# Molecular evolution of GDP-L-galactose phosphorylase, a key regulatory gene in plant ascorbate biosynthesis

**DOI:** 10.1093/aobpla/plaa055

**Published:** 2020-11-04

**Authors:** Junjie Tao, Zhuan Hao, Chunhui Huang

**Affiliations:** 1 College of Agronomy, Jiangxi Agricultural University, Nanchang, China; 2 Institute of Kiwifruit, Jiangxi Agricultural University, Nanchang, China; 3 College of Chemistry and Materials, Weinan Normal University, Weinan, China

**Keywords:** AsA, ascorbate, gene duplication, *GGP*, L-galactose pathway, molecular evolution, *VTC2*

## Abstract

Ascorbic acid (AsA) is a widespread antioxidant in living organisms, and plays essential roles in the growth and development of animals and plants as well as in the response to abiotic stress tolerance. The GDP-L-galactose phosphorylase (*GGP*) is a key regulatory gene in plant AsA biosynthesis that can regulate the concentration of AsA at the transcriptional and translational levels. The function and regulation mechanisms of *GGP* have been well understood; however, the molecular evolutionary patterns of the gene remain unclear. In this study, a total of 149 homologous sequences of *GGP* were sampled from 71 plant species covering the major groups of Viridiplantae, and the phylogenetic relationships, gene duplication and molecular evolution analyses of the genes were systematically investigated. Results showed that *GGP* genes are present throughout the plant kingdom and five shared whole-genome duplications and several lineage-specific whole-genome duplications were found, which led to the rapid expansion of *GGPs* in seed plants, especially in angiosperms. The structure of *GGP* genes was more conserved in land plants, but varied greatly in green algae, indicating that *GGP* may have undergone great differentiation in the early stages of plant evolution. Most GGP proteins had a conserved motif arrangement and composition, suggesting that plant GGPs have similar catalytic functions. Molecular evolutionary analyses showed that *GGP* genes were predominated by purifying selection, indicating that the gene is functionally conserved due to its vital importance in AsA biosynthesis. Most of the branches under positive selection identified by the branch-site model were mainly in the chlorophytes lineage, indicating episodic diversifying selection may contribute to the evolution of *GGPs*, especially in the chlorophyte lineage. The conserved function of *GGP* and its rapid expansion in angiosperms maybe one of the reasons for the increase of AsA content in angiosperms, enabling angiosperms to adapt to changing environments.

## Teaser

The molecular evolutionary patterns of plant *GGP* genes, which play a key regulatory role in AsA biosynthesis, were explored in this study. Most GGPs had similar gene structure and motif patterns, indicating plant GGPs have conserved functions. Molecular evolutionary studies showed that GGPs were mainly constrained by purifying selection, indicating the functional importance of GGPs. A few branches were identified under positive selection, indicating that episodic diversifying selection played a role during the evolution of plant GGPs. Several shared WGDs and lineage-specific WGDs were identified in seed plants, which may have promoted the radiation of *GGP* gene in angiosperms.

## Introduction

L-ascorbic acid (AsA), also well-known as ascorbate or Vitamin C (Vc), is a water-soluble vitamin and an essential micronutrient for the normal growth and development of both animals and plants. As a major antioxidant, AsA can protect cells in living organisms from the threat of reactive oxygen species (ROS) under abiotic stress. At the same time, AsA is also a cofactor for dioxygenase and plays a vital role in most metabolic processes (Macknight *et al.* 2017). Ascorbic acid is present in a wide range of plant tissues, and is a multifunctional metabolite linked to many physiological processes like regulating photosynthesis, growth and development, cell wall biosynthesis, regulating seed germination, flowering time, fruit softening and aging, postharvest storage, mediating signal transduction and enhancing plant resistance to adverse environments (Gallie 2013; Mellidou *et al.* 2017; Fenech *et al.* 2018). Lack of AsA in the human body can lead to scurvy and other diseases, while an appropriate amount of AsA is beneficial to prevention of aging, cancer and other diseases (Camarena and Wang 2016; Magrì *et al.* 2020). However, due to several mutations in the gene encoding L-gulonolactone oxidase (GuLO) in AsA synthesis, human beings and some mammals have lost the ability to synthesize AsA by themselves (Nishikimi *et al.* 1994). As a result, in order to meet daily requirements, humans have to secure the required AsA from plants, especially fresh fruits and vegetables that contain high levels of AsA. In view of the unique functions and importance of AsA in normal life activities of plants and animals, it is of great interest to study the biosynthesis and regulation of AsA in plants.

**Figure 1.**
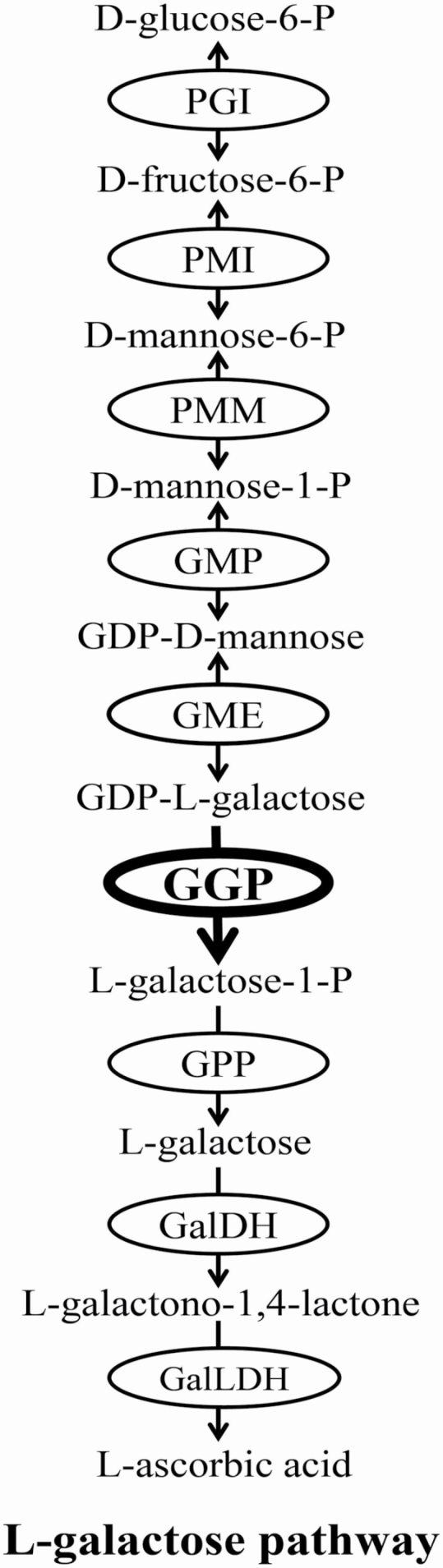
Ascorbic acid biosynthesis by the L-galactose pathway in plants. Enzymes involved in L-galactose pathway are labelled in the circles, including (1) PGI: glucose-6-phosphate isomerase; (2) PMI: mannose-6-phosphate isomerase; (3) PMM: phosphomannomutase; (4) GMP: GDP-mannose pyrophosphorylase; (5) GME: GDP-mannose-3′,5′-epimerase; (6) GGP: GDP-L-galactose phosphorylase; (7) GPP: L-galactose-1-phosphate phosphatase; (8) GalDH: L-galactose dehydrogenase; (9) GalLDH: L-galactono-1,4-lactone dehydrogenase.

Four biosynthetic pathways to AsA have been proposed in plants: the L-galactose pathway (Wheeler *et al.* 1998), the L-glucose pathway (Wolucka and Van Montagu 2003), the D-galacturonic acid (Agius *et al.* 2003) and the myo-inositol pathway (Lorence *et al.* 2004). The L-galactose pathway, also named as the Smirnoff–Wheeler pathway, is the best established AsA biosynthesis pathway in plants and considered to be the only predominant pathway for AsA accumulation in most plant species, such as vascular plants, mosses and green algae (Ishikawa *et al.* 2018). The L-galactose biosynthesis pathway starts from D-glucose-6-P and involves a total of nine steps of enzymatic reaction (Fig. 1). All the enzymes and the corresponding coding genes involved in this biosynthetic pathway have been identified and well characterized in several higher plants (Bulley and Laing 2016).

GDP-L-galactose phosphorylase (GGP), which catalyses the generation of L-galactose-1-P from GDP-L-galactose, is the first committed step in L-galactose biosynthesis pathway of AsA in many plants (Bulley and Laing 2016). The function of GGP was not discovered until 2007, and the gene encoding GGP was the last gene cloned from the L-galactose pathway (Laing *et al.* 2007; Linster *et al.* 2007). Since then, *GGP* genes have been identified and functionally characterized in several plant species, such as kiwifruit (*Actinidia chinensis*) (Bulley *et al.* 2009), apple (*Malus* × *domestica*) (Mellidou *et al.* 2012a), tomato (*Solanum lycopersicum*) (Wang *et al.* 2014) and blueberry (*Vaccinium corymbosum*) (Liu *et al.* 2015). In some plant genomes, GGP proteins are usually encoded by multiple homologous genes, such as two (*VTC2* and *VTC5*) and three (*MdGGP1*, *MdGGP2* and *MdGGP3*) homologous genes encoding GGP were identified in *Arabidopsis thaliana* and apple (*Malus* × *domestica*), respectively (Dowdle *et al.* 2007; Mellidou *et al.* 2012a). Sequence comparison reveals that VTC2 and VTC5 belong to the histidine triad (HIT) protein superfamily and can specifically catalyse the conversion of GDP-L-galactose to L-galactose-1-phosphate (Dowdle *et al.* 2007). The expressions of *VTC2* and *VTC5* are regulated by light and could be detected throughout the whole growth and development stages and in almost all tissues (root, stem, leaf, flower and silique) of *A. thaliana*, and the expression level in green tissues is significantly higher than that in roots (Dowdle *et al.* 2007; Müller-Moulé 2008). VTC2 and VTC5 are both hydrophilic proteins without transmembrane domains and organelle localization sequence (Dowdle *et al.* 2007). Subcellular localization studies showed that *A. thaliana* VTC2 and *S. lycopersicum* GGP exist in cytoplasm and nucleolus, suggesting that plant GGP may be a dual-function protein with enzymatic and regulatory functions (Müller-Moulé 2008; Wang *et al.* 2013).

*GGP* is a critical step in regulating the biosynthesis of AsA in plants, and can control AsA biosynthesis at the transcriptional and translational levels. The expression level of the *GGP* gene has been found to be closely related to the content of AsA in plants, for instance in kiwi (*Actinidia* spp.) (Bulley *et al.* 2009), tomato (*S. lycopersicum*) (Wang *et al.* 2013) and blueberry (*V. corymbosum*) (Liu *et al.* 2015). Conversely, suppression of *GGP* may lead to decrease in AsA levels (Bulley *et al.* 2009; Bulley *et al.* 2012; Wang *et al.* 2013). These studies suggest that *GGP* is a major control point of AsA biosynthesis in plants. At the translational level, a highly conserved upstream open reading frame (uORF) in the 5′ untranslated region (UTR) of *GGP* regulates AsA biosynthesis by forming a feedback loop. The uORF structure regulates the concentration of AsA and the translation of GGP. Under high concentration of AsA, the uORF is translated and inhibits the translation of *GGP*, while under low concentration of AsA, the uORF will not be translated and *GGP* can be smoothly translated to synthesize AsA (Laing *et al.* 2015). Genome editing of the uORF of *LsGGP2* in *Lactuca sativa* can significantly increase the concentration of AsA in lettuce leaves, and thus can also improve the tolerance of plants to oxidative stress (Zhang *et al.* 2018). Similar results were also obtained by editing the uORF of *SlGGP1* in tomato (*S. lycopersicum*) (Li *et al.* 2018). The feedback regulation of AsA biosynthesis suggests that the regulation mechanism at the translation level also plays an important role in the biosynthesis of AsA.

In view of the important functions of AsA in maintaining normal life activities in almost all living organisms, the AsA biosynthesis pathways and the corresponding structural genes, especially the control points such as the *GME* and *GGP*, have received much attention in recent years. As the first committed step of AsA biosynthesis pathway, *GGP* has attracted particular attention and has been widely investigated. At present, its physical and chemical properties, expression characteristics, and roles in plant AsA accumulation and biosynthesis have been well understood. However, the evolutionary patterns and functional divergences of plant *GGP* genes are still unclear. In this study, 149 homologous sequences of *GGP* genes were sampled from 71 plant species representing the major groups of Viridiplantae, and their phylogenetic relationships, gene duplication and molecular evolution analyses were first investigated systematically. The results of this study will shed light on the evolutionary patterns of plant *GGP* genes and help to further understand the biological functions of the gene in plant AsA biosynthesis.

## Methods

### Acquisition and characterization of plant *GGP* coding sequences

In order to explore and better understand the evolutionary patterns of plant *GGP* genes, comprehensive homology searches based on the BLAST method were performed (Altschul *et al.* 1990). The amino acid sequences, genomic sequences and coding DNA sequences (CDS) of plant *GGP* genes used in this study were collected from the online databases of Phytozome v12.1 (https://phytozome.jgi.doe.gov/pz/portal.html), the National Center for Biotechnology Information (NCBI) (https://www.ncbi.nlm.nih.gov/), and a selection of genomes from ConGenIE (http://congenie.org/citation) and DRYAD (https://doi.org/10.5061/dryad.0vm37) (Wan *et al.* 2018a). As a model plant, the number and function of genes involved in Vc biosynthesis pathway in *A. thaliana* have been well studied. Therefore, the *A. thaliana* GGP amino acid sequences of VTC2 (At4g26850) and VTC5 (At5g55120), which were downloaded from the TAIR database (https://www.arabidopsis.org/), were used as queries to carry out BLASTP searches against the databases of Phytozome v12.1 and NCBI with default algorithm parameters to identify GGP coding sequences in Viridiplantae. To obtain *GGP* coding sequences from the gymnosperm lineage, we also performed BLASTP searches using the VTC2 and VTC5 protein sequences against the genomes of *Picea abies*, *Pinus taeda* (both in the ConGenIE database) and *Gnetum montanum* (downloaded from the DRYAD website). All identical, redundant, partial and incomplete sequences were manually identified and eliminated from the original sequences using the BioEdit v7.1.13 software (Hall 1999), and only the full-length coding sequences were retained in the final data set.

### Multiple sequence alignment, gene structures and protein motifs analyses

Amino acid sequences of the collected plant GGP were firstly aligned using MAFFT program v7.158 (Katoh and Standley 2013) with default parameters. After manually curated in BioEdit, the multiple sequence alignment of the amino acid sequences and the corresponding unaligned CDS sequences of plant *GGP* genes were uploaded to PAL2NAL website (http://www.bork.embl.de/pal2nal/) (Suyama *et al.* 2006) and then converted into the coding sequence alignment. Subsequently, the codon alignment was filtered using the program Gblocks v0.91b (Castresana 2000) to trim ambiguously aligned positions and to obtain conserved regions, with 50 % gapped positions in the alignment were allowed and all other parameters were kept at default options.

The Gene Structure Display Server v2.0 (GSDS) (http://gsds.cbi.pku.edu.cn/) (Hu *et al.* 2015) online tool was employed to display the exon–intron structure features of plant *GGP* genes by comparing the original full-length CDS sequences with their corresponding genomic sequences. Moreover, the motif analysis tool of Multiple Em for Motif Elicitation v5.0.5 (MEME) (http://meme-suite.org/tools/meme) (Bailey *et al.* 2009) was used to detect conserved motif structures of plant GGP protein sequences with mostly default parameters except for the number of motifs was set to 10.

### Detection of recombination events

It is well-known that recombination events may adversely affect the accuracy and efficiency of phylogenetic reconstruction and molecular evolutionary analysis (Posada and Crandall 2002; Anisimova *et al.* 2003; Shriner *et al.* 2003). As a result, to avoid the potential impact of recombination on our data set of plant *GGP* protein-coding DNA sequences, the GARD recombination detection method (Kosakovsky Pond *et al.* 2006) implemented in Datamonkey web server (http://www.datamonkey.org/) (Weaver *et al.* 2018) was initially utilized to screen for evidence of recombination breakpoints prior to phylogenetic and evolutionary analyses.

### Gene tree reconstruction

The nucleotide gene tree of plant *GGPs* was generated by Bayesian inference implemented in the program MrBayes v3.2.6 (Ronquist *et al.* 2012), and no outgroups were used in the construction of the gene tree. Prior to reconstruct the Bayesian phylogeny, the best-fit nucleotide substitution model of GTR+I+G was determined using MrModeltest v2.3 under the standard of Akaike Information Criterion (AIC) (Nylander 2008). The Bayesian phylogenetic reconstruction was run for 10 000 000 Markov Chain Monte Carlo (MCMC) generations and sampled every 100 generations. Trees from the first 25 % of the sampled generations were discarded as burn-in. The final gene tree was edited and visualized using iTOL web server (https://itol.embl.de/) (Letunic and Bork 2016).

### Molecular evolutionary analyses

To test for signatures of natural selection in plant *GGP* genes, several codon-based maximum likelihood models implemented in the codeml program in the PAML package v4.9i (Yang 2007) were used in this study. And the aligned codon-based sequences and the reconstructed Bayesian phylogenetic tree were fed into the codeml program to estimate the non-synonymous (*d*_N_) versus synonymous substitution (*d*_S_) rate ratio (ω = *d*_N_/*d*_S_). The ω values estimated by the maximum likelihood methods is a useful measurement to identify adaptive molecular evolution, with ω = 1, <1 and >1 meaning neutral evolution, purifying selection and positive selection, respectively (Yang *et al.* 2000). Missing data were treated as ambiguity nucleotides or amino acids within codeml. Due to the large data set in this study, we first estimated the branch lengths under the model M0 (one-ratio model), then the tree with branch lengths from the main output file of M0 was used as tree file when run other models. The analyses of codeml were run several times with different initial parameter values to evaluate the convergence.

To test the variation of ω between amino acid sites and identify potential sites evolving by positive selection, three pairs of site-specific models were compared, including M0 (one-ratio model) versus M3 (discrete model), M1a (nearly neutral model) versus M2a (positive selection model) and M7 (neutral, β model) versus M8 (selection, β and ω model) (Yang *et al.* 2000). The one-ratio model M0 assumes a constant ω ratio for all sites and all branches, whereas the discrete model M3 assumes a discretized distribution of ω ratios. The nearly neutral model M1a allows sites with ω ≤ 1, while the positive selection model M2a adds an additional class of sites with ω > 1. The neutral model M7 assumes the β distribution of ω values among sites, whereas the alternative selection model M8 adds an extra category of sites with ω > 1 to the model M7. The comparison of the three pairs of models was performed through likelihood-ratio test (LRT) with chi-square (χ ^2^) distribution. If the LRT was significant (*P*-value < 0.01), then the Bayes Empirical Bayes (BEB) (Yang *et al.* 2005) approach was employed to identify amino acid sites under positive selection (posterior probability ≥ 90 %).

To test for different ω among lineages, we used the branch and branch-site models implemented in codeml. The two-ratio model (a branch model) was used to evaluate differences in selection pressures among lineages of particular interest (e.g. those that had experienced duplication events like the angiosperms lineage), while the improved branch-site model (Zhang *et al.* 2005) was used to test for positive selection along particular branches and sites (e.g. along the main lineages of Viridiplantae). For the two-ratio model and the branch-site model analyses, the lineages or branches of interest were prespecified as foreground branches that allow positive selection, while the rest of the lineages or branches were defined as background branches that allow negative or neutral selection. The LRT was again used to evaluate how well the data fitted the alternative model (allowing positive selection on the foreground branch) compared to the simpler model not allowing positive selection on the foreground branch. In addition, the Bonferroni’s correction was employed to control the family-wise error rate when multiple branches on the phylogeny were used to detect positive selection in the branch-site test (Anisimova and Yang 2007).

## Results

### Identification of *GGP* genes in the plant kingdom

In total, 149 homologous sequences encoding putative GGPs were mined from 71 Viridiplantae species in the final data set **[see**  **Supporting Information—Table S1**, **Text S1****]**. These species, including 15 monocots and 41 eudicots, 4 gymnosperms, 1 lycophytes, 3 bryophytes and 7 chlorophytes, represented the main lineages of Viridiplantae. The BLAST results also indicated that the *GGP* gene exists widely in various plants.

A considerably variable number of the *GGP* genes was observed among the tested Viridiplantae species **[see**  **Supporting Information—Table S1****]**. Most plant species in lineages of eudicots, monocots, gymnopsperms, lycophytes and bryophytes contained at least two homologues of *GGP*, and the highest copy number of five was found in the eudicot species of *Eucalyptus grandis* and the gymnosperm species of *P. taeda*. In a few species, especially in the lineage of chlorophytes, only one copy of the *GGP* gene was found. The CDS length of plant *GGP* genes ranged from 957 to 1854 bp, and the overall percentage of missing data is between 13.2 and 50.8 %. Positions with a gap in <50% in the final sequence alignment were reserved.

### Recombination test and phylogenetic analysis of plant *GGP* genes

No evidence of recombination event was found according to the result of GARD. Therefore, the alignment of plant *GGP* genes could be directly used to reconstruct phylogenetic relationships and perform molecular evolutionary analysis.

**Figure 2.**
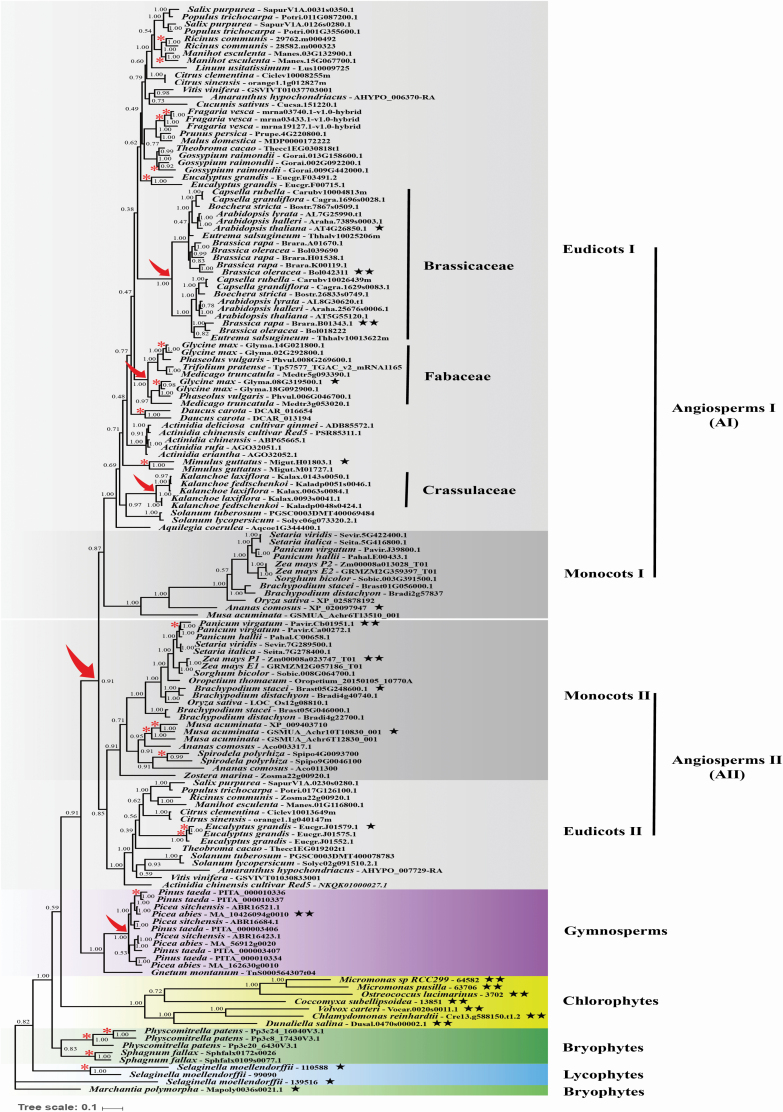
Phylogenetic analyses of plant *GGP* genes using the Bayesian method. The phylogenetic tree was constructed through the Bayesian method under the GTR+I+G model. Posterior probabilities are labelled near the nodes. The accession number of the *GGP* gene is listed after the name of the species. Red arrows indicate shared WGDs. Red asterisks (*) indicate lineage-specific WGDs. Black star (★) indicates the branch is identified under episodic diversifying selection by branch-site model. Double black stars (★★) indicate that the branch is still under positive selection after Bonferroni correction.

A phylogenetic tree of plant *GGP* was constructed from the alignment of nucleotide sequences using Bayesian method. The plant species used in this study involves the main lineages of Viridiplantae, including angiosperms, gymnosperms, lycophytes, bryophytes and chlorophytes. The positions of these major lineages in the constructed gene tree are basically consistent with the already published phylogenies of Viridiplantae. The resulting Bayesian phylogenetic tree showed that *GGP* genes from angiosperms (including 87 eudicot sequences and 34 monocot sequences) formed a single lineage with high posterior probability support (Fig. 2). Except the bryophyte gene sequences, which were divided into two separate clades, other sequences from gymnosperms, chlorophytes and lycophytes all formed a single lineage with high posterior probabilities, respectively (Fig. 2).

In the angiosperms lineage, one shared whole-genome duplication (WGD) was found prior to the radiation of angiosperms, resulting in two subclades of angiosperm I (AI) and angiosperm II (AII) with posterior probability values >0.85, and each of the two subclades contained monocotyledon and dicotyledon *GGP* gene sequences (Fig. 2). Furthermore, another three shared WGDs could also be identified in the eudicots I of AI subclade, which occurred before the radiation of Brassicaceae, Fabaceae and Crassulaceae with strongly posterior probability support, respectively, leading to the expansion of these three families (Fig. 2). Besides, a major duplication event could also be found within the lineage of gymnosperms with high posterior probability support (Fig. 2). Except the shared WGDs, several lineage-specific WGDs could also be found in the phylogenetic tree, such as *Ricinus communis*, *Manihot esculenta*, *Fragaria vesca*, *Gossypium raimondii*, *E. grandis*, *Glycine max*, *Daucus carota*, *Mimulus guttatus* in eudicots, *Panicum virgatum*, *Musa acuminata* and *Spirodela polyrhiza* in monocots and *P. taeda* in gymnosperms. All of these lineage-specific WGDs had high posterior probability values >0.9 (Fig. 2).

### Gene structures and conserved motifs of *GGP* genes

**Figure 3.**
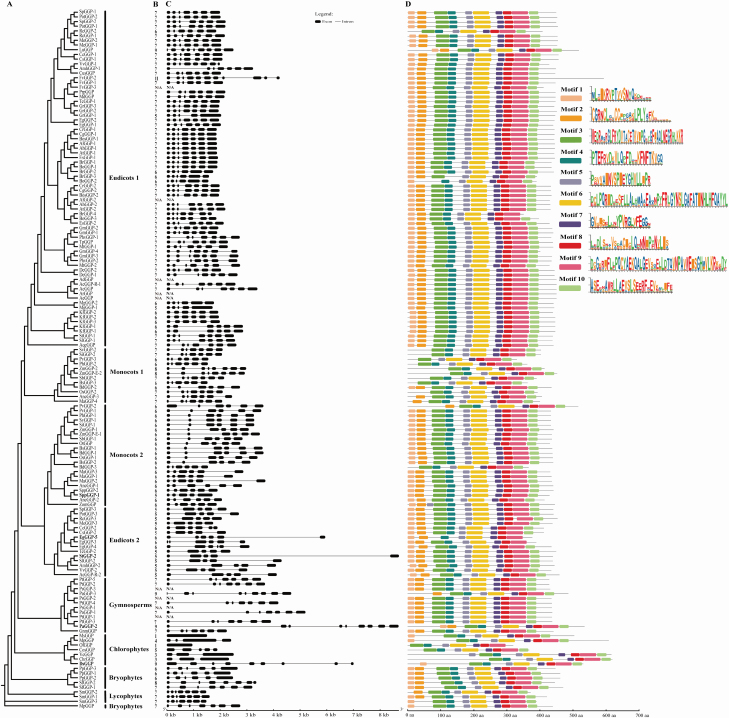
Phylogenetic relationships, gene structures and conserved protein motifs of plant *GGP* genes. (A) The Bayesian phylogenetic tree of plant *GGP* genes. (B) Exon number of corresponding *GGP* genes. (C) Exon–intron structure of plant *GGP* genes. (D) The conserved motif composition and distribution of plant GGP proteins. The conserved motifs are displayed in different coloured boxes, and the sequence information for each motif is displayed in the form of seqlog.

The exon–intron structure of plant *GGP* genes is illustrated in Fig. 3. As the genomic sequences of some genes, like *FvGGP-3* (*F. vesca*), *AlGGP-2* (*Arabidopsis lyrata*), *AdGGP* (*Actinidia deliciosa*), *ArGGP* (*Actinidia rufa*), *AeGGP* (*Actinidia eriantha*), *PsGGP* (*Picea sitchensis*) and *PtGGP-1* (*P. taeda*), were not available at the moment, their exon–intron structures were not examined in this study. As shown in Fig. 3B and C, the number of exons varied greatly among different genes, generally ranging from 1 to 11. However, most of the plant *GGP* genes share a similar exon–intron organization, and more importantly, genes within the same lineage usually have the same exon–intron organization. For example, genes within the eudicots I lineage varied from 5 to 11 exons, while most of them (73.6 %) contained 7 exons. The exon numbers of genes within the lineages of monocots I, monocots II, eudicots II, gymnosperms, chlorophytes, bryophytes and lycophytes contained 6–8, 5–6, 5–6, 7–9, 1–9, 6–8 and 7 exons, respectively. In the lineage monocots II, all of the genes contained 6 exons except for *SppGGP-1* (*S. polyrhiza*), which contained only 5 exons. This may be due to the loss of the fifth intron in *SppGGP-1* gene. Compared with the genes in other lineages, the exons-intron structure of the majority genes in chlorophytes varied greatly, and two intron-less genes and one gene with 9 exons were found in this lineage (Fig. 3B). Furthermore, a large divergence of intron length was observed in a few genes, such as *EgGGP-5* (*E. grandis*) and *StGGP-2* (*Solanum tuberosum*) in eudicots II, *PaGGP-2* (*P. abies*) in gymnosperms and *DsGGP* (*Dunaliella salina*) in chlorophytes contained several extremely long introns, which were significantly longer than other genes (Fig. 3C).

To investigate the structural divergences and the structural evolution of plant GGP proteins, the conserved motifs were estimated using the MEME online tool. As exhibited in Fig. 3D, a total of 10 conserved motifs were identified and the motifs were present in almost all sequences. Motif compositions and distributions were found to be conserved in most plant GGP proteins sequences, especially within the same lineage members. Some motifs were found to be lacking in a few GGP sequences. For example, motif 1 and 2 lacked in RcGGP-2 in eudicots I, SvGGP-2 and SiGGP-2 in monocots I, and BdGGP-3 in monocots II, motif 2 and 3 lacked in BoGGP-2 in eudicots I, motif 3 and 10 lacked in FgGGP-5 in eudicots II. Notably, motif structural and distribution divergences mainly occurred in the lineage of monocots I and chlorophytes, especially in chlorophytes where almost all members in this lineage lacked at least one motif (Fig. 3D).

### Molecular evolutionary analysis of plant *GGP* genes

Different likelihood-based methods implemented in codeml from the PAML package of programs were used to assess the type and strength of natural selection acting on plant *GGP* genes. The branch models were firstly used to test the variation of selective pressure among different branches of the phylogeny tree. The one-ratio model M0, which assumes a single ω across all branches and sites in the phylogeny, estimated the ω _0_ value for plant *GGP* genes was 0.09302 (Table 1), suggesting that the evolution of *GGP* genes was predominated by strong purifying selection. A large-scale duplication event was identified in the angiosperm lineage, which gave rise to the angiosperm lineage to split into two sublineages of angiosperm I (AI) and angiosperm II (AII) (Fig. 2). The lineage-specific two-ratio model was employed to detect the changes of selection pressures between different lineages after the duplication event, and the ancestral branches leading to angiosperm, angiosperm I, angiosperm II, eudicots I, monocots I, eudicots II and monocots II were set as foreground branch, separately. The results of two-ratio model analyses were given in Table 1. For the ancestral branch leading to angiosperm as foreground branch, the estimated ω value was lower than that of background value; however, the LRT statistic result showed that the two-ratio model did not better fit than the null model M0 (Table 1), indicating the selection pressure after the duplication event has not changed significantly. For the comparison between the two-ratio model and the one-ratio model, only the ancestral branches leading to eudicots I and monocots II were found significantly different from their background branches (Table 1). In general, these results indicated that selection pressures experienced by different lineages were different after the duplication of angiosperm, and *GGP* genes in angiosperm II may be subjected to more relaxed selection constraints during evolution.

**Table 1.** PAML branch model analyses to test the variable selective pressure among branches and after gene duplication. ^a^Np: number of estimated parameters. ^b^lnL: log-likelihood scores. ^c^df: degree of freedom. ^d^−2ΔlnL: twice the log-likelihood difference of the models being compared. *P < 0.05; **P < 0.01.

Model	Np^a^	lnL^b^	Parameter estimates	Models compared	df^c^	−2ΔlnL^d^	*P-*value
A: One-ratio model M0	297	−49065.194200	ω _0_ = 0.09302				
B: Two ratios (angiosperm)	298	−49063.900889	ω _0_ = 0.09333, ω _angiosperm_ = 0.04308	B vs. A	1	2.586622	0.1078
C: Two ratios (angiosperm I)	298	−49063.877071	ω _0_ = 0.09272, ω _angiosperm I_ = 949.49270	C vs. A	1	2.634258	0.1046
D: Two ratios (angiosperm II)	298	−49064.939231	ω _0_ = 0.09317, ω _angiosperm II_ = 0.06067	D vs. A	1	0.509938	0.4752
E: Two ratios (eudicots I)	298	−49061.885830	ω _0_ = 0.09241, ω _eudicots I_ = 1.65002	E vs. A	1	6.61674*	0.0101
F: Two ratios (monocots I)	298	−49063.571050	ω _0_ = 0.09264, ω _monocots I_ = 0.20233	F vs. A	1	3.2463	0.0716
G: Two ratios (eudicots II)	298	−49065.190239	ω _0_ = 0.09304, ω _eudicots II_ = 0.08908	G vs. A	1	0.007922	0.9291
H: Two ratios (monocots II)	298	−49060.105366	ω _0_ = 0.09359, ω _monocots II_ = 0.02125	H vs. A	1	10.177668**	0.0014

Site-specific codon models were then applied to explore ω value variation across different codon sites and identify potential sites under positive selection. The comparison between M0 and M3 showed that M3 fits the data significantly better than the M0 model (−2ΔlnL = 2937.632, *P* < 0.0001), suggesting that ω values were not homogeneous across different sites. However, the positive selection models of M2a and M8 did not fit the data significantly better than their corresponding negative models of M1a and M7, respectively, and failed to identify any sites under positive selection **[see**  **Supporting Information—Table S2****]**.

The more powerful branch-site models were also applied to test for episodic positive selection acting on a subset of sites along specific branches. First of all, the main lineages of angiosperm, angiosperm I, angiosperm II, eudicots I, eudicots II, monocots I, monocots II, gymnosperms, chlorophytes, bryophytes and lycophytes were assigned as foreground branches, respectively. The LRTs showed that no significant evidence of positive selection was detected in those lineages **[see**  **Supporting Information—Table S3****]**. Then, to test whether a particular branch in the Bayesian phylogenetic tree was under positive selection, each branch in the phylogenetic tree was assigned as foreground branch and the remaining branches as background branch. The LRTs detected evidence of positive selection on 22 branches as shown in **Supporting Information—Table S4**, and the positively selected branches were labelled in the phylogenetic tree as shown in Fig. 2. However, only 12 branches, mainly distributed in lineages of eudicots I (two species), monocots II (two species), gymnosperms (one species) and chlorophytes (seven species), were under positive selection after Bonferroni correction was applied for multiple tests (Fig. 2; **see**  **Supporting Information—Table S4**). Notably, varying numbers of putative positively selected amino acid sites with posterior probability >0.95 under BEB level on these branches were identified as shown in **Supporting Information—Table S4**.

## Discussion

As a rate-limiting step in L-galactose pathway in both green algae and higher plants, *GGP* plays an essential role in plant AsA biosynthesis and the expression level of *GGP* largely determines the synthesis rate of AsA (Vidal-Meireles *et al.* 2017). In this study, 147 sequences of *GGP* homologues were retrieved from 71 plant species, representing major Viridiplantae lineages including eudicots, monocots, gymnosperms, lycophytes, bryophytes and chlorophytes, and the functional diversity and evolutionary patterns were systematically explored.

The plant *GGP* gene has undergone several duplication events during its evolution. Among the 71 plant species collected in this study, 50 species contained more than two copies of *GGP*, which were mainly distributed in the lineage of angiosperms and gymnosperms, while the species containing only one copy of *GGP* gene were found mainly in the lineage of chlorophytes. Phylogenetic analyses revealed five well-supported shared WGDs in the evolutionary history of plant *GGP* genes. Gene duplication, which leads to an increase in the number of gene copies, usually comes from WGD events. WGDs occurred multiple rounds during the long-term evolutionary process of seed plants, which greatly promoted the adaptive radiation of seed plants (Murat *et al.* 2017; Van de Peer *et al.* 2017; Ren *et al.* 2018; Wan *et al.* 2018b). In this study, five shared WGDs were identified, four of which occurred in the lineage of angiosperms and coincided with WGD events previously identified in angiosperms. The first shared WGD occurred in the angiosperm ancestral species, resulting in two sublineages of angiosperm I and angiosperm II (Fig. 2). The other three duplication events were Brassicaceae, Fabaceae and Crassulaceae specific, respectively, and all occurred in eudicots I in the sublineage of angiosperm I (Fig. 2). The three gene duplication events coincided with WGD events in the Brassicales (Barker *et al.* 2009; Donoghue *et al.* 2011), Fabaceae (Schmutz *et al.* 2010; Young *et al.* 2011; Tang *et al.* 2014) and Crassulaceae (Yang *et al.* 2017), respectively. The last major gene duplication event was identified in the lineage of gymnosperms, but only in the Pinaceae lineage. This result was consistent with previous studies on early genome duplications in gymnosperms, that is, WGD events were detected in Pinaceae and other gymnosperms, while no evidence of WGDs was detected in the genome of gnetophytes (Li *et al.* 2015; Wan *et al.* 2018b). Moreover, a number of lineage-specific WGDs were also identified frequently in the seed plant lineages (Fig. 2). The five shared WGDs and a number of lineage-specific WGDs led to the rapid expansion of *GGP* genes in seed plants, especially in angiosperms. In general, the concentration of AsA in higher plants is usually much higher than that in bryophytes and green algae (Gest *et al.* 2013; Vidal-Meireles *et al.* 2017). For example, AsA concentrations in higher plants range approximately from 2 to 135 μmol g^−1^ FW (fresh weight); however, green algae species of *Ulva compressa* and bryophyte species of *Hypnum plumaeforme* exhibit AsA concentrations of ~0.5 μmol g^−1^ FW and 0.1–0.6 μmol g^−1^ FW, respectively (Gest *et al.* 2013; Tao *et al.* 2018). The relationship between the increased copy number of *GGP* gene and the higher AsA content in angiosperms remains to be further studied.

Most plant *GGP* genes have similar exon–intron structure and relatively conservative motif composition and distribution. The structure of *GGP* gene was more conserved in land plants, but varied greatly in green algae, indicating that *GGP* may have undergone great differentiation in the green algae lineage. Most GGP proteins had a conserved motif arrangement and composition, suggesting that plant GGPs have similar catalytic functions. Nevertheless, there may be some differences in the expression patterns and functions of *GGP* homologues in the same plant. For example, *VTC2* and *VTC5* both encode GGP in *A. thaliana*, but their expression levels and tissues specificity are a bit different, with *VTC2* playing a more important role in AsA biosynthesis (Dowdle *et al.* 2007). Studies in tomatoes (*S. lycopersicum*) showed that although *SlGGP2* played a role in regulating the concentration of AsA in fruit, the expression level of *SlGGP1* was more closely related to the level of AsA during fruit ripening (Mellidou *et al.* 2012b). Studies on the *LsGGP1* and *LsGGP2* uORF mutants in lettuce also revealed functional differences between the two isozymes, suggesting that *LsGGP2* may be the major GGP isoenzyme that regulates AsA biosynthesis (Zhang *et al.* 2018).

*GGP* is generally considered as a major determinant gene in plant AsA biosynthesis, and plays an important role in regulating AsA concentrations in many plants. Although the one-ratio model M0 is not a very realistic model to detect adaptive evolution, it is still widely used to estimate the selective pressure acting on genes (Yang *et al.* 2009; Montanucci *et al.* 2011; Darfour-Oduro *et al.* 2016). In this study, evolutionary analysis revealed that plant *GGP* gene was mainly restricted by purifying selection (ω _0_ = 0.09302), which indicated the functional importance and conservativeness of plant *GGP* genes during evolution. The molecular evolutionary results of *GGP* were similar to that of *GME*, which is the upstream gene of *GGP* in L-galactose pathway and is also considered as a key gene in plant AsA biosynthesis, and also had undergone strong purifying selection during evolution (ω _0_ = 0.0287) (Tao *et al.* 2018). Moreover, a total of 22 branches were identified under positive selection. Even after Bonferroni correction, there were still 12 branches under positive selection, most of which (seven branches) were in the chlorophytes lineage. These results were also consistent with the results in the *GME*, where most of the positively selected branches detected in the *GME* species were located in the green algae lineage (Tao *et al.* 2018), and also suggesting that the evolutionary innovation of *GGP* genes may play an important role in helping plants adapt to new and challenging environments such as high light, high altitude, UV, low temperature and aquatic environments (Gest *et al.* 2013).

In plants, the L-galactose pathway involves nine consecutive enzymes, of which *GME* and *GGP* are considered to be the critical steps to regulate the synthesis of AsA. The expression of *GME* and *GGP* is induced by light and abiotic stress, and these two genes operate synergistically to regulate AsA biosynthesis (Bulley and Laing 2016; Mellidou and Kanellis 2017). At present, only the evolutionary patterns of *GME* and *GGP* have been studied, while the selection signatures of other genes in L-galactose pathway are not still clear, and the factors affecting the evolution rate of genes in L-galactose pathway are also uncertain. Molecular evolution studies of other genes in the L-galactose pathway in future works will help to clarify the evolution patterns of the L-galactose pathway genes and identify factors affecting the selection pressure differences among the pathway genes.

The conservative region selection is an important step in phylogenetic analysis; however, aggressive filtering may affect the accuracy of phylogenetic inference and selective pressure detection. As a result, we used accurate codon-based alignment algorithms (e.g. MAFFT) to reduce alignment error and setting appropriate parameters to retain as many residues as possible (e.g. allowed 50 % gap positions) can properly reduce the effect of alignment filtering on positive selection analyses. Major groups of plant kingdom were involved in this study, but the sampling is fairly uneven at the order of family level. For example, there are five species from Actinidiaceae, eight species from Brassicaceae, while none are from basal angiosperms. The main purpose of this study is to understand the evolutionary patterns of *GGP* genes in plants. We did not pay attention to the evolutionary differences of *GGP* genes among different families of angiosperms. Therefore, the number of species in different families may be uneven, while the difference in the number of species among different families will not affect the conclusions of this study.

In conclusion, the molecular evolutionary patterns of plant *GGP* genes, which play a key regulatory role in AsA biosynthesis, were first systematically explored in this study. Most plant *GGP* genes had similar gene structure and motif patterns, indicating that plant *GGP* genes have conserved functions. Molecular evolutionary studies showed that *GGP* genes were mainly constrained by purifying selection, which indicated the functional importance of *GGP*. A few branches were identified under positive selection and most of which located in the chlorophytes lineage, indicating that episodic diversifying selection played a role during the evolution of plant *GGP* genes. Several shared WGDs and lineage-specific WGDs were identified in seed plants, especially in angiosperm lineages, which may promote the radiation of *GGP* gene in angiosperms.

## Supporting Information

The following additional information is available in the online version of this article—

**Text S1.** Alignment of plant GGP protein sequences and the histidine triad (HIT) motif is marked using box.

**Table S1.** Plant *GGP* genes used in this study.

**Table S2.** Results of site models for detection of positively selected sites in plant *GGP* genes.

**Table S3.** Results of branch-site test by treating each main lineages in the phylogeny as the foreground branch.

**Table S4.** Results of branch-site tests by treating each branch in the phylogeny as the foreground branch.

plaa055_suppl_Supplementary_Material

plaa055_suppl_Supplementary_Table_S1

## Data Availability

The amino acid sequences, genomic sequences and coding DNA sequences (CDS) of plant *GGP* were mainly downloaded from online databases, including Phytozome v12.1 (https://phytozome.jgi.doe.gov/pz/portal.html), NCBI (https://www.ncbi.nlm.nih.gov/), ConGenIE database (http://congenie.org/citation) and DRYAD website (https://doi.org/10.5061/dryad.0vm37). The detailed information of plant *GGP* genes involved in this study is shown in **Supporting Information—Table S1**.

## Notes

**Evolution & Diversity. Chief Editor**: Jeremy Beaulieu
