# Supplementary material for: Molecular evolution of GDP-L-galactose phosphorylase, a key regulatory gene in plant ascorbate biosynthesis
Source: AoB Plants. 2020 Nov 4;12(6):plaa055. doi: 10.1093/aobpla/plaa055 (PMC7640755; doi:10.1093/aobpla/plaa055)
Supplement: plaa055_suppl_Supplementary_Material [file plaa055_suppl_supplementary_material.pdf]

Table S2. Results of site models for detection of positively selected sites in plant *GGP* genes.

Model	Np[a]	lnL[b]	Parameters	Models compared	d.f.[c]	-2ΔlnL[d]	*p*-value	Positively selected sites
**M0**	297	-49065.194	ω=0.09302					none
**M3**	301	-47596.378	p0=0.38716, ω0=0.01357	M3 vs. M0	4	2937.632***	0.000	none
			P1=0.40745, ω1=0.08947					
			P2=0.20538, ω2=0.27976					
**M1a**	298	-48824.857	p_0=0.88231, ω_0=0.08702					Not allowed
			p_1=0.11769, ω_1=1.0000					
**M2a**	300	-48824.857	p_0=0.88232, ω_0=0.08702;	M2a vs. M1a	2	0	1.000	none
			p_1=0.11768 ω_1=1.000;					
			p_2=0.0, ω_2=7.98423					
**M7**	298	-47538.644	p=0.66833, q=5.72263					Not allowed
**M8**	300	-47538.647	p0=0.99999, p=0.66832	M8 vs. M7	2	0.006	0.997	none
			q=5.72257					
			p1=0.00001, ω=2.73695					

[a] Np: number of estimated parameters;

[b] lnL: log likelihood score;

[c] df : degrees of freedom;

[d] -2ΔlnL: twice the log-likelihood difference of the model compared.

***Significant at $p < 0.0001$.

Table S3. Results of branch-site test by treating each main lineages in the phylogeny as the foreground branch

Foreground branch	Model	lnL[a]	Parameter estimates	-2ΔlnL[b]	d.f.[c]	*p*-value	Positively Selected sites
**Angiosperms**	Model A	-48816.091	p0 = 0.865, p1 = 0.077 p2a = 0.054, p2b = 0.005<br>background: ω0 = 0.086, ω1 = 1.000 ω2a = 0.086, ω2b = 1.000<br>foreground: ω0 = 0.086, ω1 = 1.000, ω2a = 1.000, ω2b = 1.000	0	1	1	
	Null model	-48816.091	p0 = 0.865, p1 = 0.077, p2a = 0.054, p2b = 0.005<br>background: ω0 = 0.086, ω1 = 1.000, ω2a = 0.086, ω2b = 1.000<br>foreground: ω0 = 0.086, ω1 =1.000, ω1 = 1.000, ω2b = 1.000				
**Angiosperms 1**	Model A	-48787.112	p0 = 0.876, p1 = 0.083, p2a = 0.038, p2b = 0.004<br>background: ω0 = 0.087, ω1 = 1.000, ω2a = 0.087, ω2b = 1.000<br>foreground: ω0 = 0.087, ω1 = 1.000, ω2a = 1.000, ω2b = 1.000	0	1	1	16L (1.000); 254Q (1.000)
	Null model	-48787.112	p0 = 0.876, p1 = 0.083, p2a = 0.038, p2b = 0.004<br>background: ω0 = 0.087, ω1 = 1.000, ω2a = 0.087, ω2b = 1.000<br>foreground: ω0 = 0.087, ω1 = 1.000,				

			ω2a = 1.000, ω2b = 1.000				
**Angiosperms 2**	Model A	-48764.624	p0 = 0.839, p1 = 0.091, p2a = 0.063, p2b = 0.007<br>background: ω0 = 0.086, ω1 = 1.000, ω2a = 0.086, ω2b = 1.000<br>foreground: ω0 = 0.086, ω1 = 1.000, ω2a = 1.000, ω2b = 1.000	0	1	1	17G (0.997); 28S (0.941);35E (0.955); 45A (0.999);78Q (0.942); 82P (0.999); 107A (1.000)
	Null model	-48764.624	p0 = 0.834, p1 = 0.091, p2a = 0.063, p2b = 0.007<br>background: ω0 = 0.086, ω1 = 1.000, ω2a = 0.086, ω2b = 1.000<br>foreground: ω0 = 0.086, ω1 = 1.000, 2a = 1.000, ω2b = 1.000				
**Eudicots 1**	Model A	-48805.662	p0 = 0.857, p1 = 0.105, p2a = 0.034, p2b = 0.004<br>background: ω0 = 0.087, ω1 = 1.000, ω2a = 0.087, ω2b = 1.000<br>foreground: ω0 = 0.087, ω1 = 1.000, ω2a = 1.000, ω2b = 1.000	0	1	1	254Q (1.000)
	Null model	-48805.662	p0 = 0.857, p1 = 0.105, p2a = 0.034, p2b = 0.004<br>background: ω0 = 0.087, ω1 = 1.000, ω2a = 0.087, ω2b = 1.000<br>foreground: ω0 = 0.087, ω1 = 1.000, ω2a = 1.000, ω2b = 1.000				
**Eudicots 2**	Model A	-48783.609	p0 = 0.828, p1 = 0.100, p2a = 0.064,	0	1	1	19S (0.996); 45A (1.000);

			p2b = 0.008<br>background: ω0 = 0.086, ω1 = 1.000, ω2a = 0.086, ω2b = 1.000<br>foreground: ω0 = 0.086, ω1 = 1.000, ω2a = 1.000, ω2b = 1.000				82P (0.996);107A (0.991);<br>125Q (0.922);137E (1.000);<br>145M (1.000);155L (0.938)
	Null model	-48783.609	p0 = 0.828, p1 = 0.100, p2a = 0.064, p2b = 0.008<br>background: ω0 = 0.086, ω1 = 1.000, ω2a = 0.086, ω2b = 1.000<br>foreground: ω0 = 0.086, ω1 = 1.000, ω2a = 1.000, ω2b = 1.000			
**Monocots 1**	Model A	-48739.115	p0 = 0.715, p1 = 0.106, p2a = 0.156, p2b = 0.023<br>background: ω0 = 0.083, ω1 = 1.000, ω2a = 0.083, ω2b = 1.000<br>foreground: ω0 = 0.083, ω1 = 1.000, ω2a = 1.000, ω2b = 1.000	0	1	1	1T (1.000)；2V (0.963); 3V (0.997)；<br>5I (1.000); 6N (0.964)；7Q (0.998);<br>8- (0.999)；9- (0.929);15- (1.000);<br>18A (0.998)；25D (0.997); 27L(0.988);<br>28S (0.999); 44T (0.947); 59A (0.996);<br>60Q (0.992); 75R (0.924); 94D (0.97);<br>110V(0.999);179P(1.000);194S (1.000);<br>214N (1.000); 248A(0.998);265V (0.987);<br>287Y (1.000);288A (0.996);<br>298E (0.948);299D (0.993)
	Null model	-48739.115	p0 = 0.715, p1 = 0.106, p2a = 0.156, p2b = 0.023<br>background: ω0 = 0.083, ω1 = 1.000,			

			ω2a = 0.083, ω2b = 1.000<br>foreground: ω0 = 0.083, ω1 = 1.000,<br>ω2a = 1.000, ω2b = 1.000				
**Monocots 2**	Model A	-48813.117	p0 = 0.840, p1 = 0.110, p2a = 0.045,<br>p2b = 0.006<br>background: ω0 = 0.087, ω1 = 1.000,<br>ω2a = 0.087, ω2b = 1.000<br>foreground: ω0 = 0.087, ω1 = 1.000,<br>ω2a = 1.000, ω2b = 1.000	0	1	1	12- (0.999); 28S (1.000); 78Q (0.964)
	Null model	-48813.117	p0 = 0.840, p1 = 0.110, p2a = 0.045,<br>p2b = 0.006<br>background: ω0 = 0.087, ω1 = 1.000,<br>ω2a = 0.087, ω2b = 1.000<br>foreground: ω0 = 0.087, ω1 = 1.000,<br>ω2a = 1.000, ω2b = 1.000				
**Gymnosperms**	Model A	-48826.351	p0 = 0.829, p1 = 0.128, p2a = 0.037,<br>p2b = 0.006<br>background: ω0 = 0.087, ω1 = 1.000,<br>ω2a = 0.087, ω2b = 1.000<br>foreground: ω0 = 0.087, ω1 = 1.000,<br>ω2a = 1.000, ω2b = 1.000	0	1	1	2V (0.991); 152Y (0.995)
	Null model	-48826.351	p0 = 0.829, p1 = 0.128, p2a = 0.037,<br>p2b = 0.006<br>background: ω0 = 0.087, ω1 = 1.000,<br>ω2a = 0.087, ω2b = 1.000<br>foreground: ω0 = 0.087, ω1 = 1.000,				

			ω2a = 1.000, ω2b = 1.000				
**Chlorophytes**	Model A	-48674.379	p0 = 0.587, p1 = 0.088, p2a = 0.283, p2b = 0.042<br>background: ω0 = 0.083, ω1 = 1.000, ω2a = 0.083, ω2b = 1.000<br>foreground: ω0 = 0.083, ω1 = 1.000, ω2a = 1.000, ω2b = 1.000	0	1	1	5I (0.998); 6N (0.998); 11- (0.999); 12- (0.999);18A (0.998);19S (1.000); 22L (1.000); 47E (1.000);75R (1.000); 80L (1.000); 96Q (0.991); 101A (1.000); 135C(1.000);148V(1.000); 190G(0.998); 214N (1.000); 215A (0.999);216C (0.903); 217I (1.000);242C (1.000);245E (0.988); 249R (0.994); 251E (1.000);277R (1.000); 284T (1.000);287Y (1.000); 289K (1.000); 294E (1.000); 297S (1.000);300R (0.938); 304V (0.953);305K (0.994)
	Null model	-48674.379	p0 = 0.587, p1 = 0.088, p2a = 0.283, p2b = 0.042<br>background: ω0 = 0.083, ω1 = 1.000, ω2a = 0.083, ω2b = 1.000<br>foreground; ω0 = 0.083, ω1 = 1.000, ω2a = 1.000, ω2b = 1.000				
**Lycophytes**	Model A	-48822.348	p0 = 0.814, p1 = 0.128, p2a = 0.050, p2b = 0.008<br>background: ω0 = 0.087, ω1 = 1.000, ω2a = 0.087, ω2b = 1.000	0.592	1	0.442	27L (0.989);31E (0.958); 112A (0.971);179P (0.999); 222D (0.956);300R (0.979)

			foreground: ω0 = 0.087, ω1 = 1.000, ω2a = 1.626, ω2b = 1.626				
	Null model	-48822.644	p0 = 0.801, p1 = 0.127, p2a = 0.063, p2b = 0.010<br>background: ω0 = 0.087, ω1 = 1.000, ω2a = 0.087, ω2b = 1.000<br>foreground: ω0 = 0.087, ω1 = 1.000, ω2a = 1.000, ω2b = 1.000				
**Bryophytes**	Model A	-48829.428	p0 = 0.850, p1 = 0.123, p2a = 0.0235, p2b = 0.003<br>background: ω0 = 0.088, ω1 = 1.000, ω2a = 0.088, ω2b = 1.000<br>foreground: ω0 = 0.088, ω1 = 1.000, ω2a = 1.000, ω2b = 1.000	0	1	1	222D (0.991)
	Null model	-48829.428	p0 = 0.851, p1 = 0.123, p2a = 0.023, p2b = 0.003<br>background: ω0 = 0.088, ω1 = 1.000, ω2a = 0.088, ω2b = 1.000<br>foreground: ω0 = 0.088, ω1 = 1.000, ω2a = 1.000, ω2b = 1.000				

[a] lnL: log-likelihood scores.

[b] -2ΔlnL: twice the log-likelihood difference of the models being compared.

[C] d.f.: degree of freedom.

Table S4. Results of branch-site tests by treating each branch in the phylogeny as the foreground branch.

Foreground branches	Model	lnL[a]	Parameter estimates	-2ΔlnL[b]	d.f.[c]	*p*-value	Positively Selected sites
Ananas comosus (*AncGGP-3*)	Model A	-48817.404	p0 = 0.784, p1 = 0.125, p2a = 0.079, p2b = 0.013<br>Background: ω0 = 0.087, ω1 = 1.000, ω2a = 0.087, ω2b = 1.000<br>Foreground: ω0 = 0.087, ω1 = 1.0000 ω2a =5.443, ω2b = 5.443	8.041	1	0.0046*	1T (0.987); 7Q (0.987); 59A (0.981); 60Q (0.965); 214N (0.992); 245E (0.984); 265V (0.993)；287Y (0.991); 303E (0.992)
	Null model	-48821.424	p0 = 0.717, p1 = 0.115, p2a = 0.145, p2b = 0.023<br>Background: ω0 = 0.086, ω1 = 1.000, ω2a = 0.086, ω2b = 1.000<br>Foreground: ω0 = 0.086, ω1 = 1.000, ω2a =1.000, ω2b = 1.000				
Arabidopsis thaliana (*AtGGP-1*)	Model A	-48830.433	p0 = 0.860, p1 = 0.137, p2a = 0.003, p2b = 0.00046<br>Background: ω0 = 0.087 ω1 = 1.000, ω2a = 0.087, 9ω2b = 1.000<br>Foreground: ω0 = 0.087, ω1 = 1.000, ω2a =43.808, ω2b = 43.808	5.493	1	0.019*	215A (0.995)
	Null model	-48833.180	p0 = 0.808, p1 = 0.129, p2a = 0.055, p2b = 0.009<br>Background: ω0 = 0.087 ω1 = 1.000, ω2a = 0.087, 9ω2b = 1.000<br>Foreground: ω0 = 0.087, ω1 = 1.000,				

			ω2a =1.000, ω2b = 1.000				
Brachypodium_stacei (*BsGGP-1*)	Model A	-48830.372	p0 = 0.861, p1 = 0.137, p2a = 0.002, p2b = 0.00033<br>Background: ω0 = 0.087, ω1 = 1.000, ω2a = 0.087, ω2b = 1.000<br>Foreground: ω0 = 0.087, ω1 = 1.000, ω2a =76.292, ω2b = 76.292	5.983	1	0.014*	187T (0.995)
	Null model	-48833.364	p0 = 0.82, p1 = 0.13, p2a = 0.043, p2b = 0.007<br>Background: ω0 = 0.088, ω1 = 1.000, ω2a = 0.088, ω2b = 1.000<br>Foreground: ω0 = 0.088, ω1 = 1.000, ω2a =1.000, ω2b = 1.000				
Brassica_oleracea (*BoGGP-2*)	Model A	-48810.375	p0 = 0.838, p1 = 0.134, p2a = 0.025, p2b = 0.004<br>Background: ω0 = 0.087, ω1 = 1.000, ω2a = 0.087 ω2b = 1.000<br>Foreground: ω0 = 0.087, ω1 = 1.000, ω2a = 23.625., ω2b = 23.625	26.261	1	0.0000**	1T (0.975); 5I (1.000); 6N (0.999); 7Q (0.999); 15- (1.000)
	Null model	-48823.506	p0 = 0.715, p1 = 0.114, p2a = 0.148, p2b = 0.024<br>Background: ω0 = 0.087, ω1 = 1.000, ω2a = 0.087 ω2b = 1.000<br>Foreground: ω0 = 0.087, ω1 = 1.000, ω2a =1.000, ω2b = 1.000				
Brassica_rapa	Modle A	-48805.884	p0 = 0.816, p1 = 0.13, p2a = 0.047, p2b	20.204	1	0.0000**	1T (0.969); 17G (0.960); 51L (0.969);

(*BrGGP-4*)			= 0.007<br>Background: ω0 = 0.087, ω1 = 1.000, ω2a = 0.087, ω2b = 1.000<br>Foreground: ω0 = 0.087, ω1 = 1.000, ω2a = 45.8, ω2b = 45.8				75R (0.969); 107A (0.965); 207E (0.961); 225P (0.966); 251E (0.967); 281D (0.967); 283A (0.998); 284T (1.000); 285E (0.965); 287Y (1.000); 288A (0.965); 289K (0.995)
	Null model	-48815.986	p0 = 0.334, p1 = 0.053, p2a = 0.528, p2b = 0.084<br>Background: ω0 = 0.087, ω1 = 1.000, ω2a = 0.087, ω2b = 1.000<br>Foreground: ω0 = 0.087, ω1 = 1.000, ω2a =1.000, ω2b = 1.000				
Chlamydomonas_reinhardtii (*ChrGGP*)	Model A	-48800.092	p0 = 0.728, p1 = 0.112, p2a = 0.139, p2b = 0.021<br>Background: ω0 = 0.087, ω1 = 1.000, ω2a = 0.087 ω2b = 1.000<br>Foreground: ω0 = 0.087, ω1 = 1.000, ω2a = 27.048., ω2b = 27.048	36.411	1	0.0000*	11- (0.999); 12- (0.999); 22L (0.960); 47E (0.956); 87K (1.000); 148V (0.998); 151P (0.999); 152Y (0.999); 159S (1.000); 203T (0.951); 230I (1.000); 265V (0.953); 284T (0.996); 289K (0.994); 294E (1.000); 297S (0.998)
	Null model	-48818.298	p0 = 0.723, p1 = 0.112, p2a = 0.144, p2b = 0.022<br>Background: ω0 = 0.087, ω1 = 1.000, ω2a = 0.087, ω2b = 1.000<br>Foreground: ω0 = 0.087, ω1 = 1.000, ω2a =1.000, ω2b = 1.000				
Coccomyxa_subellipsoidea (*CosGGP*)	Model A	-48808.925	p0 = 0.7, p1 = 0.114, p2a = 0.161 p2b = 0.026<br>Background: ω0 = 0.087, ω1 = 1.000,	17.84	1	0.0000**	2V (0.997); 5I (0.992); 6N (0.978); 7Q (1.000); 47E (0.958); 94D (0.999); 131P (0.997); 135C (0.999); 140L (0.995); 190G

			ω2a = 0.087 ω2b = 1.000<br>Foreground: ω0 = 0.087, ω1 = 1.000, ω2a =7.241, ω2b = 7.241				(0.975); 220Q (1.000); 241Q (0.990); 245E (0.985); 246R (0.997); 251E (0.984); 260Q (0.985); 287Y (0.993); 296L (0.996); 300R (0.983)
	Null model	-48817.845	p0 = 0.713, p1 = 0.115 p2a = 0.148 p2b = 0.024<br>Background: ω0 = 0.086, ω1 = 1.000, ω2a = 0.086, ω2b = 1.000<br>Foreground: ω0 = 0.086, ω1 = 1.000, ω2a =1.000, ω2b = 1.000				
Dunaliella_salina (*DsGGP*)	Model A	-48808.785	p0 = 0.724, p1 = 0.114, p2a = 0.14, p2b = 0.022<br>Background: ω0 = 0.087, ω1 = 1.000, ω2a = 0.087, ω2b = 1.000<br>Foreground: ω0 = 0.087, ω1 = 1.000, ω2a = 21.654, ω2b = 21.654	29.163	1	0.0000**	4S (0.999); 6N (0.990); 75R (0.996); 80L (0.990); 84D (0.998); 94D (0.953); 134I(0.989); 215A(0.999); 217I(1.000); 226F(0.953); 240I(0.999); 242C(0.968); 246R(0.999); 283A(0.995); 284T(0.992); 297S(0.996)
	Null model	-48823.367	p0 = 0.747, p1 = 0.118, p2a = 0.116, p2b = 0.018<br>Background: ω0 = 0.087, ω1 = 1.000, ω2a = 0.087, ω2b = 1.000<br>Foreground: ω0 = 0.087, ω1 = 1.000, ω2a =1.000, ω2b = 1.000				
Eucalyptus grandis (*EgGGP-5*)	Model A	-48818.108	p0 = 0.832, p1 = 0.133, p2a = 0.031, p2b = 0.005<br>Background: ω0 = 0.087, ω1 = 1.000, ω2a = 0.087, ω2b = 1.000	12.113	1	0.0005*	37G (0.968); 62N (1.000); 63E (1.000); 125Q (0.966); 135C (0.965); 216C (0.968); 264A (0.967); 284T (0.969)

			Foreground: ω0 = 0.087, ω1 = 1.000, ω2a = 59.268, ω2b = 59.268				
	Null model	-48824.164	p0 = 0.379, p1 = 0.06, p2a = 0.484, p2b = 0.077<br>Background: ω0 = 0.087, ω1 = 1.000, ω2a = 0.087, ω2b = 1.000<br>Foreground: ω0 = 0.087, ω1 = 1.000, ω2a =1.000, ω2b = 1.000				
Glycine max (*GmGGP-4*)	Model A	-48824.508	p0 = 0.858, p1 = 0.137, p2a = 0.005, p2b = 0.00079<br>Background: ω0 = 0.087, ω1 = 1.000, ω2a = 0.087, ω2b = 1.000<br>Foreground: ω0 = 0.087, ω1 = 1.000, ω2a = 73.352, ω2b = 73.352	8.194	1	0.004*	304V (0.998); 305K (0.997)
	Null model	-48828.605	p0 = 0.71, p1 = 0.113, p2a = 0.152, p2b = 0.024<br>Background: ω0 = 0.087, ω1 = 1.000, ω2a = 0.087 ω2b = 1.000<br>Foreground: ω0 = 0.087, ω1 = 1.000, ω2a =1.000, ω2b = 1.000				
Marchantia polymorpha (*MpGGP*)	Model A	-48827.668	p0 = 0.838, p1 = 0.133, p2a = 0.025, p2b = 0.004<br>Background: ω0 = 0.087, ω1 = 1.000, ω2a = 0.087, ω2b = 1.000<br>Foreground: ω0 = 0.087, ω1 = 1.000, ω2a = 7.806, ω2b = 7.806	8.527	1	0.003*	127K (0.982); 128S (0.955); 203T (0.970)

	Null model	-48831.931	p0 = 0.834, p1 = 0.133, p2a = 0.029, p2b = 0.005<br>Background: ω0 = 0.087, ω1 = 1.000, ω2a = 0.087 ω2b = 1.000<br>Foreground: ω0 = 0.087, ω1 = 1.000, ω2a =1.000, ω2b = 1.000				
Micromonas_pusilla (*MpGGP*)	Model A	-48803.535	p0 = 0.728, p1 = 0.112, p2a = 0.138, p2b = 0.021<br>Background: ω0 = 0.087, ω1 = 1.000, ω2a = 0.087, ω2b = 1.000<br>Foreground: ω0 = 0.087, ω1 = 1.000, ω2a = 13.253, ω2b = 13.253	26.221	1	0.0000**	15- (0.999); 19S (0.993); 75R (0.998); 123V (1.000); 214N (0.977); 215A (0.977); 241Q (0.999); 242C (1.000); 284T (0.999); 289K (0.989)
	Null model	-48816.645	p0 = 0.675, p1 = 0.105, p2a = 0.19, p2b = 0.03<br>Background: ω0 = 0.086, ω1 = 1.000, ω2a = 0.086 ω2b = 1.000<br>Foreground: ω0 = 0.086, ω1 = 1.000, ω2a =1.000, ω2b = 1.000				
Micromonas sp RCC299 (*MsGGP*)	Model A	-48804.458	p0 = 0.793, p1 = 0.126, p2a = 0.07, p2b = 0.011<br>Background: ω0 = 0.087, ω1 = 1.000, ω2a = 0.087, ω2b = 1.000<br>Foreground: ω0 = 0.087, ω1 = 1.000, ω2a = 16.994, ω2b = 16.994	34.717	1	0.0000**	4S (0.999); 12- (0.999); 22L (0.986); 47E (0.967); 80L (0.999); 101A (0.965); 107A (0.998); 138T (0.998); 215A (0.998); 287Y (0.998); 289K (0.964); 297S (0.999)
	Null model	-48821.817	p0 = 0.755, p1 = 0.12, p2a = 0.108, p2b = 0.017				

			Background: ω0 = 0.087, ω1 = 1.000, ω2a = 0.087 ω2b = 1.000 Foreground: ω0 = 0.087, ω1 = 1.000, ω2a =1.000, ω2b = 1.000				
Mimulus guttatus (*MgGGP-2*)	Mode A	-48831.088	p0 = 0.859, p1 = 0.137, p2a = 0.004, p2b = 0.001 Background: ω0 = 0.087, ω1 = 1.000, ω2a = 0.087, ω2b = 1.000 Foreground: ω0 = 0.087, ω1 = 1.000, ω2a = 17.229, ω2b = 17.229	4.794	1	0.029*	129Q (0.953)
	Null model	-48833.485	p0 = 0.851, p1 = 0.136, p2a = 0.012, p2b = 0.002 Background: ω0 = 0.087, ω1 = 1.000, ω2a = 0.087 ω2b = 1.000 Foreground: ω0 = 0.087, ω1 = 1.000, ω2a =1.000, ω2b = 1.000				
Musa acuminata (*MaGGP-1*)	ModelA	-48831.56	p0 = 0.856, p1 = 0.136, p2a = 0.007, p2b = 0.001 Background: ω0 = 0.087, ω1 = 1.000, ω2a = 0.087, ω2b = 1.000 Foreground: ω0 = 0.087, ω1 = 1.000, ω2a = 9.465, ω2b = 9.465	4.01	1	0.045*	29Q (0.983); 190G (0.984)
	Null model	-48833.565	p0 = 0.842, p1 = 0.134, p2a = 0.021, p2b = 0.003 Background: ω0 = 0.087, ω1 = 1.000, ω2a = 0.087 ω2b = 1.000				

			Foreground: ω0 = 0.087, ω1 = 1.000, ω2a =1.000, ω2b = 1.000				
Ostreococcus lucimarinus (*OlGGP*)	Model A	-48801.958	p0 = 0.763, p1 = 0.115, p2a = 0.107, p2b = 0.016 Background: ω0 = 0.087, ω1 = 1.000, ω2a = 0.087, ω2b = 1.000 Foreground: ω0 = 0.087, ω1 = 1.000, ω2a = 9.474, ω2b = 9.474	30.123	1	0.0000**	46C (0.997); 47E (0.953); 52P (0.996); 56G (0.992); 63E (0.999); 95Q (0.982); 129Q (0.998); 191C (0.997); 215A (0.955); 216C (0.998); 245E (0.999); 250G (0.990); 277R (0.972); 289K (0.997); 290K (0.980)
	Null model	-48817.019	p0 = 0.744, p1 = 0.117, p2a = 0.12, p2b = 0.019 Background: ω0 = 0.086, ω1 = 1.000, ω2a = 0.086, ω2b = 1.000 Foreground: ω0 = 0.086, ω1 = 1.000, ω2a =1.000, ω2b = 1.000				
Panicum virgatum (*PvGGP-2*)	Model A	-48791.388	p0 = 0.849, p1 = 0.136, p2a = 0.013, p2b = 0.002 Background: ω0 = 0.087, ω1 = 1.000, ω2a = 0.087, ω2b = 1.000 Foreground: ω0 = 0.087, ω1 = 1.000, ω2a = 87.418, ω2b = 87.418	55.025	1	0.0000**	5I (0.999); 6N (0.999); 7Q (1.000); 8- (1.000); 9- (1.000); 12- (1.000)
	Null model	-48818.900	p0 = 0.709, p1 = 0.113, p2a = 0.154, p2b = 0.025 Background: ω0 = 0.087, ω1 = 1.000, ω2a = 0.087, ω2b = 1.000 Foreground: ω0 = 0.087, ω1 = 1.000, ω2a =1.000, ω2b = 1.000				

Picea abies (*PaGGP-3*)	Model A	-48799.663	p0 = 0.855, p1 = 0.136, p2a = 0.008, p2b = 0.001<br>Background: ω0 = 0.087, ω1 = 1.000, ω2a = 0.087, ω2b = 1.000<br>Foreground: ω0 = 0.087, ω1 = 1.000, ω2a = 969.357, ω2b = 969.357	46.735	1	0.0000**	1T (1.000); 2V (0.993); 4S (1.000); 5I (0.999); 6N (1.000); 7Q (0.993)
	Null model	-48823.030	p0 = 0.476, p1 = 0.076, p2a = 0.387, p2b = 0.062<br>Background: ω0 = 0.087, ω1 = 1.000, ω2a = 0.087, ω2b = 1.000<br>Foreground: ω0 = 0.087, ω1 = 1.000, ω2a =1.000, ω2b = 1.000				
Selaginella moellendorffii (*SmGGP-2*)	Model A	-48824.521	p0 = 0.811, p1 = 0.130, p2a = 0.051, p2b = 0.008<br>Background: ω0 = 0.087, ω1 = 1.000, ω2a = 0.087, ω2b = 1.000<br>Foreground: ω0 = 0.087, ω1 = 1.000, ω2a = 4.037, ω2b = 4.037	5.590	1	0.018*	109N (0.964); 112A (0.969); 113I (0.984); 151P (0.978); 179P (0.966); 297S (0.992)
	Null model	-48827.316	p0 = 0.782, p1 = 0.125, p2a = 0.08, p2b = 0.013<br>Background: ω0 = 0.087, ω1 = 1.000, ω2a = 0.087, ω2b = 1.000<br>Foreground: ω0 = 0.087, ω1 = 1.000, ω2a =1.000, ω2b = 1.000				
Selaginella moellendorffii (*SmGGP-3*)	Model A	-48814.674	p0 = 0.758, p1 = 0.116, p2a = 0.109, p2b = 0.017	10.297	1	0.001*	8- (0.984); 9- (0.971); 34M (0.956); 82P (0.991); 112A (0.955); 138T (0.965);

			Background: ω0 = 0.087, ω1 = 1.000, ω2a = 0.087, ω2b = 1.000 Foreground: ω0 = 0.087, ω1 = 1.000, ω2a = 3.798, ω2b = 3.798				150N (0.953); 190G (0.988); 217I (0.995); 222D (0.974); 231A (0.980); 245E (0.970); 250G (0.983); 253D (0.975); 290K (0.990); 299D (0.989); 300R (0.985)
	Null model	-48819.823	p0 = 0.741, p1 = 0.116, p2a = 0.123, p2b = 0.019 Background: ω0 = 0.087, ω1 = 1.000, ω2a = 0.087, ω2b = 1.000 Foreground: ω0 = 0.087, ω1 = 1.000, ω2a =1.000, ω2b = 1.000				
Volvox carteri (*VcGGP*)	Model A	-48804.773	p0 = 0.755, p1 = 0.118, p2a = 0.109, p2b = 0.017 Background: ω0 = 0.087, ω1 = 1.000, ω2a = 0.087, ω2b = 1.000 Foreground: ω0 = 0.087, ω1 = 1.000, ω2a = 16.335, ω2b = 16.335	32.725	1	0.0000**	8- (0.999); 9- (0.961); 18A (0.965); 22L (0.992); 23Y (0.999); 47E (0.966); 148V (0.990); 184R (0.999); 190G (0.972); 202R (0.998); 251E (0.998); 303E (1.000)
	Null model	-48821.135	p0 = 0.745, p1 = 0.118, p2a = 0.119, p2b = 0.019 Background: ω0 = 0.087, ω1 = 1.000, ω2a = 0.087, ω2b = 1.000 Foreground: ω0 = 0.087, ω1 = 1.000, ω2a =1.000, ω2b = 1.000				
Zea mays (*ZmGGP-1*)	Model A	-48818.760	p0 = 0.846, p1 = 0.135, p2a = 0.016, p2b = 0.003 Background: ω0 = 0.087, ω1 = 1.000, ω2a = 0.087, ω2b = 1.000	21.888	1	0.0000**	25D (0.996); 26S (0.996); 27L (0.996); 28S (1.000); 29Q (0.996)

			Foreground: ω0 = 0.087, ω1 = 1.000, ω2a = 999.000, ω2b = 999.000				
	Null model	-48829.704	p0 = 0.449, p1 = 0.072, p2a = 0.414, p2b = 0.066<br>Background: ω0 = 0.087, ω1 = 1.000, ω2a = 0.087, ω2b = 1.000<br>Foreground: ω0 = 0.087, ω1 = 1.000, ω2a =1.000, ω2b = 1.000				

[a] lnL: log-likelihood scores.

[b] -2ΔlnL: twice the log-likelihood difference of the models being compared.

[C] d.f.: degree of freedom.

* $p<0.05$; ** $p<0.0003$ (Bonferroni correction for a significance level of 0.05 over 149 independent tests).

Text S1. Alignment of plant GGP protein sequences and the histidine triad (HIT) motif is marked using box.

```
                ....|....| ....|....| ....|....| ....|....| ....|....| ....|....| ....|....| ....|....| ....|....| ....|....| ....|....| ....|....|
                         10         20         30         40         50         60         70         80         90        100        110        120
AcGGP           ---------- ---------- ---------- ---------- ---------- ---------- ---------- ----MLK--- ---------- ---------- ---------- ----------
AcGGP-R-1       ---------- ---------- ---------- ---------- ---------- ---------- ---------- ----MLK--- ---------- ---------- ---------- ----------
AcGGP-R-2       M--------- ---------- ---------- ---------- ---------- ---------- -------LNT ATQ-MLT--- ---------- ---------- ---------- ----------
AdGGP           ---------- ---------- ---------- ---------- ---------- ---------- ---------- ----MLK--- ---------- ---------- ---------- ----------
AeGGP           ---------- ---------- ---------- ---------- ---------- ---------- ---------- ----MLK--- ---------- ---------- ---------- ----------
ArGGP           ---------- ---------- ---------- ---------- ---------- ---------- ---------- ----MLK--- ---------- ---------- ---------- ----------
AmhGGP-1        ---------- ---------- ---------- ---------- ---------- ---------- ---------- ----MLR--- ---------- ---------- ---------- ----------
AmhGGP-2        ---------- ---------- ---------- ---------- ---------- ---------- -------MGI LEK-SLT--- ---------- ---------- ---------- ----------
AncGGP-3        ---------- ---------- ---------- ---------- ---------- ---------- ---------- ----MLSEA- ---------- ---------- ---------- ----------
AncGGP-1        ---------- ---------- ---------- ---------- ---------- ---------- ---------- ----MLT--- ---------- ---------- ---------- ----------
AncGGP-2        ---------- ---------- ---------- ---------- ---------- ---------- ---------- ----MLT--- ---------- ---------- ---------- ----------
AqcGGP          ---------- ---------- ---------- ---------- ---------- ---------- ---------- ----MLR--- ---------- ---------- ---------- ----------
AhGGP-1         ---------- ---------- ---------- ---------- ---------- ---------- ---------- ----MLK--- ---------- ---------- ---------- ----------
AhGGP-2         ---------- ---------- ---------- ---------- ---------- ---------- ---------- --M-LLK--- ---------- ---------- ---------- ----------
AlGGP-1         ---------- ---------- ---------- ---------- ---------- ---------- ---------- ----MLK--- ---------- ---------- ---------- ----------
AlGGP-2         ---------- ---------- ---------- ---------- ---------- ---------- ---------- --M-LLK--- ---------- ---------- ---------- ----------
AtGGP-1         ---------- ---------- ---------- ---------- ---------- ---------- ---------- ----MLK--- ---------- ---------- ---------- ----------
AtGGP-2         ---------- ---------- ---------- ---------- ---------- ---------- ---------- --M-LLK--- ---------- ---------- ---------- ----------
BosGGP-1        ---------- ---------- ---------- ---------- ---------- ---------- ---------- ----MLK--- ---------- ---------- ---------- ----------
BosGGP-2        ---------- ---------- ---------- ---------- ---------- ---------- ---------- --M-LLK--- ---------- ---------- ---------- ----------
BdGGP-1         ---------- ---------- ---------- ---------- ---------- ---------- ---------- MEM-KLT--- ---------- ---------- ---------- ----------
BdGGP-2         ---------- ---------- ---------- ---------- ---------- ---------- ---------- MEV-KLT--- ---------- ---------- ---------- ----------
BdGGP-3         ---------- ---------- ---------- ---------- ---------- ---------- ---------- ---------- ---------- ---------- ---------- ----------
BsGGP-1         ---------- ---------- ---------- ---------- ---------- ---------- ---------- MEM-KLT--- ---------- ---------- ---------- ----------
BsGGP-2         ---------- ---------- ---------- ---------- ---------- ---------- ---------- MEV-KLT--- ---------- ---------- ---------- ----------
BsGGP-3         ---------- ---------- ---------- ---------- ---------- ---------- ---------- ---------- ---------- ---------- ---------- ----------
BoGGP-1         ---------- ---------- ---------- ---------- ---------- ---------- ---------- ----MLK--- ---------- ---------- ---------- ----------
BoGGP-2         ---------- ---------- ---------- ---------- ---------- ---------- ---------- ----MLK--- ---------- ---------- ---------- ----------
BoGGP-3         ---------- ---------- ---------- ---------- ---------- ---------- ---------- --M-LLK--- ---------- ---------- ---------- ----------
BrGGP-1         ---------- ---------- ---------- ---------- ---------- ---------- ---------- ----MLK--- ---------- ---------- ---------- ----------
BrGGP-2         ---------- ---------- ---------- ---------- ---------- ---------- ---------- ----MLK--- ---------- ---------- ---------- ----------
BrGGP-3         ---------- ---------- ---------- ---------- ---------- ---------- ---------- ----MLK--- ---------- ---------- ---------- ----------
BrGGP-4         ---------- ---------- ---------- ---------- ---------- ---------- ---------- --M-LLK--- ---------- ---------- ---------- ----------
CgGGP-1         ---------- ---------- ---------- ---------- ---------- ---------- ---------- ----MLK--- ---------- ---------- ---------- ----------
CgGGP-2         ---------- ---------- ---------- ---------- ---------- ---------- ---------- --M-LLK--- ---------- ---------- ---------- ----------
CrGGP-1         ---------- ---------- ---------- ---------- ---------- ---------- ---------- ----MLK--- ---------- ---------- ---------- ----------
CrGGP-2         ---------- ---------- ---------- ---------- ---------- ---------- ---------- --M-LLK--- ---------- ---------- ---------- ----------
ChrGGP          ---------- ---------- ---------- ---------- ---------- ---------- ---------- --MDSLQ-A- L--------- ---------- ---------- ----------
CcGGP-1         ---------- ---------- ---------- ---------- ---------- ---------- ---------- --M-MLR--- ---------- ---------- ---------- ----------
CcGGP-2         ---------- ---------- ---------- ---------- ---------- ---------- ---------- ----MLT--- ---------- ---------- ---------- ----------
CsGGP-1         ---------- ---------- ---------- ---------- ---------- ---------- ---------- --M-MLR--- ---------- ---------- ---------- ----------
CsGGP-2         ---------- ---------- ---------- ---------- ---------- ---------- ---------- ----MLT--- ---------- ---------- ---------- ----------
CosGGP          ---------- ---------- ---------- ---------- ---------- ---------- ---------- ----MYH--- ---------- ---------- ---------- ----------
CusGGP          ---------- ---------- ---------- ---------- ---------- ---------- ---------- ----MLR--- ---------- ---------- ---------- ----------
DcGGP-1         ---------- ---------- ---------- ---------- ---------- ---------- ---------- --MEMLR--- ---------- ---------- ---------- ----------
DcGGP-2         ---------- ---------- ---------- ---------- ---------- ---------- ---------- ----MLR--- ---------- ---------- ---------- ----------
DsGGP           M--------- ---------- ---------L QQVTQQQQQL QQQQQQQQQL DGNVCNSS-- -------MSR SNSNHLT--- ---------- ---------- ---------- ----------
EgGGP-1         ---------- ---------- ---------- ---------- ---------- ---------- ---------- --M-MLK--- ---------- ---------- ---------- ----------
EgGGP-2         ---------- ---------- ---------- ---------- ---------- ---------- ---------- --M-MLK--- ---------- ---------- ---------- ----------
EgGGP-3         ---------- ---------- ---------- ---------- ---------- ---------- ---------- ----MLT--- ---------- ---------- ---------- ----------
EgGGP-4         ---------- ---------- ---------- ---------- ---------- ---------- ---------- ----MLT--- ---------- ---------- ---------- ----------
EgGGP-5         ---------- ---------- ---------- ---------- ---------- ---------- ---------- ----MLT--- ---------- ---------- ---------- ----------
EsGGP-1         ---------- ---------- ---------- ---------- ---------- ---------- ---------- ----MLS--- ---------- ---------- ---------- ----------
EsGGP-2         ---------- ---------- ---------- ---------- ---------- ---------- ---------- --M-LLK--- ---------- ---------- ---------- ----------
FvGGP-1         ---------- ---------- ---------- ---------- ---------- ---------- ---------- --M-MLK--- ---------- ---------- ---------- ----------
FvGGP-2         ---------- ---------- ---------- ---------- ---------- ---------- ---------- --M-MLK--- ---------- ---------- ---------- ----------
FvGGP-3         ---------- ---------- ---------- ---------- ---------- ---------- ---------- --M-MMK--- ---------- ---------- ---------- ----------
GmGGP-1         ---------- ---------- ---------- ---------- ---------- ---------- ---------- --M-MLK--- ---------- ---------- ---------- ----------
GmGGP-2         ---------- ---------- ---------- ---------- ---------- ---------- ---------- --M-MLR--- ---------- ---------- ---------- ----------
GmGGP-3         ---------- ---------- ---------- ---------- ---------- ---------- ---------- ----MLS--- ---------- ---------- ---------- ----------
GmGGP-4         ---------- ---------- ---------- ---------- ---------- ---------- ---------- ----MLS--- ---------- ---------- ---------- ----------
GnmGGP          ---------- ---------- ---------- ---------- ---------- ---------- ---------- ----MLT--- ---------- ---------- ---------- ----------
GrGGP-1         ---------- ---------- ---------- ---------- ---------- ---------- ---------- --M-MLR--- ---------- ---------- ---------- ----------
GrGGP-2         ---------- ---------- ---------- ---------- ---------- ---------- ---------- --M-MLR--- ---------- ---------- ---------- ----------
GrGGP-3         ---------- ---------- ---------- ---------- ---------- ---------- ---------- -MM-QLS--- ---------- ---------- ---------- ----------
KfGGP-1         ---------- ---------- ---------- ---------- ---------- ---------- -------MGE STM-MLK--- ---------- ---------- ---------- ----------
KfGGP-2         ---------- ---------- ---------- ---------- ---------- ---------- -------MGE STM-MLK--- ---------- ---------- ---------- ----------
KlGGP-1         ---------- ---------- ---------- ---------- ---------- ---------- -------MGE STM-MLK--- ---------- ---------- ---------- ----------
KlGGP-2         ---------- ---------- ---------- ---------- ---------- ---------- -------MGE STM-MLK--- ---------- ---------- ---------- ----------
KlGGP-3         ---------- ---------- ---------- ---------- ---------- ---------- -------MGE STM-MLK--- ---------- ---------- ---------- ----------
LuGGP           MLVVTLGKVG CEGGRRVVDI TAIHGVSRPL LHVRRKEVIV IQSNPSAHGG RGALPSEGGS PSDLLFLAGG DKM-MLR--- ---------- ---------- ---------- ----------
MdGGP           ---------- ---------- ---------- ---------- ---------- ---------- ---------- --M-MLR--- ---------- ---------- ---------- ----------
MeGGP-1         M--------- ---------- ---------- ---------- ---------- ---------- ---------S DNF-MLR--- ---------- ---------- ---------- ----------
MeGGP-2         M--------- ---------- ---------- ---------- ---------- ---------- ---------S DNL-MLR--- ---------- ---------- ---------- ----------
MeGGP-3         ---------- ---------- ---------- ---------- ---------- ---------- ---------- --M-LLT--- ---------- ---------- ---------- ----------
MpGGP           ---------- ---------- ---------- ---------- ---------- ---------- ---------- ----MLT--- ---------- ---------- ---------- ----------
MtGGP-1         ---------- ---------- ---------- ---------- ---------- ---------- ---------- --M-MLK--- ---------- ---------- ---------- ----------
MtGGP-2         ---------- ---------- ---------- ---------- ---------- ---------- ---------- ----MLS--- ---------- ---------- ---------- ----------
MpGGP           ---------- ---------- ---------- ---------- ---------- ---------- ---------- ----MAT--- ---------- ---------- ---------- ----------
MsGGP           ---------- ---------- ---------- ---------- ---------- ---------- ---------- --M-SLT--- ---------- ---------- ---------- ----------
MgGGP-1         ---------- ---------- ---------- ---------- ---------- ---------- ---------- --M-MLK--- ---------- ---------- ---------- ----------
MgGGP-2         ---------- ---------- ---------- ---------- ---------- ---------- ---------- --M-MLK--- ---------- ---------- ---------- ----------
MaGGP-1         ---------- ---------- ---------- ---------- ---------- ---------- ---------- ----MLT--- ---------- ---------- ---------- ----------
MaGGP-2         ---------- ---------- ---------- ---------- ---------- ---------- ---------- ----MLS--- ---------- ---------- ---------- ----------
MaGGP-3         ---------- ---------- ---------- ---------- ---------- ---------- ---------- ----MLT--- ---------- ---------- ---------- ----------
MaGGP-4         ---------- ---------- ---------- ---------- ---------- ---------- ---------- ---------- ---------- ---------- ---------- ----------
OtGGP           ---------- ---------- ---------- ---------- ---------- ---------- ---------- MEM-KLT--- ---------- ---------- ---------- ----------
OsGGP-1         ---------- ---------- ---------- ---------- ---------- ---------- ---------- MEM-KLT--- ---------- ---------- ---------- ----------
OsGGP-2         ---------- ---------- ---------- ---------- ---------- ---------- ---------- ----MPISPL ---------- ---------- ---------- ----------
OlGGP           ---------- ---------- ---------- ---------- ---------- ---------- ---------- ---------- ---------- ---------- ---------- ----------
PhGGP-1         ---------- ---------- ---------- ---------- ---------- ---------- ---------- MEM-KLT--- ---------- ---------- ---------- ----------
PhGGP-2         ---------- ---------- ---------- ---------- ---------- ---------- ---------- ---------- ---------- ---------- ---------- ----------
PvGGP-1         ---------- ---------- ---------- ---------- ---------- ---------- ---------- MEM-KLT--- ---------- ---------- ---------- ----------
PvGGP-2         ---------- ---------- ---------- ---------- ---------- ---------- ---------- MATCSLTRLT VC-------- ---------- ---------- ----------
PvGGP-3         ---------- ---------- ---------- ---------- ---------- ---------- ---------- ---------- ---------- ---------- ---------- ----------
PhvGGP-1        ---------- ---------- ---------- ---------- ---------- ---------- ---------- --M-MLK--- ---------- ---------- ---------- ----------
PhvGGP-2        ---------- ---------- ---------- ---------- ---------- ---------- ---------- ----MLS--- ---------- ---------- ---------- ----------
PpGGP-1         ---------- ---------- ---------- ---------- ---------- ---------- ---------- ----MLT--- ---------- ---------- ---------- ----------
PpGGP-2         ---------- ---------- ---------- ---------- ---------- ---------- ---------- ----MLT--- ---------- ---------- ---------- ----------
PpGGP-3         ---------- ---------- ---------- ---------- ---------- ---------- ---------- ----MLT--- ---------- ---------- ---------- ----------
PaGGP-1         ---------- ---------- ---------- ---------- ---------- ---------- ---------- ----MLT--- ---------- ---------- ---------- ----------
PaGGP-2         ---------- ---------- ---------- ---------- ---------- ---------- ---------- ----MVTKQD HVDKTQTGSL SLASPFGISA FSAFPHFRPH DRRRILRSRN
PaGGP-3         ---------- ---------- ---------- ---------- ---------- ---------- ---------- --MPLGR--- ---------- ---------- ---------- ----------
PsGGP-1         ---------- ---------- ---------- ---------- ---------- ---------- ---------- ----MLT--- ---------- ---------- ---------- ----------
PsGGP-2         ---------- ---------- ---------- ---------- ---------- ---------- ---------- ----MLT--- ---------- ---------- ---------- ----------
PsGGP-3         ---------- ---------- ---------- ---------- ---------- ---------- ---------- ----MLT--- ---------- ---------- ---------- ----------
PtGGP-1         ---------- ---------- ---------- ---------- ---------- ---------- ---------- ----MLT--- ---------- ---------- ---------- ----------
PtGGP-2         ---------- ---------- ---------- ---------- ---------- ---------- ---------- ----MLT--- ---------- ---------- ---------- ----------
PtGGP-3         ---------- ---------- ---------- ---------- ---------- ---------- ---------- ----MLT--- ---------- ---------- ---------- ----------
PtGGP-4         ---------- ---------- ---------- ---------- ---------- ---------- ---------- ----MLT--- ---------- ---------- ---------- ----------
PtGGP-5         ---------- ---------- ---------- ---------- ---------- ---------- ---------- ----MLT--- ---------- ---------- ---------- ----------
PotGGP-1        ---------- ---------- ---------- ---------- ---------- ---------- ---------- --M-MLR--- ---------- ---------- ---------- ----------
PotGGP-2        ---------- ---------- ---------- ---------- ---------- ---------- ---------- --M-MLR--- ---------- ---------- ---------- ----------
```

```
PotGGP-3     ---------- ---------- ---------- ---------- ---------- ---------- ---------- --M-VLT--- ---------- ---------- ---------- ----------
PrpGGP       ---------- ---------- ---------- ---------- ---------- ---------- ---------- --M-MLR--- ---------- ---------- ---------- ----------
RcGGP-1      M--------- ---------- ---------- ---------- ---------- ---------- ---------S GKF-MLS--- ---------- ---------- ---------- ----------
RcGGP-2      ---------- ---------- ---------- ---------- ---------- ---------- ---------- ---------- ---------- ---------- ---------- ----------
RcGGP-3      ---------- ---------- ---------- ---------- ---------- ---------- ---------- ----MLT--- ---------- ---------- ---------- ----------
SpGGP-1      ---------- ---------- ---------- ---------- ---------- ---------- ---------- --M-MLR--- ---------- ---------- ---------- ----------
SpGGP-2      ---------- ---------- ---------- ---------- ---------- ---------- ---------- --M-MLK--- ---------- ---------- ---------- ----------
SpGGP-3      ---------- ---------- ---------- ---------- ---------- ---------- ---------- ----MLT--- ---------- ---------- ---------- ----------
SmGGP-1      ---------- ---------- ---------- ---------- ---------- ---------- ---------- ----MLT--- ---------- ---------- ---------- ----------
SmGGP-2      ---------- ---------- ---------- ---------- ---------- ---------- ---------- ---------- ---------- ---------- ---------- ----------
SmGGP-3      ---------- ---------- ---------- ---------- ---------- ---------- ---------- ----MLA--- ---------- ---------- ---------- ----------
SiGGP-1      ---------- ---------- ---------- ---------- ---------- ---------- ---------- MEM-KLT--- ---------- ---------- ---------- ----------
SiGGP-2      ---------- ---------- ---------- ---------- ---------- ---------- ---------- ----MTVDTL ---------- ---------- ---------- ----------
SvGGP-1      ---------- ---------- ---------- ---------- ---------- ---------- ---------- MEM-KLT--- ---------- ---------- ---------- ----------
SvGGP-2      ---------- ---------- ---------- ---------- ---------- ---------- ---------- ----MTVDTL ---------- ---------- ---------- ----------
SlGGP-1      ---------- ---------- ---------- ---------- ---------- ---------- ---------- --M-MLK--- ---------- ---------- ---------- ----------
SlGGP-2      ---------- ---------- ---------- ---------- ---------- ---------- ---------- ----MLT--- ---------- ---------- ---------- ----------
StGGP-1      ---------- ---------- ---------- ---------- ---------- ---------- ---------- --M-MLK--- ---------- ---------- ---------- ----------
StGGP-2      ---------- ---------- ---------- ---------- ---------- ---------- ---------- ----MLT--- ---------- ---------- ---------- ----------
SbGGP-1      ---------- ---------- ---------- ---------- ---------- ---------- ---------- MEM-KLT--- ---------- ---------- ---------- ----------
SbGGP-2      ---------- ---------- ---------- ---------- ---------- ---------- ---------- ----MSVTAL ---------- ---------- ---------- ----------
SfGGP-1      ---------- ---------- ---------- ---------- ---------- ---------- ---------- ----MLT--- ---------- ---------- ---------- ----------
SfGGP-2      ---------- ---------- ---------- ---------- ---------- ---------- ---------- ----MLT--- ---------- ---------- ---------- ----------
SppGGP-1     ---------- ---------- ---------- ---------- ---------- ---------- ---------- ----MLT--- ---------- ---------- ---------- ----------
SppGGP-2     ---------- ---------- ---------- ---------- ---------- ---------- ---------- ----MLT--- ---------- ---------- ---------- ----------
TcGGP-1      ---------- ---------- ---------- ---------- ---------- ---------- ---------- --M-MLR--- ---------- ---------- ---------- ----------
TcGGP-2      ---------- ---------- ---------- ---------- ---------- ---------- ---------- ----MLT--- ---------- ---------- ---------- ----------
TpGGP        ---------- ---------- ---------- ---------- ---------- ---------- ---------- --M-MLK--- ---------- ---------- ---------- ----------
VvGGP-1      ---------- ---------- ---------- ---------- ---------- ---------- ---------- --MNTLR--- ---------- ---------- ---------- ----------
VvGGP-2      ---------- ---------- ---------- ---------- ---------- ---------- ---------- ----MLT--- ---------- ---------- ---------- ----------
VcGGP        ---------- ---------- ---------- ---------- ---------- ---------- ---------- --MEMICE-Q ---------- ---------- ---------- ----------
ZmGGP-E-1    ---------- ---------- ---------- ---------- ---------- ---------- ---------- MEM-KLT--- ---------- ---------- ---------- ----------
ZmGGP-E-2    ---------- ---------- ---------- ---------- ---------- ---------- ---------- ----MTRRSD AR-------- ---------- ---------- ----------
ZmGGP-1      ---------- ---------- ---------- ---------- ---------- ---------- ---------- MEM-KLT--- ---------- ---------- ---------- ----------
ZmGGP-2      ---------- ---------- ---------- ---------- ---------- ---------- ---------- ----MTRRSD AR-------- ---------- ---------- ----------
ZomGGP       ---------- ---------- ---------- ---------- ---------- ---------- ---------M AEM-KLT--- ---------- ---------- ---------- ----------

             ....|....| ....|....| ....|....| ....|....| ....|....| ....|....| ....|....| ....|....| ....|....| ....|....| ....|....| ....|....|
                    130        140        150        160        170        180        190        200        210        220        230        240
AcGGP        ---------- ---------- ---------- ---------- ---------- -----I---- ---KR----- ---------- ---------- ---------- ---------- -----V---P
AcGGP-R-1    ---------- ---------- ---------- ---------- ---------- -----I---- ---KR----- ---------- ---------- ---------- ---------- -----V---P
AcGGP-R-2    ---------- ---------- ---------- ---------- ---------- -----I---- ---RR----- ---------- ---------- ---------- ---------- -----V---P
AdGGP        ---------- ---------- ---------- ---------- ---------- -----I---- ---KR----- ---------- ---------- ---------- ---------- -----V---P
AeGGP        ---------- ---------- ---------- ---------- ---------- -----I---- ---KR----- ---------- ---------- ---------- ---------- -----V---P
ArGGP        ---------- ---------- ---------- ---------- ---------- -----I---- ---KR----- ---------- ---------- ---------- ---------- -----V---P
AmhGGP-1     ---------- ---------- ---------- ---------- ---------- -----I---- ---KR----- ---------- ---------- ---------- ---------- -----V---P
AmhGGP-2     ---------- ---------- ---------- ---------- ---------- -----I---- ---KR----- ---------- ---------- ---------- ---------- -----V---P
AncGGP-3     ---------- ---------- ---------- ---------- ---------- ---------- ---QK----- ---------- ---------- ---------- ---------- --------EH
AncGGP-1     ---------- ---------- ---------- ---------- ---------- -----I---- ---KR----- ---------- ---------- ---------- ---------- -----V---P
AncGGP-2     ---------- ---------- ---------- ---------- ---------- -----I---- ---KR----- ---------- ---------- ---------- ---------- -----V---P
AqcGGP       ---------- ---------- ---------- ---------- ---------- -----I---- ---KR----- ---------- ---------- ---------- ---------- -----V---P
AhGGP-1      ---------- ---------- ---------- ---------- ---------- -----I---- ---KR----- ---------- ---------- ---------- ---------- -----V---P
AhGGP-2      ---------- ---------- ---------- ---------- ---------- -----I---- ---KR----- ---------- ---------- ---------- ---------- -----V---P
AlGGP-1      ---------- ---------- ---------- ---------- ---------- -----I---- ---KR----- ---------- ---------- ---------- ---------- -----V---P
AlGGP-2      ---------- ---------- ---------- ---------- ---------- -----I---- ---KR----- ---------- ---------- ---------- ---------- -----V---P
AtGGP-1      ---------- ---------- ---------- ---------- ---------- -----I---- ---KR----- ---------- ---------- ---------- ---------- -----V---P
AtGGP-2      ---------- ---------- ---------- ---------- ---------- -----I---- ---KR----- ---------- ---------- ---------- ---------- -----V---P
BosGGP-1     ---------- ---------- ---------- ---------- ---------- -----I---- ---KR----- ---------- ---------- ---------- ---------- -----V---P
BosGGP-2     ---------- ---------- ---------- ---------- ---------- -----I---- ---KR----- ---------- ---------- ---------- ---------- -----V---P
BdGGP-1      ---------- ---------- ---------- ---------- ---------- -----I---- ---KR----- ---------- ---------- ---------- ---------- -----V---P
BdGGP-2      ---------- ---------- ---------- ---------- ---------- -----I---- ---KR----- ---------- ---------- ---------- ---------- -----V---P
BdGGP-3      ---------- ---------- ---------- ---------- ---------- ---------- ---------- ---------- ---------- ---------- ---------- ----------
BsGGP-1      ---------- ---------- ---------- ---------- ---------- -----I---- ---KR----- ---------- ---------- ---------- ---------- -----V---P
BsGGP-2      ---------- ---------- ---------- ---------- ---------- -----I---- ---KR----- ---------- ---------- ---------- ---------- -----V---P
BsGGP-3      ---------- ---------- ---------- ---------- ---------- ---------- ---------- ---------- ---------- ---------- ---------- ----------
BoGGP-1      ---------- ---------- ---------- ---------- ---------- -----I---- ---KR----- ---------- ---------- ---------- ---------- -----V---P
BoGGP-2      ---------- ---------- ---------- ---------- ---------- -----I---- ---KR----- ---------- ---------- ---------- ---------- -----V---P
BoGGP-3      ---------- ---------- ---------- ---------- ---------- -----I---- ---KR----- ---------- ---------- ---------- ---------- -----V---L
BrGGP-1      ---------- ---------- ---------- ---------- ---------- -----I---- ---KR----- ---------- ---------- ---------- ---------- -----V---P
BrGGP-2      ---------- ---------- ---------- ---------- ---------- -----I---- ---KR----- ---------- ---------- ---------- ---------- -----V---P
BrGGP-3      ---------- ---------- ---------- ---------- ---------- -----N---- ---KR----- ---------- ---------- ---------- ---------- -----V---P
BrGGP-4      ---------- ---------- ---------- ---------- ---------- -----I---- ---KR----- ---------- ---------- ---------- ---------- -----V---P
CgGGP-1      ---------- ---------- ---------- ---------- ---------- -----I---- ---KR----- ---------- ---------- ---------- ---------- -----V---P
CgGGP-2      ---------- ---------- ---------- ---------- ---------- -----I---- ---KR----- ---------- ---------- ---------- ---------- -----V---A
CrGGP-1      ---------- ---------- ---------- ---------- ---------- -----I---- ---KR----- ---------- ---------- ---------- ---------- -----V---P
CrGGP-2      ---------- ---------- ---------- ---------- ---------- -----I---- ---KR----- ---------- ---------- ---------- ---------- -----V---P
ChrGGP       ---------- ---------- ---------- ---------- ---------- -----L---- ---RQ----- ---------- ---------- ----P----- ---------- -----A---P
CcGGP-1      ---------- ---------- ---------- ---------- ---------- -----I---- ---KR----- ---------- ---------- ---------- ---------- -----V---P
CcGGP-2      ---------- ---------- ---------- ---------- ---------- -----I---- ---KR----- ---------- ---------- ---------- ---------- -----V---P
CsGGP-1      ---------- ---------- ---------- ---------- ---------- -----I---- ---KR----- ---------- ---------- ---------- ---------- -----V---P
CsGGP-2      ---------- ---------- ---------- ---------- ---------- -----I---- ---KR----- ---------- ---------- ---------- ---------- -----V---P
CosGGP       ---------- ---------- ---------- ---------- ---------- -----I---- VVLRR----- ---------- ---------- ---------- ---------- ----------
CusGGP       ---------- ---------- ---------- ---------- ---------- -----I---- ---KR----- ---------- ---------- ---------- ---------- -----V---P
DcGGP-1      ---------- ---------- ---------- ---------- ---------- -----I---- ---KR----- ---------- ---------- ---------- ---------- -----V---P
DcGGP-2      ---------- ---------- ---------- ---------- ---------- -----I---- ---KR----- ---------- ---------- ---------- ---------- -----V---P
DsGGP        ---------- ---------- ---------- ---------- ---------- -----L---- ---KR----- ---------- ---------- ---------- ---------- -----V---A
EgGGP-1      ---------- ---------- ---------- ---------- ---------- -----I---- ---KR----- ---------- ---------- ---------- ---------- -----V---P
EgGGP-2      ---------- ---------- ---------- ---------- ---------- -----I---- ---KR----- ---------- ---------- ---------- ---------- -----V---P
EgGGP-3      ---------- ---------- ---------- ---------- ---------- -----I---- ---KR----- ---------- ---------- ---------- ---------- -----A---A
EgGGP-4      ---------- ---------- ---------- ---------- ---------- -----I---- ---KR----- ---------- ---------- ---------- ---------- -----V---A
EgGGP-5      ---------- ---------- ---------- ---------- ---------- -----I---- ---KR----- ---------- ---------- ---------- ---------- -----V---A
EsGGP-1      ---------- ---------- ---------- ---------- ---------- -----I---- ---KR----- ---------- ---------- ---------- ---------- -----V---P
EsGGP-2      ---------- ---------- ---------- ---------- ---------- -----I---- ---KR----- ---------- ---------- ---------- ---------- -----V---P
FvGGP-1      ---------- ---------- ---------- ---------- ---------- -----I---- ---KR----- ---------- ---------- ---------- ---------- -----V---P
FvGGP-2      ---------- ---------- ---------- ---------- ---------- -----I---- ---KR----- ---------- ---------- ---------- ---------- -----V---P
FvGGP-3      ---------- ---------- ---------- ---------- ---------- -----I---- ---KK----- ---------- ---------- ---------- ---------- -----V---P
GmGGP-1      ---------- ---------- ---------- ---------- ---------- -----I---- ---KR----- ---------- ---------- ---------- ---------- -----V---P
GmGGP-2      ---------- ---------- ---------- ---------- ---------- -----I---- ---KR----- ---------- ---------- ---------- ---------- -----V---P
GmGGP-3      ---------- ---------- ---------- ---------- ---------- -----I---- ---KR----- ---------- ---------- ---------- ---------- -----V---P
GmGGP-4      ---------- ---------- ---------- ---------- ---------- -----I---- ---KR----- ---------- ---------- ---------- ---------- -----V---P
GnmGGP       ---------- ---------- ---------- ---------- ---------- -----I---- ---KR----- ---------- ---------- ---------- ---------- -----C---P
GrGGP-1      ---------- ---------- ---------- ---------- ---------- -----I---- ---KR----- ---------- ---------- ---------- ---------- -----V---P
GrGGP-2      ---------- ---------- ---------- ---------- ---------- -----I---- ---KR----- ---------- ---------- ---------- ---------- -----V---P
GrGGP-3      ---------- ---------- ---------- ---------- ---------- -----I---- ---KR----- ---------- ---------- ---------- ---------- -----V---P
KfGGP-1      ---------- ---------- ---------- ---------- ---------- -----I---- ---KR----- ---------- ---------- ---------- ---------- -----V---P
KfGGP-2      ---------- ---------- ---------- ---------- ---------- -----I---- ---KR----- ---------- ---------- ---------- ---------- -----V---P
KlGGP-1      ---------- ---------- ---------- ---------- ---------- -----I---- ---KR----- ---------- ---------- ---------- ---------- -----V---P
KlGGP-2      ---------- ---------- ---------- ---------- ---------- -----I---- ---KR----- ---------- ---------- ---------- ---------- -----V---P
KlGGP-3      ---------- ---------- ---------- ---------- ---------- -----I---- ---KR----- ---------- ---------- ---------- ---------- -----V---P
LuGGP        ---------- ---------- ---------- ---------- ---------- -----I---- ---KR----- ---------- ---------- ---------- ---------- -----V---P
MdGGP        ---------- ---------- ---------- ---------- ---------- -----I---- ---KR----- ---------- ---------- ---------- ---------- -----V---P
MeGGP-1      ---------- ---------- ---------- ---------- ---------- -----I---- ---KR----- ---------- ---------- ---------- ---------- -----V---P
MeGGP-2      ---------- ---------- ---------- ---------- ---------- -----I---- ---KR----- ---------- ---------- ---------- ---------- -----V---P
MeGGP-3      ---------- ---------- ---------- ---------- ---------- -----I---- ---KK----- ---------- ---------- ---------- ---------- -----V---P
MpGGP        ---------- ---------- ---------- ---------- ---------- -----I---- ---RR----- ---------- ---------- ---------- ---------- -----V---S
MtGGP-1      ---------- ---------- ---------- ---------- ---------- -----I---- ---KR----- ---------- ---------- ---------- ---------- -----V---P
```

```
MtGGP-2    ---------- ---------- ---------- ---------- ---------- -----I---- ---KR----- ---------- ---------- ---------- ---------- -----V---P
MpGGP      ---------- ---------- ---------- ---------- ---------- -----I---- ---KR----- ---------- ---------- ---------- ---------- -----V---E
MsGGP      ---------- ---------- ---------- ---------- ---------- -----I---- ---KK----- ---------- ---------- ---------- ---------- -----I---P
MgGGP-1    ---------- ---------- ---------- ---------- ---------- -----I---- ---KR----- ---------- ---------- ---------- ---------- -----V---P
MgGGP-2    ---------- ---------- ---------- ---------- ---------- -----I---- ---KR----- ---------- ---------- ---------- ---------- -----V---P
MaGGP-1    ---------- ---------- ---------- ---------- ---------- -----I---- ---KR----- ---------- ---------- ---------- ---------- -----V---P
MaGGP-2    ---------- ---------- ---------- ---------- ---------- -----I---- ---KR----- ---------- ---------- ---------- ---------- -----V---P
MaGGP-3    ---------- ---------- ---------- ---------- ---------- -----I---- ---KR----- ---------- ---------- ---------- ---------- -----V---P
MaGGP-4    ---------- ---------- ---------- ---------- ---------- ---------- ---------- ---------- ---------- ---------- ---------- ----------
OtGGP      ---------- ---------- ---------- ---------- ---------- -----I---- ---RR----- ---------- ---------- ---------- ---------- -----V---P
OsGGP-1    ---------- ---------- ---------- ---------- ---------- -----I---- ---KR----- ---------- ---------- ---------- ---------- -----V---P
OsGGP-2    ---------- ---------- ---------- ---------- ---------- -----L---- ---AH----- ---------- ---------L APSS------ ---------- -----V--AP
OlGGP      ---------- ---------- ---------- ---------- ---------- ---------- ---------- ---------- ---------- ---------- ---------- ----------
PhGGP-1    ---------- ---------- ---------- ---------- ---------- -----I---- ---RR----- ---------- ---------- ---------- ---------- -----V---P
PhGGP-2    ---------- ---------- ---------- ---------- ---------- ---------- ---------- ---------- ---------- ---------- ---------- ----------
PvGGP-1    ---------- ---------- ---------- ---------- ---------- -----I---- ---RR----- ---------- ---------- ---------- ---------- -----V---P
PvGGP-2    ---------- ---------- ---------- ----SC---- ---------- ----AVF--A CLHERL---- ---------- ----SQAMLC LPARTPE--- --------PG YAHATV---P
PvGGP-3    ---------- ---------- ---------- ---------- ---------- ---------- ---------- ---------- ---------- ---------- ---------- ----------
PhvGGP-1   ---------- ---------- ---------- ---------- ---------- -----I---- ---KR----- ---------- ---------- ---------- ---------- -----V---P
PhvGGP-2   ---------- ---------- ---------- ---------- ---------- -----I---- ---KR----- ---------- ---------- ---------- ---------- -----V---P
PpGGP-1    ---------- ---------- ---------- ---------- ---------- -----I---- ---KR----- ---------- ---------- ---------- ---------- -----V---E
PpGGP-2    ---------- ---------- ---------- ---------- ---------- -----I---- ---RR----- ---------- ---------- ---------- ---------- -----V---E
PpGGP-3    ---------- ---------- ---------- ---------- ---------- -----I---- ---RR----- ---------- ---------- ---------- ---------- -----V---E
PaGGP-1    ---------- ---------- ---------- ---------- ---------- -----I---- ---KR----- ---------- ---------- ---------- ---------- -----C---P
PaGGP-2    AGGALESTPS PHGGRGALPS EGGSPSDLLF LAGGGCDRAL FCLAHPFLLS RGEIIW---- ---ER----- ---------- ---------- ---------- ---------- -----C---P
PaGGP-3    ---------- ---------- ---------- ---------- ---------- -----I---- ---RRRRFLR SRNAIAALER TTSPHGGRGA LPSEGGSPSD LQFLAGGGRG LALF-CLAHP
PsGGP-1    ---------- ---------- ---------- ---------- ---------- -----I---- ---KR----- ---------- ---------- ---------- ---------- -----C---P
PsGGP-2    ---------- ---------- ---------- ---------- ---------- -----I---- ---KR----- ---------- ---------- ---------- ---------- -----C---P
PsGGP-3    ---------- ---------- ---------- ---------- ---------- -----I---- ---KR----- ---------- ---------- ---------- ---------- -----C---P
PtGGP-1    ---------- ---------- ---------- ---------- ---------- -----I---- ---KR----- ---------- ---------- ---------- ---------- -----C---P
PtGGP-2    ---------- ---------- ---------- ---------- ---------- -----I---- ---KR----- ---------- ---------- ---------- ---------- -----C---P
PtGGP-3    ---------- ---------- ---------- ---------- ---------- -----I---- ---KR----- ---------- ---------- ---------- ---------- -----C---P
PtGGP-4    ---------- ---------- ---------- ---------- ---------- -----I---- ---KR----- ---------- ---------- ---------- ---------- -----C---P
PtGGP-5    ---------- ---------- ---------- ---------- ---------- -----I---- ---KR----- ---------- ---------- ---------- ---------- -----C---P
PotGGP-1   ---------- ---------- ---------- ---------- ---------- -----I---- ---KR----- ---------- ---------- ---------- ---------- -----V---P
PotGGP-2   ---------- ---------- ---------- ---------- ---------- -----I---- ---KR----- ---------- ---------- ---------- ---------- -----V---P
PotGGP-3   ---------- ---------- ---------- ---------- ---------- -----I---- ---KK----- ---------- ---------- ---------- ---------- -----V---P
PrpGGP     ---------- ---------- ---------- ---------- ---------- -----I---- ---KR----- ---------- ---------- ---------- ---------- -----V---P
RcGGP-1    ---------- ---------- ---------- ---------- ---------- -----I---- ---KR----- ---------- ---------- ---------- ---------- -----V---P
RcGGP-2    ---------- ---------- ---------- ---------- ---------- ---------- ---------- ---------- ---------- ---------- ---------- ----------
RcGGP-3    ---------- ---------- ---------- ---------- ---------- -----I---- ---KK----- ---------- ---------- ---------- ---------- -----V---P
SpGGP-1    ---------- ---------- ---------- ---------- ---------- -----I---- ---KR----- ---------- ---------- ---------- ---------- -----V---P
SpGGP-2    ---------- ---------- ---------- ---------- ---------- -----I---- ---KR----- ---------- ---------- ---------- ---------- -----V---P
SpGGP-3    ---------- ---------- ---------- ---------- ---------- -----I---- ---KK----- ---------- ---------- ---------- ---------- -----V---P
SmGGP-1    ---------- ---------- ---------- ---------- ---------- -----I---- ---RR----- ---------- ---------- ---------- ---------- -----I---P
SmGGP-2    ---------- ---------- ---------- ---------- ---------- -----M---- ---------- ---------- ---------- ---------- ---------G -----F--EA
SmGGP-3    ---------- ---------- ---------- ---------- ---------- -----I---- ---KR----- ---------- ---------- ---------- ---------- -----V---P
SiGGP-1    ---------- ---------- ---------- ---------- ---------- -----I---- ---RR----- ---------- ---------- ---------- ---------- -----V---P
SiGGP-2    ---------- ---------- ---------- ---------- ---------- -----I---- ---AP----- ---------- ---------L APSL------ ---------- -----V--AP
SvGGP-1    ---------- ---------- ---------- ---------- ---------- -----I---- ---RR----- ---------- ---------- ---------- ---------- -----V---P
SvGGP-2    ---------- ---------- ---------- ---------- ---------- -----I---- ---AP----- ---------- ---------L APSL------ ---------- -----V--AP
SlGGP-1    ---------- ---------- ---------- ---------- ---------- -----I---- ---KR----- ---------- ---------- ---------- ---------- -----V---P
SlGGP-2    ---------- ---------- ---------- ---------- ---------- -----V---- ---KR----- ---------- ---------- ---------- ---------- -----V---P
StGGP-1    ---------- ---------- ---------- ---------- ---------- -----I---- ---KR----- ---------- ---------- ---------- ---------- -----V---P
StGGP-2    ---------- ---------- ---------- ---------- ---------- -----I---- ---KR----- ---------- ---------- ---------- ---------- -----V---P
SbGGP-1    ---------- ---------- ---------- ---------- ---------- -----I---- ---KR----- ---------- ---------- ---------- ---------- -----V---P
SbGGP-2    ---------- ---------- ---------- ---------- ---------- -----L---- ---AQ----- ---------- ---------L IPSL------ ---------- -----V--AP
SfGGP-1    ---------- ---------- ---------- ---------- ---------- -----I---- ---KR----- ---------- ---------- ---------- ---------- -----V---A
SfGGP-2    ---------- ---------- ---------- ---------- ---------- -----I---- ---KR----- ---------- ---------- ---------- ---------- -----V---A
SppGGP-1   ---------- ---------- ---------- ---------- ---------- -----I---- ---KR----- ---------- ---------- ---------- ---------- -----V---P
SppGGP-2   ---------- ---------- ---------- ---------- ---------- -----I---- ---KR----- ---------- ---------- ---------- ---------- -----V---P
TcGGP-1    ---------- ---------- ---------- ---------- ---------- -----I---- ---KR----- ---------- ---------- ---------- ---------- -----V---P
TcGGP-2    ---------- ---------- ---------- ---------- ---------- -----I---- ---KR----- ---------- ---------- ---------- ---------- -----V---A
TpGGP      ---------- ---------- ---------- ---------- ---------- -----I---- ---KR----- ---------- ---------- ---------- ---------- -----V---P
VvGGP-1    ---------- ---------- ---------- ---------- ---------- -----I---- ---KR----- ---------- ---------- ---------- ---------- -----V---P
VvGGP-2    ---------- ---------- ---------- ---------- ---------- -----I---- ---KR----- ---------- ---------- ---------- ---------- -----V---P
VcGGP      ---------- ---------- ---------- ---------- ---------- -----LHRQS GAWSR----- ---------- ---------- ---------- ---------- -----L---P
ZmGGP-E-1  ---------- ---------- ---------- ---------- ---------- -----I---- ---RR----- ---------- ---------- ---------- ---------- -----V---P
ZmGGP-E-2  ---------- ---------- ---------- ---------- ---------- -----L---- ---RR----- ---------- ---------- ---------- ---------- -----VVMEP
ZmGGP-1    ---------- ---------- ---------- ---------- ---------- -----I---- ---RR----- ---------- ---------- ---------- ---------- -----V---P
ZmGGP-2    ---------- ---------- ---------- ---------- ---------- -----L---- ---RR----- ---------- ---------- ---------- ---------- -----VVMEP
ZomGGP     ---------- ---------- ---------- ---------- ---------- -----I---- ---KR----- ---------- ---------- ---------- ---------- -----V---P

           ....|....| ....|....| ....|....| ....|....| ....|....| ....|....| ....|....| ....|....| ....|....| ....|....| ....|....| ....|....|
                  250        260        270        280        290        300        310        320        330        340        350        360
AcGGP      -------TVV SNFQK----- ---------- --------DE AED------- ---------- ----GARS-- ---------- G--------- GGCGRNC--- ----LQKCC- ----------
AcGGP-R-1  -------TVV SNFQK----- ---------- --------DE ADD------- ---------- ----GARS-- ---------- G--------- GGCGRNC--- ----LQKCC- ----------
AcGGP-R-2  -------TVV SNYQE----- ---------- --------DV SED------- ---------- PQFQSDSG-- ---------- T--------- LGCGRNC--- ----LSKCC- ----------
AdGGP      -------TVV SNFQK----- ---------- --------DE AED------- ---------- ----GARS-- ---------- G--------- GGCGRNC--- ----LQKCC- ----------
AeGGP      -------TVV SNFQK----- ---------- --------DE ADD------- ---------- ----GARS-- ---------- G--------- GGCGRNC--- ----LQKCC- ----------
ArGGP      -------TVV SNFQK----- ---------- --------DE ADD------- ---------- ----GARS-- ---------- G--------- GGCGRNC--- ----LQKCC- ----------
AmhGGP-1   -------TIV SNYQK----- ---------- --------EE SDG------- ---------- ----SSRS-- ---------- V--------- GGCGRNC--- ----LKECC- ----------
AmhGGP-2   -------TVL SNYQE----- ---------- --------ED G--------- ---------- ----GASE-- ---------- N--------- LGCGRNC--- ----LGKCC- ----------
AncGGP-3   -------EMV SEESG----- -------G-- ---------- ---------- ---------- ----GGEG-- ---------- G--------- RGCGRSC--- ----LSKCCL ----------
AncGGP-1   -------TVV SNYRE----- ---------- --------DA ---------- ---------- -----PAE-- ---------- A--------- AGCGRNC--- ----LGKCC- ----------
AncGGP-2   -------TVV SNYQE----- ---------- --------DT VDD------- ---------- ----AAAA-- ---------- ---------- AAAEPVG--- ----LGRCC- ----------
AqcGGP     -------TVV SNYQK----- ---------- --------DG EED------- ---------- ----ATVF-- ---------- T--------- GGCGRNC--- ----LRHCC- ----------
AhGGP-1    -------TVV SNYQK----- ---------- --------DD GAD------- ---------- ----D----- ---------- S--------- VGCGRNC--- ----LGACC- ----------
AhGGP-2    -------TVV SNYQK----- ---------- --------DE TVE------- ---------- ----E----- ---------- ---------- GGCGRNC--- ----LSKCC- ----------
AlGGP-1    -------TVV SNYQK----- ---------- --------DD GAD------- ---------- ----D----- ---------- S--------- VGCGRNC--- ----LGACC- ----------
AlGGP-2    -------TVV SNYQK----- ---------- --------DE TVE------- ---------- ----E----- ---------- ---------- GGCGRNC--- ----LSKCC- ----------
AtGGP-1    -------TVV SNYQK----- ---------- --------DD GAE------- ---------- ----D----- ---------- P--------- VGCGRNC--- ----LGACC- ----------
AtGGP-2    -------TVV SNYQK----- ---------- --------DE TVE------- ---------- ----E----- ---------- ---------- GGCGRNC--- ----LSKCC- ----------
BosGGP-1   -------TVV SNYQK----- ---------- --------DE AAD------- ---------- ----E----- ---------- S--------- VGCGRNC--- ----LGACC- ----------
BosGGP-2   -------TVV SNYQK----- ---------- --------DE TVE------- ---------- ----E----- ---------- ---------- GGCGRSC--- ----LNKCC- ----------
BdGGP-1    -------TIV SNYQE----- ---------- --------EA AA-------- ---------- -----ERP-- ---------- R--------- GGCGKNC--- ----LGDCC- ----------
BdGGP-2    -------TVL SNYQE----- ---------- --------EG G--------- ---------- ---------- ---------- G--------- AGCGRNC--- ----LGDCC- ----------
BdGGP-3    ---------- ---------- ---------- ---------- ---------- ---------- ---------- ---------- ---------- ---------- ---------- ----------
BsGGP-1    -------TIV SNYQE----- ---------- --------DA AA-------- ---------- -----ERP-- ---------- R--------- GGCGKNC--- ----LGDCC- ----------
BsGGP-2    -------TVL SNYQE----- ---------- --------EG GGG------- ---------- ----GGGG-- ---------- G--------- AGCGRNC--- ----LGDCC- ----------
BsGGP-3    ---------- ---------- ---------- ---------- ---------- ---------- ---------- ---------- ---------- ---------- ---------- ----------
BoGGP-1    -------TVV SNYQK----- ---------- --------DE ASD------- ---------- ----E----- ---------- S--------- VGCGRNC--- ----LGACC- ----------
BoGGP-2    -------MVL SAAVD----- ---------- --------GT ASE------- ---------- ----L----- ---------- V--------- ---------- -------VL- ----------
BoGGP-3    -------TLV SNHQK----- ---------- --------DE TAE------- ---------- ----E-EE-- ---------- G--------- EGCGRNI--- ----LSKCC- ----------
BrGGP-1    -------TVV SNYQK----- ---------- --------DE SAD------- ---------- ----E----- ---------- S--------- VGCGRNC--- ----LGACC- ----------
BrGGP-2    -------TVV SNYQK----- ---------- --------DE ASD------- ---------- ----E----- ---------- S--------- VGCGRNC--- ----LGACC- ----------
BrGGP-3    -------TVV SNYQK----- ---------- --------NE ASD------- ---------- ----G----- ---------- S--------V GGCGRNC--- ----LRACC- ----------
BrGGP-4    -------AVV SNHQK----- ---------- --------DE TAE------- ---------- ----E-EE-- ---------- G--------- EGCGRNI--- ----LSKCC- ----------
CgGGP-1    -------TVV SNYQK----- ---------- --------DE AAD------- ---------- ----E----- ---------- S--------- VGCGRNC--- ----LGACC- ----------
CgGGP-2    -------TVV SNYQK----- ---------- --------DE TVE------- ---------- ----E----- ---------- ---------- GGCGRNC--- ----LSKCC- ----------
CrGGP-1    -------TVV SNYQK----- ---------- --------DE AAD------- ---------- ----E----- ---------- S--------- VGCGRNC--- ----LGACC- ----------
CrGGP-2    -------TVV SNYQK----- ---------- --------DE TVE------- ---------- ----E----- ---------- ---------- GGCGRNC--- ----LRKCC- ----------
ChrGGP     --ASAPATVA SCLEAHVPVY AFPATGV--- ------WVDS ALGMVAPVAK LARIDSMPAV PDYFAEARCD S---PPPTGL ALPEYG-LGV PGSDSPCCAG TG--SSDCAS -----PAVED
CcGGP-1    -------TVV SNYQK----- ---------- --------EE TEE------- ---------- -AAAAARR-- ---------- V--------- SGCGRNC--- ----LNKCC- ----------
CcGGP-2    -------TVV SNYQD----- ---------- --------QE ETA------- ---------- ----SENS-- ---------- E--------- AGCGSNC--- ----LGNCS- ----------
CsGGP-1    -------TVV SNYQK----- ---------- --------EE TEE------- ---------- -AAAAARR-- ---------- V--------- SGCGRNC--- ----LNKCC- ----------
```

```
CsGGP-2      -------TVV SNYQD----- ---------- --------QE ETA------- ---------- ----SENS-- ---------- E--------- AGCGSNC--- ----LGNCS- ----------
CosGGP       -------SWL SGSSD----- ---------- --------EE GS-------- ---------- ---------- ---------- ---------- ---------- ---------- ----------
CusGGP       -------TVV SNYQE----- ---------- --------DE TEN------- ---------- ----VSR--- ---------- S--------- TGCGRNC--- ----LNKCC- ----------
DcGGP-1      -------TIV SNYQK----- ---------- --------EE GEE------- ---------- ----GACR-- ---------- ---------- VGCGRNC--- ----LNTCC- ----------
DcGGP-2      -------TVV SNYQK----- ---------- --------ED GEE------- ---------- ----ASRQ-- ---------T T--------- GGCGRNC--- ----LNNCC- ----------
DsGGP        -------TVV SMKQL----- ---------- --------AQ DDS------- ---------- ---------- ---------- ---------- GGSGGNAPPS NPESISQC-- ----------
EgGGP-1      -------TVV SNYQK----- ---------- --------EE VEG------- ---------- ----G--R-- ---------- V--------- GGCGRNC--- ----LNKCC- ----------
EgGGP-2      -------TVV SNYQK----- ---------- --------EE AEE------- ---------- ----GARR-- ---------- G--------- GGCGRNC--- ----LNKCC- ----------
EgGGP-3      -------TVV SNYQE----- ---------- --------EG ---------- ---------- ------GE-- ---------- A--------- KGCGRNC--- ----LGKCC- ----------
EgGGP-4      -------TVV SNYQE----- ---------- --------GG EKD-N----- ---------- AAVAVEGE-- ---------- T--------- KGCGRNC--- ----LGKCC- ----------
EgGGP-5      -------TVV SNYQE----- ---------- --------EG GEKDN----- ---------- AAVAVEGE-- ---------- A--------- KGCGRNC--- ----LGKCC- ----------
EsGGP-1      -------TVV SNYQK----- ---------- --------DE AAD------- ---------- ----E----- ---------- S--------- VGCGRNC--- ----LGACC- ----------
EsGGP-2      -------TVV SNYQK----- ---------- --------DE TAE------- ---------- ----E----- ---------- G--------- GGCGRNC--- ----LSKCC- ----------
FvGGP-1      -------TVV SNYQK----- ---------- --------DE ADE------- ---------- ----G-RR-- ---------- A--------- GGCGRNC--- ----LNKCC- ----------
FvGGP-2      -------TVV SNYQK----- ---------- --------DE ADE------- ---------- ----G-RR-- ---------- A--------- GGCGRNC--- ----LNKCC- ----------
FvGGP-3      -------TVV SNYQN----- ---------- --------DE ADE------- ---------- ----G-RR-- ---------- V--------- GGCGRNC--- ----LNKCC- ----------
GmGGP-1      -------TVV SNFQK----- ---------- --------DD AAD------- ---------- ----SPRP-- ---------- V--------- GGCGRNC--- ----LKACC- ----------
GmGGP-2      -------TVV SNYQK----- ---------- --------DD AAD------- ---------- ----SPRP-- ---------- V--------- GGCGRNC--- ----LKACC- ----------
GmGGP-3      -------TVV SNYQK----- ---------- --------EE TGE------- ---------- ----AA---- ---------- A--------- GGCGRNC--- ----LKSCC- ----------
GmGGP-4      -------TVV SNYQK----- ---------- --------EE TGE------- ---------- ----AA-A-- ---------- A--------- GGCGRNC--- ----LKSCC- ----------
GnmGGP       -------TIV SMYHD----- ---------- ---------- ---------- ---------- ----GSEA-- ---------- A--------- AGCGRNC--- ----LGQCC- ----------
GrGGP-1      -------TVV SNYQK----- ---------- --------DE ADD------- ---------- ----TARR-- ---------- T--------- AGCGKNC--- ----LKSCC- ----------
GrGGP-2      -------TVV SNYQK----- ---------- --------DE TEE------- ---------- ----TARR-- ---------- S--------- SGCGKNC--- ----LRSCC- ----------
GrGGP-3      -------TVV SNYQK----- ---------- --------DE AEE------- ---------- ----TARR-- ---------- S--------- GGCGKNC--- ----LRSCC- ----------
KfGGP-1      -------TVV SNYQK----- ---------- --------DE ADE------- ---------- ----P----- ---------- ---------- KGCGRNC--- ----LKQCC- ----------
KfGGP-2      -------TVL SNYQK----- ---------- --------DE ADE------- ---------- ----P----- ---------- ---------- KGCGRQC--- ----LKECC- ----------
KlGGP-1      -------TVV SNYQK----- ---------- --------DE ADE------- ---------- ----P----- ---------- ---------- KGCGRNC--- ----LKQCC- ----------
KlGGP-2      -------TVL SNYQK----- ---------- --------DE ADE------- ---------- ----P----- ---------- ---------- KGCGRQC--- ----LKECC- ----------
KlGGP-3      -------TVL SNYQK----- ---------- --------DE ADE------- ---------- ----P----- ---------- ---------- KGCGRQC--- ----LKECC- ----------
LuGGP        -------TVV SNYQK----- ---------- --------EE TED------- ---------- ----A----- ---------- G--------- GGCGRNC--- ----LQNCC- ----------
MdGGP        -------TVV SNYQK----- ---------- --------DE AEE------- ---------- ----GARR-- ---------- V--------- EGCGRNC--- ----LNQCC- ----------
MeGGP-1      -------TLV SNFQK----- ---------- ---------E AEE------- ---------- ----GASR-- ---------- S--------- GGCGRNC--- ----LQKCC- ----------
MeGGP-2      -------TVV SNFQK----- ---------- --------EE AEE------- ---------- ----GARR-- ---------- S--------- EGCGRNC--- ----LRKCC- ----------
MeGGP-3      -------TVV SNYQE----- ---------- --------ES SE-------- ---------- ------TS-- ---------- F--------- EGCGRHC--- ----LGKCC- ----------
MpGGP        -------TVV SINQV----- ---------- --------SG AAD------- ---------- ----SSS--- ---------- ---------- ---------- ---------- ----------
MtGGP-1      -------TVV SNYQK----- ---------- --------EE VGE------- ---------- ---AAPRT-- ---------- V--------- GGCGRNC--- ----LKSCC- ----------
MtGGP-2      -------TVV SNYQK----- ---------- --------DE DAP------- ---------- ---------- ---------- V--------- NGCGRNC--- ----LKSCC- ----------
MpGGP        -------TMI SMHQM----- ---------- --------DD EDD------- ---------- PK-------- ---------- ---------- ----ANT--- ----VARVV- ----------
MsGGP        -------TVL SIHQM----- ---------- --------DE EED------- ---------- PKF------- ---------- ---------- ----AGA--- ----VRRCC- ----------
MgGGP-1      -------TVV SNYQK----- ---------- --------ED AEE------- ---------- ----G----- ---------- G--------- AGCGRNC--- ----LRSCC- ----------
MgGGP-2      -------TVV SNYQK----- ---------- --------DE AEE------- ---------- ----GVRP-- ---------- V--------- TGCGRNC--- ----FRSCC- ----------
MaGGP-1      -------TVL SNYQE----- ---------- --------DS ---------- ---------- ------GE-- ---------- S--------- RGCGRNC--- ----LGKCC- ----------
MaGGP-2      -------TVL SNYQE----- ---------- --------EN ---------- ---------- -----AGE-- ---------- P--------- LGCGRSC--- ----LGKCC- ----------
MaGGP-3      -------TVL SNYQE----- ---------- --------DS ---------- ---------- ------GE-- ---------- P--------- RGCDRNC--- ----LSKCC- ----------
MaGGP-4      ---------M SVYQP----- ---------- --------TQ GQH------- ---------- ---------G ---------- E--------- EKSGRSC--- ----LLQVH- ----------
OtGGP        -------TVV SNYQE----- ---------- --------DS AAA------- ---------- ----A----- ---------- A--------- GGCGRNC--- ----LGDCC- ----------
OsGGP-1      -------TVV SNYQE----- ---------- --------DA AAT------- ---------- ---AGERP-- ---------- R--------- AGCGRDC--- ----LGDCC- ----------
OsGGP-2      -----AAFGA ---------- ---------- ---------- ---------- -------RP- ----ARRR-- ---------- ---------- ---------- ---------- ----------
OlGGP        ---------- ---------- ---------- ---------- ---------- ---------- ---------- ---------- ---------- ---------- ---------- ----------
PhGGP-1      -------TVV SNYQD----- ---------- --------DA ---------- ---------- -----DKP-- ---------- R--------- AGCGRNC--- ----LGHCC- ----------
PhGGP-2      ---------- ---------- ---------- ---------- ---------- ---------- ---------- ---------- ---------- ---------- ---------- ----------
PvGGP-1      -------TVV SNYQE----- ---------- --------DA ---------- ---------- -----DKP-- ---------- R--------- AGCGRNC--- ----LGHCC- ----------
PvGGP-2      LEPGSRETVD SGVST----- -----GPGSC GAGMSCLGEC AEEKRL---- LS-------- ADCAGNQTIS VL--SLAAWI RKPIMGLLHL ANLAKGS--- ----LHRCS- ----------
PvGGP-3      ---------- ---------- ---------- ---------- ---------- ---------- ---------- ---------- ---------- ---------- ---------- ----------
PhvGGP-1     -------TVV SNYQK----- ---------- --------DG AAD------- ---------- ----TSRP-- ---------- V--------- GGCGRNC--- ----LKACC- ----------
PhvGGP-2     -------TVV SNYQK----- ---------- --------EG SDE------- ---------- ----SP---- ---------- A--------- EGCGRNC--- ----LKSCC- ----------
PpGGP-1      -------TVV SMHQQ----- ---------- --------LE ---------- ---------- -------Q-- ---------- V--------- PGCGRDC--- ----LGTCC- ----------
PpGGP-2      -------TLV SINQQ----- ---------- --------LD ---------- ---------- -------Q-- ---------- A--------- PGCGRDC--- ----LGNCC- ----------
PpGGP-3      -------TVV SMQQQ----- ---------- --------LD ---------- ---------- -------Q-- ---------- G--------- SGCGRDC--- ----LGNCC- ----------
PaGGP-1      -------TIL SNYQE----- ---------- ---------- ---------- ---------- -----GSE-- ---------- G--------- LGCGRNC--- ----LGQCC- ----------
PaGGP-2      -------TVL SNYQE----- ---------- ---------- ---------- ---------- -----GSE-- ---------- G--------- LGCGRSC--- ----LGQCC- ----------
PaGGP-3      -------FLL STGEE----- ---------- ---------- ---------- ---------- -----GSE-- ---------- G--------- LGCGRNC--- ----LGQCC- ----------
PsGGP-1      -------TIL SNYQE----- ---------- ---------- ---------- ---------- -----GSE-- ---------- G--------- LGCGRNC--- ----LGQCC- ----------
PsGGP-2      -------TIV SNYQE----- ---------- ---------- ---------- ---------- -----GSE-- ---------- G--------- LGCGRNC--- ----LGQCC- ----------
PsGGP-3      -------TIL SNYQE----- ---------- ---------- ---------- ---------- -----GSE-- ---------- G--------- LGCGRNC--- ----LGQCC- ----------
PtGGP-1      -------TIL SNYQE----- ---------- ---------- ---------- ---------- -----GSK-- ---------- G--------- LGCGRNC--- ----LGQCC- ----------
PtGGP-2      -------TIL SNYQE----- ---------- ---------- ---------- ---------- -----GSE-- ---------- G--------- LGCGRNC--- ----LGQCC- ----------
PtGGP-3      -------TIL SNYQE----- ---------- ---------- ---------- ---------- -----GSE-- ---------- G--------- FGCGRNC--- ----LGQCC- ----------
PtGGP-4      -------TIV SNYQE----- ---------- ---------- ---------- ---------- -----GSE-- ---------- G--------- VGCGRNC--- ----LGQCC- ----------
PtGGP-5      -------TTL SNYQE----- ---------- ---------- ---------- ---------- -----GSE-- ---------- G--------- LGCGRNC--- ----LGQCC- ----------
PotGGP-1     -------TVV SNYQK----- ---------- --------ED GNE------- ---------- ----GSRR-- ---------- G--------- GGCGRNC--- ----LQNCC- ----------
PotGGP-2     -------TVV SNYQK----- ---------- --------EE GEE------- ---------- ----GSRR-- ---------- G--------- GGCGRNC--- ----LQNCC- ----------
PotGGP-3     -------TVV SNYQE----- ---------- --------EN SE-------- ---------- ------KG-- ---------- F--------- EGCGRNC--- ----LGKCC- ----------
PrpGGP       -------TVV SNYQK----- ---------- --------DE AEE------- ---------- ----GARR-- ---------- V--------- GGCGRNC--- ----LNQCC- ----------
RcGGP-1      -------TVV SNFQK----- ---------- --------DE AED------- ---------- ----GGKK-- ---------- S--------- GGCGRNC--- ----LQKCC- ----------
RcGGP-2      ---------- ---------- ---------- ---------- ---------- ---------- ---------- ---------- ---------- ---------- ---------- ----------
RcGGP-3      -------TVV SNYQE----- ---------- --------DD SSD------- ---------- ------TS-- ---------- F--------- EGCGRDC--- ----LGNCC- ----------
SpGGP-1      -------TVV SNYQK----- ---------- --------EE GEE------- ---------- ----GSRR-- ---------- G--------- GGCGRNC--- ----LRNCC- ----------
SpGGP-2      -------TVV SNYQK----- ---------- --------ED GEE------- ---------- ----GSRR-- ---------- G--------- GGCGRNC--- ----LQNCC- ----------
SpGGP-3      -------TVV SNYQE----- ---------- --------NS E--------- ---------- ------KG-- ---------- F--------- EGCGRNC--- ----LGKCC- ----------
SmGGP-1      -------TIV SNYQE----- ---------- ---------- ---------- ---------- ----SLES-- ---------- K--------- IGCGKNC--- ----LGSCC- ----------
SmGGP-2      -------AIV SNDRS----- ---------- ---------- ---------- ---------- ------ET-- ---------- N--------- HGCGRNC--- ----LGPCC- ----------
SmGGP-3      -------TVV SVNQD----- ---------- ---------- ---------- ---------- ---------- ---------- C--------- GSVSRDC--- ----LQSCF- ----------
SiGGP-1      -------TVV SNYQE----- ---------- --------DA ---------- ---------- -----DKP-- ---------- R--------- AGCGRNC--- ----LGDCC- ----------
SiGGP-2      -----APAAS ---------- ---------- ---------- ---------- -------RSR QYETSRRR-- ---------- ---------- ---------- ---------- ----------
SvGGP-1      -------TVV SNYQE----- ---------- --------DA ---------- ---------- -----DKP-- ---------- R--------- AGCGRNC--- ----LGDCC- ----------
SvGGP-2      -----APAAS ---------- ---------- ---------- ---------- -------RSR QYETSRRR-- ---------- ---------- ---------- ---------- ----------
SlGGP-1      -------TLV SNFQK----- ---------- --------DE ADE------- ---------- ----IAAR-- ---------- G--------- AGCGRNC--- ----LRNCC- ----------
SlGGP-2      -------TLV SNYQE----- ---------- --------DV LEG------- ---------- -------N-- ---------- V--------- MGCGRKC--- ----LGKCC- ----------
StGGP-1      -------TLV SNFQK----- ---------- --------DE ADE------- ---------- ----IGAR-- ---------- G--------- AGCGRNC--- ----LRNCC- ----------
StGGP-2      -------TLV SNYQE----- ---------- --------DV LEG------- ---------- -------N-- ---------- V--------- MGCGRKC--- ----LGKCC- ----------
SbGGP-1      -------TVV SNYQD----- ---------- --------DA ---------- ---------- -----DKP-- ---------- R--------- AGCGRNC--- ----LGDCC- ----------
SbGGP-2      -----APTAS ---------- ---------- ---------- ---------- -------RAL RNRTSQRR-- ---------- ---------- ---------- ---------- ----------
SfGGP-1      -------TLV SINQE----- ---------- --------LE ---------- ---------- -------Q-- ---------- A--------- AGCGKDC--- ----LGSCC- ----------
SfGGP-2      -------TLV SFNQE----- ---------- --------LE ---------- ---------- -------Q-- ---------- A--------- TGCGRDC--- ----LGSCC- ----------
SppGGP-1     -------TVV SNFQE----- ---------- ---------- ---------- ---------- -----AEE-- ---------- S--------- LGCGRSC--- ----LGKCC- ----------
SppGGP-2     -------TVV SNFQE----- ---------- ---------- ---------- ---------- -----AAE-- ---------- A--------- LGCGRNC--- ----LGKCC- ----------
TcGGP-1      -------TVL SNYQK----- ---------- --------DE AEE------- ---------- ----TARR-- ---------- S--------- GGCGKNC--- ----LRSCC- ----------
TcGGP-2      -------TVL SNYQE----- ---------- --------DA YEK------- ---------- -------Q-- ---------- E--------- VGCGRNC--- ----LGNCC- ----------
TpGGP        -------TVV SNYQK----- ---------- --------EE VGD------- ---------- ----APRT-- ---------- V--------- GGCGRNC--- ----LKACC- ----------
VvGGP-1      -------TVV SNYQK----- ---------- --------ED SDD------- ---------- ----GARQ-- ---------- V--------- GGCGRNC--- ----LKQCC- ----------
VvGGP-2      -------TVV SNYQE----- ---------- --------EA SE-------- ---------- ---------- ---------- S--------- LGCGRNC--- ----LGHCC- ----------
VcGGP        S-ASSDSTVA SCLDAYLPIY IF-------- --------ST SRKSIEKPLK LSRVDSFPTV PDLTDEPTCD S---PQFHGS SLPTY----- CPTGDDSFNS LP--INICSH VQQQFSSPYD
ZmGGP-E-1    -------TVV SNYQE----- ---------- --------DA ---------- ---------- -----DKP-- ---------- R--------- AGCGRNC--- ----LGDCC- ----------
ZmGGP-E-2    --AS-PPTAA SRADE----- ---------- --------GG GESFAVSTAV FAP----KSN RNLRGQRKVG NLVREPALSE T--------- PILGRHCRLF FV--LGKAL- ----------
ZmGGP-1      -------TVV SNYQE----- ---------- --------DA ---------- ---------- -----DKP-- ---------- R--------- AGCGRNC--- ----LGDCC- ----------
ZmGGP-2      --AS-PPTAA SRADE----- ---------- --------GG GESFYCCL-- -------RSE IQPKSQRKVG NLVREPALSE T--------- PILGRHCRLF FV--LGKAL- ----------
ZomGGP       -------TVL SNYQE----- ---------- --------DG KSE------- ---------- ---------- ---------- T--------- VGCGRSC--- ----LGKCC- ----------

             ....|....| ....|....| ....|....| ....|....| ....|....| ....|....| ....|....| ....|....| ....|....| ....|....| ....|....| ....|....|
                    370        380        390        400        410        420        430        440        450        460        470        480
AcGGP        IQGAKLPLYA FKRVKEVV-- ---------- ---------- ---------- ---------- ---------- ---------- ------GEK- ---------- ---------- ----------
AcGGP-R-1    IQGAKLPLYA FKRVNEVV-- ---------- ---------- ---------- ---------- ---------- ---------- ------GEK- ---------- ---------- ----------
AcGGP-R-2    LPVSKLPLYA FKKDEDNA-- ---------- ---------- ---------- ---------- ---------- ---------- ------IYKH ---------- --NGS----- ---------S
AdGGP        IQGAKLPLYA FKRVKEVV-- ---------- ---------- ---------- ---------- ---------- ---------- ------GEK- ---------- ---------- ----------
AeGGP        IQGAKLPLYA FKRVNEVV-- ---------- ---------- ---------- ---------- ---------- ---------- ------GEK- ---------- ---------- ----------
```

```
ArGGP        IQGAKLPLYA FKRVNEVV-- ---------- ---------- ---------- ---------- ---------- ---------- ------GEK- ---------- ---------- ----------
AmhGGP-1     IQGAKFPLYA FKRINKIE-- ---------- ---------- ---------- ---------- ---------- ---------- ------TEQ- ---------- ---------- ----------
AmhGGP-2     LLVSKLPLYT YKNEDEDM-- ---------- ---------- ---------- ---------- ---------- ---------- ------IEN- ---------- ---------- ----------
AncGGP-3     RQGAKLLLYC FSKIDEDL-- ---------- ---------- ---------- ---------- ---------- ---------- ---------- ---------- ---------- ----------
AncGGP-1     LPVSKLPLYA FKEDPRSL-- ---------- ---------- ---------- ---------- ---------- ---------- ------ISS- ---------- ---------- ----------
AncGGP-2     LSLSKLPLYA FKGDS----- ---------- ---------- ---------- ---------- ---------- ---------- ---------- ---------- ---------- ----------
AqcGGP       LPGAKLPLYT VRRMKNKI-- ---------- ---------- ---------- ---------- ---------- ---------- ------NEK- ---------- ---------- ----------
AhGGP-1      INGARLPLYA CKNMEKSG-- ---------- ---------- ---------- ---------- ---------- ---------- -----AVEK- ---------- ---------- ----------
AhGGP-2      INGARLPLYT CKNLDKSV-- ---------- ---------- ---------- ---------- ---------- ---------- ------GE-- ---------- ---------- ----------
AlGGP-1      INGARLPLYA CKNLAKSG-- ---------- ---------- ---------- ---------- ---------- ---------- -----AVEK- ---------- ---------- ----------
AlGGP-2      INGARLPLYT CKNLDTFV-- ---------- ---------- ---------- ---------- ---------- ---------- ------GE-- ---------- ---------- ----------
AtGGP-1      LNGARLPLYA CKNLVKSG-- ---------- ---------- ---------- ---------- ---------- ---------- -------EK- ---------- ---------- ----------
AtGGP-2      INGARLPLYT CKNLDKSV-- ---------- ---------- ---------- ---------- ---------- ---------- ------GE-- ---------- ---------- ----------
BosGGP-1     INGARLPLYA CKNLEKSN-- ---------- ---------- ---------- ---------- ---------- ---------- -------EK- ---------- ---------- ----------
BosGGP-2     ISGVRLPLYT CKNLDKSV-- ---------- ---------- ---------- ---------- ---------- ---------- ------GE-- ---------- ---------- ----------
BdGGP-1      LPVSELPVYA FKANPTKL-- ---------- ---------- ---------- ---------- ---------- ---------- ---------- ---------- ---------- ----------
BdGGP-2      LPASKLPLYA FKASNQNK-- ---------- ---------- ---------- ---------- ---------- ---------- ---------- ---------- ---------- ----------
BdGGP-3      ---------- --------M- ---------- ---------- ---------- ---------- ---------- ---------- ------GSKA ---------V DS-------- ----------
BsGGP-1      LPVSELPVYA FKANPTKL-- ---------- ---------- ---------- ---------- ---------- ---------- ---------- ---------- ---------- ----------
BsGGP-2      LPASKLPLYA FKASQKK--- ---------- ---------- ---------- ---------- ---------- ---------- ---------- ---------- ---------- ----------
BsGGP-3      ---------- --------M- ---------- ---------- ---------- ---------- ---------- ---------- ------GSKG ---------V DS-------- ----------
BoGGP-1      INGARLPLYA CKKLDKSG-- ---------- ---------- ---------- ---------- ---------- ---------- ---------- ---------- ---------- ----------
BoGGP-2      TVRARLPLYA CNKLGKSA-- ---------- ---------- ---------- ---------- ---------- ---------- ---------- ---------- ---------- ----------
BoGGP-3      INGERLPLYT CK-------- ---------- ---------- ---------- ---------- ---------- ---------- ---------- ---------- ---------- ----------
BrGGP-1      INGARLPLYA CKKLDKSG-- ---------- ---------- ---------- ---------- ---------- ---------- ---------- ---------- ---------- ----------
BrGGP-2      LNGARLPLYA CKKLENSG-- ---------- ---------- ---------- ---------- ---------- ---------- -----TGEK- ---------- ---------- ----------
BrGGP-3      VNGARLPLYV CKKVEDSG-- ---------- ---------- ---------- ---------- ---------- ---------- -------EK- ---------- ---------- ----------
BrGGP-4      INVERLPLYT CK-------- ---------- ---------- ---------- ---------- ---------- ---------- ---------- ---------- ---------- ----------
CgGGP-1      INGARLPLYA CKNLEKSR-- ---------- ---------- ---------- ---------- ---------- ---------- -------EK- ---------- ---------- ----------
CgGGP-2      INGARLPLYT CKNLDKSV-- ---------- ---------- ---------- ---------- ---------- ---------- ------KD-- ---------- ---------- ----------
CrGGP-1      INGARLPLYA CKNLEKSR-- ---------- ---------- ---------- ---------- ---------- ---------- -------EK- ---------- ---------- ----------
CrGGP-2      INGARLPLYT CNNLDKSV-- ---------- ---------- ---------- ---------- ---------- ---------- ------KD-- ---------- ---------- ----------
ChrGGP       VPDADLHGYS FNDLPLAISG -IDVGSGSGA SSPLASSAGV VPSMDEGADV DVDVDAVVTL AASSPHKAAG LHPRHPGHHQ APRRHASDVE ------FVRM ARRGGAGAAA AA--------
CcGGP-1      IQGAKLPLYA FKRVNKFK-- ---------- ---------- ---------- ---------- ---------- ---------- ------TEM- ---------- ---------- ----------
CcGGP-2      LPVSKLPLYQ ITKDKVDL-- ---------- ---------- ---------- ---------- ---------- ---------- ------TEE- ---------- ---------- ----------
CsGGP-1      IQGAKLPLYA FKRVNKFK-- ---------- ---------- ---------- ---------- ---------- ---------- ------TEM- ---------- ---------- ----------
CsGGP-2      LPVSKLPLYQ ITKDKVDL-- ---------- ---------- ---------- ---------- ---------- ---------- ------TEE- ---------- ---------- ----------
CosGGP       LSEGRVP--- ---------- ---------- ---------- ---------- ---------- ---------- ---------- ---------- ---------- ---------- ----------
CusGGP       IEGAKIPLYA FKKLNKIS-- ---------- ---------- ---------- ---------- ---------- ---------- ------GSK- ---------- ---------- ----------
DcGGP-1      LQGAKLPLYA FKRVNKCG-- ---------- ---------- ---------- ---------- ---------- ---------- ------NRG- ---------- ---------- ----------
DcGGP-2      LPGSKLPLYA FKKETAKE-- ---------- ---------- ---------- ---------- ---------- ---------- ------LAA- ---------- ---------- ----------
DsGGP        -QGARLPMYI YADHAPSKM- --QRIPSYSR WD-VG----- IEDADTSSPL CPCVDDASD- ---------- ---------- -----DSDQA YWTKPSAQAY SSDGAVPCMP SSPYLGDDPM
EgGGP-1      VQGAKFPLYA FKKEKEVP-- ---------- ---------- ---------- ---------- ---------- ---------- ------GDR- ---------- ---------- ----------
EgGGP-2      IPGAKLPLYA FKRVNFVA-- ---------- ---------- ---------- ---------- ---------- ---------- ------DEK- ---------- ---------- ----------
EgGGP-3      LP--DLPLYT FKGE--DC-- ---------- ---------- ---------- ---------- ---------- ---------- ------AEE- ---------- ---------- ----------
EgGGP-4      LPASDLPLYT FKVAREDC-- ---------- ---------- ---------- ---------- ---------- ---------- ------TEE- ---------- ---------- ----------
EgGGP-5      LPASDLPLYT FKGE--DC-- ---------- ---------- ---------- ---------- ---------- ---------- ------AEE- ---------- ---------- ----------
EsGGP-1      INGARLPLYA CKKLEKSG-- ---------- ---------- ---------- ---------- ---------- ---------- -----AGEK- ---------- ---------- ----------
EsGGP-2      INGARLPLYT CNNVDKSV-- ---------- ---------- ---------- ---------- ---------- ---------- ------GE-- ---------- ---------- ----------
FvGGP-1      ISGAKLPLYA FKKQNNSP-- ---------- ---------- ---------- ---------- ---------- ---------- ------GEK- ---------- ---------- ----------
FvGGP-2      ISGAKLPLYA FKKQNNSP-- ---------- ---------- ---------- ---------- ---------- ---------- ------GEK- ---------- ---------- ----------
FvGGP-3      LPGAKLPLYA FKKRENSP-- ---------- ---------- ---------- ---------- ---------- ---------- ------GDE- ---------- ---------- ----------
GmGGP-1      IPDAKLPLYG FKKANI---- ---------- ---------- ---------- ---------- ---------- ---------- ------GKD- ---------- ---------- ----------
GmGGP-2      LPDAKLPLYA FKKAN----- ---------- ---------- ---------- ---------- ---------- ---------- ------GKD- ---------- ---------- ----------
GmGGP-3      IQGARLPLYA FKKVDKVC-- ---------- ---------- ---------- ---------- ---------- ---------- ------GNE- ---------- ---------- ----------
GmGGP-4      IQDARLPLYA FKRGEKVG-- ---------- ---------- ---------- ---------- ---------- ---------- ------GKE- ---------- ---------- ----------
GnmGGP       VPGSKMPLYT FKRVKTHV-- ---------- ---------- ---------- ---------- ---------- ---------- ------QDK- ---------- ---------- ----------
GrGGP-1      IPGAKLPLYA FKKVN-ME-- ---------- ---------- ---------- ---------- ---------- ---------- ------REE- ---------- ---------- ----------
GrGGP-2      IPGAKLPLYA FRKLNNIE-- ---------- ---------- ---------- ---------- ---------- ---------- ------SEN- ---------- ---------- ----------
GrGGP-3      IPGAKLPLYA FKK----E-- ---------- ---------- ---------- ---------- ---------- ---------- ------SEK- ---------- ---------- ----------
KfGGP-1      IQAAKLPLYG YKWKNVSV-- ---------- ---------- ---------- ---------- ---------- ---------- ------KGT- ---------- ---------- ----------
KfGGP-2      IRAAKLPLYG YKWKSETE-- ---------- ---------- ---------- ---------- ---------- ---------- ------KGT- ---------- ---------- ----------
KlGGP-1      IQAAKLPLYG YKWKNVSV-- ---------- ---------- ---------- ---------- ---------- ---------- ------KGT- ---------- ---------- ----------
KlGGP-2      IRAAKLPLYG YKWKSETE-- ---------- ---------- ---------- ---------- ---------- ---------- ------KGT- ---------- ---------- ----------
KlGGP-3      IRAAKLPLYG YKWKSETE-- ---------- ---------- ---------- ---------- ---------- ---------- ------KGA- ---------- ---------- ----------
LuGGP        IPGAKLPLYT FKRVDVIP-- ---------- ---------- ---------- ---------- ---------- ---------- ------GDK- ---------- ---------- ----------
MdGGP        IPGAKLPLYA FKKRNVNN-- ---------- ---------- ---------- ---------- ---------- ---------- ------GDT- ---------- ---------- ----------
MeGGP-1      IQGAKLPLYA FKKLNKIV-- ---------- ---------- ---------- ---------- ---------- ---------- ------REK- ---------- ---------- ----------
MeGGP-2      IQGAKLPLYA FKKLNKIV-- ---------- ---------- ---------- ---------- ---------- ---------- ------CEK- ---------- ---------- ----------
MeGGP-3      LPGSSVPLYS FKIDRENS-- ---------- ---------- ---------- ---------- ---------- ---------- ------KEN- ---------- ---------- ----------
MpGGP        -LGASLPLYC YGRRDTTKV- -VTNV----- ---------- ---------- ---------- ---------- ---------- ------SENA ---------L SLD------- ----------
MtGGP-1      IQDAKLPLYA FKKIDKVT-- ---------- ---------- ---------- ---------- ---------- ---------- ------EKD- ---------- ---------- ----------
MtGGP-2      IQEAKLPLYG FKRGE----- ---------- ---------- ---------- ---------- ---------- ---------- ------AKD- ---------- ---------- ----------
MpGGP        RARRPLPLLT YAKHRQECE- -PKRVRSFGS FDDLR----- VPDISANGQG CVNMDAYA-- ---------- ---------- ------NDGG ---------F SDDGSLSTSC AATFGVNDGC
MsGGP        RAGKKLPTLT FAPESARDSR LMKKVRSFGS FDDLR----- VPDI------ ----DGYS-- ---------- ---------- ------NEE- ---------- ---------- ----------
MgGGP-1      LPGSKLPLYA FKKANKLV-- ---------- ---------- ---------- ---------- ---------- ---------- ------CEK- ---------- ---------- ----------
MgGGP-2      LPGAKLPLYA FKKAKDGV-- ---------- ---------- ---------- ---------- ---------- ---------- ------CEK- ---------- ---------- ----------
MaGGP-1      LPVSKLPLYA FKSDARPE-- ---------- ---------- ---------- ---------- ---------- ---------- ------I--- ---------- ---------- ----------
MaGGP-2      LPASKLPVYA FKNDPRPK-- ---------- ---------- ---------- ---------- ---------- ---------- ------A--- ---------- ---------- ----------
MaGGP-3      LAVSKLPLYA FESDARPK-- ---------- ---------- ---------- ---------- ---------- ---------- ------I--- ---------- ---------- ----------
MaGGP-4      WIGIRLPLYY FSKAEKDS-- ---------- ---------- ---------- ---------- ---------- ---------- ---------- ---------- ---------- ----------
OtGGP        LPVSKLALYA FKANP----- ---------- ---------- ---------- ---------- ---------- ---------- ---------- ---------- ---------- ----------
OsGGP-1      LPDSKLPLYA FKASPKKP-- ---------- ---------- ---------- ---------- ---------- ---------- ---------- ---------- ---------- ----------
OsGGP-2      ---------- FVAERKTGM- ---------- ---------- ---------- ---------- ---------- ---------- ------GNRS ---------F DL-------- ----------
OlGGP        ---------- ---------- ---------- ---------- ---------- ---------- ---------- ---------- ---------- ---------- ---------- ----------
PhGGP-1      LPVSKLPLYA FKANPAK--- ---------- ---------- ---------- ---------- ---------- ---------- ---------- ---------- ---------- ----------
PhGGP-2      ---------- --------M- ---------- ---------- ---------- ---------- ---------- ---------- ------DGKN ---------F GL-------- ----------
PvGGP-1      LPVSKLPLYA FKANPAK--- ---------- ---------- ---------- ---------- ---------- ---------- ---------- ---------- ---------- ----------
PvGGP-2      EPVSKLPLYA FKANPAK--- ---------- ---------- ---------- ---------- ---------- ---------- ---------- ---------- ---------- ----------
PvGGP-3      ---------- --------M- ---------- ---------- ---------- ---------- ---------- ---------- ------DGKS ---------V SL-------- ----------
PhvGGP-1     LPDAKLPLYA FKKVV----- ---------- ---------- ---------- ---------- ---------- ---------- ------NEK- ---------- ---------- ----------
PhvGGP-2     IQGARLPLYA CKRGDKIG-- ---------- ---------- ---------- ---------- ---------- ---------- ------GKE- ---------- ---------- ----------
PpGGP-1      IPGAKLPLYA FGVRQSSG-- ---------- ---------- ---------- ---------- ---------- ---------- ------SNK- ---------- ---------- ----------
PpGGP-2      IPGAKLPLYT FGVKKNSS-- ---------- ---------- ---------- ---------- ---------- ---------- ------LHK- ---------- ---------- ----------
PpGGP-3      IRGTKLPLYT FGLKGLLS-- ---------- ---------- ---------- ---------- ---------- ---------- ------SNK- ---------- ---------- ----------
PaGGP-1      VPGSKLPLYT FKRRHTIT-- ---------- ---------- ---------- ---------- ---------- ---------- ------GEK- ---------- ---------- ----------
PaGGP-2      VPGSKLPLYT FKRRLTIA-- ---------- ---------- ---------- ---------- ---------- ---------- ------GEE- ---------- ---------- ----------
PaGGP-3      VPGSKLPLYT FKRRHTIV-- ---------- ---------- ---------- ---------- ---------- ---------- ------GEK- ---------- ---------- ----------
PsGGP-1      VPGSKLPLYT FKRRHTIT-- ---------- ---------- ---------- ---------- ---------- ---------- ------GEK- ---------- ---------- ----------
PsGGP-2      VPGSKLPLYT FKRRHTIV-- ---------- ---------- ---------- ---------- ---------- ---------- ------GVE- ---------- ---------- ----------
PsGGP-3      VPGSKLPLYT FKRRHTIV-- ---------- ---------- ---------- ---------- ---------- ---------- ------GEK- ---------- ---------- ----------
PtGGP-1      VPGSKLPLYT FKRRYTIS-- ---------- ---------- ---------- ---------- ---------- ---------- ------GEK- ---------- ---------- ----------
PtGGP-2      LPGSKLPLYT FKRRHTIV-- ---------- ---------- ---------- ---------- ---------- ---------- ------GEK- ---------- ---------- ----------
PtGGP-3      VPGSKLPLYT FKRRITIA-- ---------- ---------- ---------- ---------- ---------- ---------- ------GEK- ---------- ---------- ----------
PtGGP-4      VPVSKLPLYT FRRRHTIV-- ---------- ---------- ---------- ---------- ---------- ---------- ------EEK- ---------- ---------- ----------
PtGGP-5      VPGSMLPLYT FKRRHTIV-- ---------- ---------- ---------- ---------- ---------- ---------- ------GEK- ---------- ---------- ----------
PotGGP-1     LQDACLPLYA FKKVDSIV-- ---------- ---------- ---------- ---------- ---------- ---------- ------SKKK ---------- ---------- ---------G
PotGGP-2     LQGARLPLYT FKKVDRII-- ---------- ---------- ---------- ---------- ---------- ---------- ------TEQK ---------- ---------- ----------
PotGGP-3     LPVSKLPLYA FKEDNSNL-- ---------- ---------- ---------- ---------- ---------- ---------- ------IGN- ---------- ---------- ----------
PrpGGP       IPGAKLPLYA FKKLTKID-- ---------- ---------- ---------- ---------- ---------- ---------- ------GDK- ---------- ---------- ----------
RcGGP-1      IQGAKLPLYA FKRVDKIV-- ---------- ---------- ---------- ---------- ---------- ---------- ------SEK- ---------- ---------- ----------
RcGGP-2      -MSAKLPLYA FKRLNKIV-- ---------- ---------- ---------- ---------- ---------- ---------- ------CEK- ---------- ---------- ----------
RcGGP-3      LPGSNLPLYS FKKDGENL-- ---------- ---------- ---------- ---------- ---------- ---------- ------LQN- ---------- ---------- ----------
SpGGP-1      LQGARLPLYT FKKVDRIA-- ---------- ---------- ---------- ---------- ---------- ---------- ------TD-- ---------- ---------- ----------
SpGGP-2      LQDARLPLYA FKKVNSIV-- ---------- ---------- ---------- ---------- ---------- ---------- ------SEKE ---------- ---------- ---------G
SpGGP-3      LPVSKLPLYA FKEDNSNL-- ---------- ---------- ---------- ---------- ---------- ---------- ------IGN- ---------- ---------- ----------
```

```
SmGGP-1      IPGARLPLYL FGNQELDEH- ---------- ---------- ---------- ---------- ---------- ---------- ---------- ---------- ---------- ----------
SmGGP-2      FPGARVPLYL YGEAQCMD-- ---------- ---------- ---------- ---------- ---------- ---------- ---------- ---------- ---------- ----------
SmGGP-3      LTGFDLPLFK YTKKMKTTE- -LQLL----- ---------- ---------- ---------- ---------- ---------- ------GDE- ---------- ---------- ----------
SiGGP-1      LPVSKLPLYA FKANPAK--- ---------- ---------- ---------- ---------- ---------- ---------- ---------- ---------- ---------- ----------
SiGGP-2      ---------- FVVKTKLAM- ---------- ---------- ---------- ---------- ---------- ---------- ------DVKS ---------F DL-------- ----------
SvGGP-1      LPVSKLPLYA FKANPAK--- ---------- ---------- ---------- ---------- ---------- ---------- ---------- ---------- ---------- ----------
SvGGP-2      ---------- FVVKTKLAM- ---------- ---------- ---------- ---------- ---------- ---------- ------DVKS ---------F DL-------- ----------
SlGGP-1      LPGSKLPLYG FKNLSYGK-- ---------- ---------- ---------- ---------- ---------- ---------- ---------- ---------- ---------- ----------
SlGGP-2      MPVSVLPLYA FKNDDNEP-- ---------- ---------- ---------- ---------- ---------- ---------- ------IEN- ---------- ---------- ----------
StGGP-1      LPGSKLPLYG FKNLSYGK-- ---------- ---------- ---------- ---------- ---------- ---------- ---------- ---------- ---------- ----------
StGGP-2      MPVSVLPLYA FKNDDNEP-- ---------- ---------- ---------- ---------- ---------- ---------- ------IEN- ---------- ---------- ----------
SbGGP-1      LPASKLPLYA FKLNPAK--- ---------- ---------- ---------- ---------- ---------- ---------- ---------- ---------- ---------- ----------
SbGGP-2      ---------- FFAETKLTM- ---------- ---------- ---------- ---------- ---------- ---------- ------DDTS ---------F DL-------- ----------
SfGGP-1      MPGAKLPLYT FGRRRSVDL- ---------- ---------- ---------- ---------- ---------- ---------- ------LKKK ---------M AV-------- ---------A
SfGGP-2      MPGAKLPLYT FRRRQSAN-- ---------- ---------- ---------- ---------- ---------- ---------- ------VKN- ---------- ---------- ----------
SppGGP-1     LPAAKLPLYA FKTEIQEA-- ---------- ---------- ---------- ---------- ---------- ---------- ------SAKG ---------- ---------- ----------
SppGGP-2     LPVSKLPLYS FKNEDKDE-- ---------- ---------- ---------- ---------- ---------- ---------- ------DPKG ---------- ---------- ----------
TcGGP-1      IPGAKLPLYA FKKVTNTQ-- ---------- ---------- ---------- ---------- ---------- ---------- ------SEK- ---------- ---------- ----------
TcGGP-2      IPASTLPLYA FQKEQDDS-- ---------- ---------- ---------- ---------- ---------- ---------- ------GEY- ---------- ---------- ----------
TpGGP        IQDAKLPLYA FKKINKVN-- ---------- ---------- ---------- ---------- ---------- ---------- ------GKD- ---------- ---------- ----------
VvGGP-1      IQGAKLPLYA YKKVKDVV-- ---------- ---------- ---------- ---------- ---------- ---------- ------NEK- ---------- ---------- ----------
VvGGP-2      LPVSKLPLYT FKKNVKDS-- ---------- ---------- ---------- ---------- ---------- ---------- ------SEA- ---------- ---------- ----------
VcGGP        VVGKGLPYTQ FGNEVLPSA- ---------- ---------- CSCADAGIGA IPLVDTKASL APGRAH---- ---------- -PRRHPSDVR ---------L SRPGSMGSSL A------DDC
ZmGGP-E-1    LPASKLPLYA FKLTPAK--- ---------- ---------- ---------- ---------- ---------- ---------- ---------- ---------- ---------- ----------
ZmGGP-E-2    SDSCIFILAM LSAATKLTM- ---------- ---------- ---------- ---------- ---------- ---------- ------DGTS ---------F DL-------- ----------
ZmGGP-1      LPASKLPLYA FKLSPAK--- ---------- ---------- ---------- ---------- ---------- ---------- ---------- ---------- ---------- ----------
ZmGGP-2      SDSCIFILAM LSAATKLTM- ---------- ---------- ---------- ---------- ---------- ---------- ------DGTS ---------F DL-------- ----------
ZomGGP       FPVSNFPLYE FKKDTQKH-- ---------- ---------- ---------- ---------- ---------- ---------- -------EK- ---------- ---------- ----------

             ....|....| ....|....| ....|....| ....|....| ....|....| ....|....| ....|....| ....|....| ....|....| ....|....| ....|....| ....|....|
                     490        500        510        520        530        540        550        560        570        580        590        600
AcGGP        -G-LLAV--- D--------- ---------- ---------- ---------- ----DE-EAP VAFLDSLL-- ---LGEWEDR VQRGLFRYDV TACETKVIPG EYGFIAQLNE GRHLKKRPTE
AcGGP-R-1    -G-VLAL--- D--------- ---------- ---------- ---------- ----NE-EAP VAFLDSLL-- ---LGEWEDR VQRGLFRYDV TACETKVIPG EYGFIAQLNE GRHLKKRPTE
AcGGP-R-2    PG-EARP--- V--------- ---------- ---------- ---------- ----AE-VRP IGLLNNLL-- ---LGQWEER MARGLFRYDV TACETKIIPG NYGFIAQLNE GRHLKKRPTE
AdGGP        -G-LLAV--- D--------- ---------- ---------- ---------- ----DE-EAP VAFLDSLL-- ---LGEWEDR VQRGLFRYDV TACETKVIPG EYGFIAQLNE GRHLKKRPTE
AeGGP        -G-VLAL--- D--------- ---------- ---------- ---------- ----NE-EAP VAFLDSLL-- ---LGEWEDR VQRGLFRYDV TACETKVIPG EYGFIAQLNE GRHLKKRPTE
ArGGP        -G-VLAL--- D--------- ---------- ---------- ---------- ----NE-EAP VAFLDSLL-- ---LGEWEDR VQRGLFRYDV TACETKVIPG EYGFIAQLNE GRHLKKRPTE
AmhGGP-1     -A-LTVD--- E--------- ---------- ---------- ---------- ----PR-KSP VAFLDSLL-- ---LGEWEDR VERGLFRYDV TACETKVIPG DYGFVAQLNE GRHLKKRPTE
AmhGGP-2     -G-MTYS--- V--------- ---------- ---------- ---------- ----DQ-MPT ISFLHDLL-- ---LGQWEDR MRKGFFRYDV TQCETKIIPG RYGFIAQLNE GRLLKKRPTE
AncGGP       ---CLPV--- P--------- ---------- ---------- ---------- ----KV-QPR VSFLDR-L-- ---LEEWDDR KVRGLFHHDI AACETKVLPG KHNFIIVLIE GRDLKKRPTE
AncGGP-1     -SSSSPG--- E--------- ---------- ---------- ---------- ----DEAPPA DFFLHTLL-- ---LGQWEDR MSRGLFRYDV TACETKVIPG EHGFIAQLNE GRHLKKRPTE
AncGGP-2     ---EISP--- N--------- ---------- ---------- ---------- ----ST-SEG FFFLNSLL-- ---LTQWDER MSRGLFRYDV TACEAKVVPG KCGFVAQLNE GRHRKKRPTE
AqcGGP       -D-LV----- ---------- ---------- ---------- ---------- ----GE-KQE ATFLDTLL-- ---LGEWEDR AQRGLFRYDV TACETKVIPG EYGFIAQLNE GRHLKKRPTE
AhGGP-1      -L-VISN--- E--------- ---------- ---------- ---------- ----AK-EPP VAFLESLV-- ---LGEWEDR FQRGLFRYDV TACETKVIPG KYGFVAQLNE GRHLKKRPTE
AhGGP-2      ---------- ---------- ---------- ---------- ---------- ----KL-ESP VTFLESLV-- ---LGEWEDR FQRGLFRYDV TACETKVIPG KYGFIAQLNE GRHLKKRPTE
AlGGP-1      -L-VISN--- E--------- ---------- ---------- ---------- ----AK-EPP VAFLESLV-- ---LGEWEDR FQRGLFRYDV TACETKVIPG KYGFVAQLNE GRHLKKRPTE
AlGGP-2      ---------- ---------- ---------- ---------- ---------- ----KL-ESP VMFLESLV-- ---LGEWEDR FQRGLFRYDV TACETKVIPG KYGFIAQLNE GRLLKKRPTE
AtGGP-1      -L-VISH--- E--------- ---------- ---------- ---------- ----AI-EPP VAFLESLV-- ---LGEWEDR FQRGLFRYDV TACETKVIPG KYGFVAQLNE GRHLKKRPTE
AtGGP-2      ---------- ---------- ---------- ---------- ---------- ----NT-ESP VTFLESLV-- ---IGEWEDR FQRGLFRYDV TACETKVIPG KYGFIAQLNE GRHLKKRPTE
BosGGP-1     -L-VISH--- E--------- ---------- ---------- ---------- ----AK-EAP VDFLESLV-- ---LGEWEDR FQRGLFRYDV TACETKVIPG KYGFVAQLNE GRHLKKRPTE
BosGGP-2     ---------- ---------- ---------- ---------- ---------- ----KL-ESS VTFLESLV-- ---LGEWEDR FQRGLFRYDV TACETKVIPG KYGFIAQLNE GRHLKKRPTE
BdGGP-1      ----PAS--- Q--------- ---------- ---------- ---------- ----EH-AVP SNVFIDLL-- ---LGQWEDR MTQGLFRYDV TACETKVIPG NLGFVAQLNE GRHLKKRPTE
BdGGP-2      ----FAQ--- E--------- ---------- ---------- ---------- ----DG-LPT DFFLNSLL-- ---LGQWEDR MAQGLFRYDV TACETKVIPG ELGFVAQLNE GRHLKKRPTE
BdGGP-3      -TYSFAVEDR E--------- ---------- ---------- ---------- ----EH-DPN LSPFLRKL-- ---FKQWDNC KARGLFHHDI TACETKVLPG EHNFVATLIE GRDQKKRPTE
BsGGP-1      -----SS--- Q--------- ---------- ---------- ---------- ----EH-AVP STFFIDLL-- ---LGQWEDR MTQGLFRYDV TACETKVIPG NLGFVAQLNE GRHLKKRPTE
BsGGP-2      ----AAH--- E--------- ---------- ---------- ---------- ----DG-LPT GFFLNSVL-- ---LGQWEDR MARGLFRYDV TACETKVIPG ELGFVAQLNE GRHLKKRPTE
BsGGP-3      -TYSFAV--- E--------- ---------- ---------- ---------- ----EH-D-N LSPFLRKL-- ---FKEWDDC KARGLFHHDI TACETKVLPG EHNFVATLIE GRDQKKRPTE
BoGGP-1      ---------- ---------- ---------- ---------- ---------- ----AG-EKP VAFLESLV-- ---LGEWEDR FQRGLFRYDV TACETKVIPG KYGFVAQLNE GRHLKKRPTE
BoGGP-2      -E-VINH--- E--------- ---------- ---------- ---------- ----AR-EPP VAFLESLV-- ---LGEWEDR YQRGLFRYDL TAC---VIPG KYGFIAQLNE GRHL-KRPTE
BoGGP-3      ---------- ---------- ---------- ---------- ---------- ----PA-EEN VTFLESVL-- ---LGEWEDR FQRGLFRYDV TACETKVIPG KYGFIAQLNE ARHLKKRPTE
BrGGP-1      ---------- ---------- ---------- ---------- ---------- ----VG-EKP VAFLESLV-- ---LGEWEDR FQRGLFRYDV TACETKVIPG KYGFVAQLNE GRHLKKRPTE
BrGGP-2      -V-VISH--- E--------- ---------- ---------- ---------- ----AR-EPP VAFLESLV-- ---LREWEDR FQRGLFRYDV TACETKVIPG KYGFVAQLNE GRHLKKRPTE
BrGGP-3      ---------- ---------- ---------- ---------- ---------- ----AR-EPS VAFLESLV-- ---LGEWEDR YQRGLFRYDL TACVTKVIPG KYGFIAQLNE GRHL-KRPTE
BrGGP-4      ---------- ---------- ---------- ---------- ---------- ----PA-EEN VTFLESLL-- ---LGEWEDR FQRGLFRYDV TACETKVLPG KYGFIAQLNE GRHLKKRPTE
CgGGP-1      -L-VISH--- E--------- ---------- ---------- ---------- ----AK-EPP VAFLESLV-- ---LGEWEDR FQRGLFRYDV TACETKVIPG KYGFVAQLNE GRHLKKRPTE
CgGGP-2      ---------- ---------- ---------- ---------- ---------- ----KS-ESP VTFLESVV-- ---LGEWEDR FQRGLFRYDV TACETKVIPG KCGFIAQLNE GRHLKKRPTE
CrGGP-1      -L-VISH--- E--------- ---------- ---------- ---------- ----AK-EPP VAFLESLV-- ---LGEWEDR FQRGLFRYDV TACETKVIPG KYGFVAQLNE GRHLKKRPTE
CrGGP-2      ---------- ---------- ---------- ---------- ---------- ----KS-ESP VTFLESVV-- ---LGEWEDR FQRGLFRYDV TACETKVIPG KCGFIAQLNE GRHLKKRPTE
ChrGGP       TAVSAAK--- AAAD------ ---------- -------IGG ---------- ----AE-VPG RSLLEGVV-- ---MALWEDR ADRGMFRYDV SQCETRVLPG PAGFVAQLNE GRATKKRPTE
CcGGP-1      -G-VHGH--- E--------- ---------- -------VR- ---------- ----EP-EPP VAFLDSLV-- ---LGEWEDR VQRGLFRYDV TACETRVIPG QYGFIAQLNE GRHLKKRPTE
CcGGP-2      -A-------- S--------- ---------- ---------- ---------- ----GE-EPR MSFLHDLL-- ---LGQWEDR MNRGLFRYDV TSCETKIIPG EYGFVAQLNE GRHLKKRPTE
CsGGP-1      -G-VHGH--- E--------- ---------- -------VR- ---------- ----EP-EPP VAFLDSLV-- ---LGEWEDR VQRGLFRYDV TACETRVIPG QYGFIAQLNE GRHLKKRPTE
CsGGP-2      -A-------- S--------- ---------- ---------- ---------- ----GE-EPR MSFLHDLL-- ---LGQWEDR MNRGLFRYDV TSCETKIIPG EYGFIAQLNE GRHLKKRATE
CosGGP       ----SAP--- S--------- ---------- ---------- ---------- ----SD-SGA TSLLDTVL-- ---LAEWEDR AEQGLFRYDV TACPTKVVPG AYGFVAQFNE GRGSKKRPTE
CusGGP       -GLCCEY--- E--------- ---------- ---------- ---------- ----NS-VPP VAFLDSLL-- ---LGEWEDR MQRGLFRYDV TACETKVIPG KYGFIAQLNE GRHLKKRPTE
DcGGP-1      -G-SSAE--- E--------- ---------- ---------- ---------- ----YK-EPP VAFLDSLI-- ---IGEWEDR VQKGLFRYDV TACETKVIPG NYGFIAQLNE GRHLKKRPTE
DcGGP-2      -E-EIKQ--- Q--------- ---------- ---------- ---------- ----PQ-QPP VAFLESLL-- ---LGEWEDR MQKGLFRYDV TACQTKVIPG DHGFIAQLNE GRHLKKRPTE
DsGGP        SS-SVTN--- SAEL------ ---------- ---------- ---------- ----GT-VTG RSLLDTVL-- ---LALWEDC AEKGLFRYDV TACATKVVPG MFGFIAQLNE GRASNKRPTE
EgGGP-1      -A-LLGH--- V--------- ---------- ---------- ---------- ----ER-EPP VAFLDSLL-- ---LGEWEDR MQRGLFRYDV TACETKVIPG ECGFIAQLNE GRHLKKRPTE
EgGGP-2      -A-GIVG--- E--------- ---------- ---------- ---------- ----NA-EPP VAFLDSLL-- ---LGEWEDR MQRGLFRYDV TACETKIIPG GYGFIAQLNE GRHLKKRPTE
EgGGP-3      ----SIF--- Y--------- ---------- ---------- ---------- ----QK-PPE TSFLLNLL-- ---LGQWKNR MNRGLFRYDV TTCKTKVIPS DYGFIVQLNE GRHLKKRPTE
EgGGP-4      ----SIF--- Y--------- ---------- ---------- ---------- ----QE-PPE TSFLLNLL-- ---LGQWENR MNQGLFHYDV TTCKTKVIPG DYGFIVQLNE GRHLKKRPTE
EgGGP-5      ----SIF--- Y--------- ---------- ---------- ---------- ----QK-PPE TSFLLNLL-- ---LGQWKNR MNRA------ ---------- --------ER GRHLKKRPTE
EsGGP-1      -L-VISH--- E--------- ---------- ---------- ---------- ----AR-EPP VAFLESLV-- ---LGEWEDR FQRGLFRYDV TACETKVIPG KYGFVAQLNE GRHLKKRPTE
EsGGP-2      ---------- ---------- ---------- ---------- ---------- ----KA-ESP VAFLDSLV-- ---LGEWEDR FQRGLFRYDV TACETKVIPG KYGFIAQLNE GRHLKKRPTE
FvGGP-1      -G-FSGH--- E--------- ---------- ---------- ---------- ----KQ-DAP VAFLDSLV-- ---LGEWEDR MQKGLFRYDV TACETKVIPG QFGFIAQLNE GRHLKKRPTE
FvGGP-2      -G-FSGH--- E--------- ---------- ---------- ---------- ----KQ-DAP VAFLDSLV-- ---LGEWEDR MLKGLFRYDV TACETKVIPG QFGFIAQLNE GRHLKKRPTE
FvGGP-3      ---------- K--------- ---------- ---------- ---------- ----KQ-EAP VAFLDSLV-- ---LGEWDDR MKKGLFRYDV TACETKMIPG QFGFIAQLNE GRHLKKRPTE
GmGGP-1      -L-ALHA--- C--------- ---------- ---------- ---------- ----AG-EPP LAFLDSLL-- ---LGEWEDR MQRGLFRYDV TACETKVIPG EYGFVAQLNE GRHLKKRPTE
GmGGP-2      -L-ALHA--- C--------- ---------- ---------- ---------- ----AG-EPP LTFLDSLL-- ---LGEWEDR MQRGLFRYDV TACETKVIPG EYGFVAQLNE GRHLKKRPTE
GmGGP-3      -L-PLLG--- C--------- ---------- ---------- ---------- ----KE-QHP VAFLDSLV-- ---LGEWEDR MQRGLFRYDV TACETKVIPG EYGFIAQLNE GRHLKKRPTE
GmGGP-4      -L-PLLG--- C--------- ---------- ---------- ---------- ----K--KHP VDFLDSLV-- ---LGEWEDR MQRGLFRYDV TTCETKVIPG KYGFIAQLNE GRHLKKRPTE
GnmGGP       ---SSSG--- ---------- ---------- ---------- ---------- ----NE-EGA MSFLNDLL-- ---LGQWEDR MQKGLFRYDV TACETKVIPG NLGFIAQLNE GRHLKKRPTE
GrGGP-1      -D-VLKM--- V--------- ---------- ---------- ---------- ----NT-EPP VAFLDSLV-- ---LGEWEDR MQRGLFRYDV TACETKVIPG EYGFIAQLNE GRHLKKRPTE
GrGGP-2      -V-VLGI--- E--------- ---------- ---------- ---------- ----NK-DPP VAFLDSLI-- ---LGEWEDR MQRGLFRYDV TACETKVIPG EYGFIAQLNE GRHLKKRPTE
GrGGP-3      -D-AFES--- E--------- ---------- ---------- ---------- ----NK-EPP VAFLDSLV-- ---LGEWEDR MQRGLFRYDV TACETKVIPG EYGFIAQLNE GRHLKKRPTE
KfGGP-1      -D-SSNC--- V--------- ---------- ---------- ---------- ----SK-GLP VSFLDSLV-- ---LGEWEER MQRGLFRYDV TACETKVIPG EYGFIAQLNE GRHLQKRPTE
KfGGP-2      -D-SLSC--- V--------- ---------- ---------- ---------- ----SK-GLP GNFLDSLV-- ---LGEWEER MQRGLFRYDV TACETKVIPG EYGFIAQLNE GRHLQKRPTE
KlGGP-1      -D-SSNC--- V--------- ---------- ---------- ---------- ----SK-GLP VSFLDSLV-- ---LGEWEER MQRGLFRYDV TACETKVIPG EYGFIAQLNE GRHLQKRPTE
KlGGP-2      -D-SLSC--- V--------- ---------- ---------- ---------- ----SK-GLP GNFLDSLV-- ---LGEWEER MQRGLFRYDV TACETKVIPG EYGFIAQLNE GRHLQKRPTE
KlGGP-3      -D-SLSC--- V--------- ---------- ---------- ---------- ----SK-GLP GNFLDSLV-- ---LGEWEER MQRGLFRYDV TACETKVIPG EYGFIAQLNE GRHLQKRPTE
LuGGP        -C-ALVH--- D--------- ---------- ---------- ---------- ----DN-PAP VAFLDSLL-- ---LGQWEDR MQRGLFRYDV TACETKVIPG RYGFVAQLNE GRHLKKRPTE
MdGGP        -G-VPGH--- D--------- ---------- ---------- ---------- ----KR-EPP VAFLDSLL-- ---LGEWEDR MQRGLFRYDV TACETKVIPG QYGFIAQLNE GRHLKKRPTE
MeGGP-1      -E-VLEH--- E--------- ---------- ---------- ---------- ----NN-EPP VAFLETLL-- ---LGEWEDR MQRGLFRYDV TACETKVIPG QYGFIAQLNE GRHLKKRPTE
MeGGP-2      -E-VRGQ--- E--------- ---------- ---------- ---------- ----NN-EPP VAFLESLL-- ---LGEWEDR VQRGLFRYDV TACETKVIPG QYGFIAQLNE GRHLKKRPTE
MeGGP-3      -G-EVNS--- A--------- ---------- ---------- ---------- ----KE-QPQ VCFLHNLL-- ---LGQWEDR MSRGLFRYDV TSCATRIIPG RWGFIAQLNE GRHLKKRPTE
MpGGP        SN-SASN--- N--------- ---------- ---------- ---------- ----QP-VED ASFLDSLL-- ---LSQWEDR MEKGLFRYDV TACETKVLPG VCGFIAQLNE GRHLKKRPTE
MtGGP-1      -L-AIHE--- C--------- ---------- ---------- ---------- ----QE-ELP MAFLDSLV-- ---LGEWEDR MQRGLFRYDV TACETKVIPG ECGFIAQLNE GRHLKKRPTE
MtGGP-2      ---LSPI--- A--------- ---------- ---------- ---------- ----CK-ERP VAFLDSII-- ---LGQWEDR MQRGLFRYDV TACETKVIPG EYGFIAQLNE GRHLKKRPTE
MpGGP        GG-SDGGG-- SDAG------ ---------- -------GGG GG----GGGG GSQSGY-VQD VSPFDRIL-- ---LAAWEDR FAAGLFRYDV TACDTKIIPG RIGFVAQYNE GRATKKRPTE
MsGGP        TG-SYNSD-- SDSF------ ---------- -------SGG ---------- ----GY-VQD VSPFDRIL-- ---LAAWEDR FAAGLFRYDV TACKTKVVPG GYGFVAQFNE GRATKKRPTE
MgGGP-1      -G-SFDS--- V--------- ---------- ---------- ---------- ----NK-EPP VEFLDALL-- ---LGEWEDR VQRGLFRYDV TACETKVIPG KYGFIAQLNE GRHLKKRPTE
MgGGP-2      -----VS--- E--------- ---------- ---------- ---------- ----NT-EPP VDFLDSLV-- ---VAGWEDR MQRGLFRYDV TACDTKVIAG NYGFIAQLNE GRHLKKRPTE
MaGGP-1      ---SSSG--- V--------- ---------- ---------- ---------- ----DE-PPS DFFLNSLL-- ---LGGWEDR MSRGLFRYDV TACETKVIPG EYGFVAQLNE GRHLKKRPTE
MaGGP-2      ---SSVG--- T--------- ---------- ---------- ---------- ----DE-LPP DFFLNTLL-- ---LGQWEDR MNRGLFRYDV TACETKVIPG DHGFIAQLNE GRHLKKRPTE
MaGGP-3      ---SSPG--- D--------- ---------- ---------- ---------- ----DE-PPS DFFLNTLL-- ---LGQWEDW MSRGLFRYDV MACETKVIPG EYGFVAQLNE GRHLKKRPTE
```

```
MaGGP-4     ---GCSL--- ---------- ---------- ---------- ---------- ----EK-IEA QSTFLDRL-- ---LKEWEDR KVRGLFHHDV NAYETKILSG DYGFILQLIE GRDLKKRPTE
OtGGP       ---------- Q--------- ---------- ---------- ---------- ----AD-AAQ TKPLVNLL-- ---LTEWENR LARGLFRYDV TACETKVIPG NLGFVAQLNE GRHLKKRPTE
OsGGP-1     -----SS--- Q--------- ---------- ---------- ---------- ----ED-ASN DEFFVNLL-- ---LGLWEDR MARGLFRYDV TACETKVIPG NLGFVAQLNE GRHLKKRPTE
OsGGP-2     -SCSLRKE-- E--------- ---------- ---------- ---------- ----EN-GTN LSPFVRKL-- ---FKEWDDR KARGLFHHDI SSCETKVLPG EHNFVATLIE GRDQKKRPTQ
OlGGP       ---------- ---------- ---------- ---------- ---------- ---------- ----DRIL-- ---ISAWEDR FAGGLFRYDV TAVSTKVIDG KKKYVAQFNI GRATNKRPTE
PhGGP-1     -----PS--- Q--------- ---------- ---------- ---------- ----ED-AAS TKSLVNIL-- ---LTEWEDR MARGLFRYDV TACETKVIPG NLGFVAQLNE GRHLKKRPTE
PhGGP-2     -SCSLGM--- E--------- ---------- ---------- ---------- ----DD-SLN LSRFLDKL-- ---FREWEDR KARGLFHHDI SCCETKVLPG ERNFVATLIE GRDQKKRPTE
PvGGP-1     -----PA--- Q--------- ---------- ---------- ---------- ----ED-AAS TKSLVNIL-- ---LAEWEDR MARGLFRYDV TACETKVIPG NLGFVAQLNE GRHLKKRPTE
PvGGP-2     -----PA--- Q--------- ---------- ---------- ---------- ----ED---- -KSLVNIL-- ---LAEWEDR MARGLFRYDV TACETKVIPG NLGFVAQLNE GRHLKKRPTE
PvGGP-3     -SCSFGM--- E--------- ---------- ---------- ---------- ----DD-SLN SSRFLDKL-- ---FREWEDR KARGLFHHDI SCCETK---- ---------- ----------
PhvGGP-1    -D-LALQ--- E--------- ---------- ---------- ---------- ----CG-EPP VAFLDSLL-- ---LGEWEDR MQKGLFRYDV TACETKVIPG EYGFVAQLNE GRHLKKRPTE
PhvGGP-2    -L-PLLG--- C--------- ---------- ---------- ---------- ----KE-HYP VAFLDSLI-- ---LGEWEER MQRGLFRYDV TACETKVIPG EYGFIAQLNE GRHLKKRPTE
PpGGP-1     -R-AHTG--- SDDH-CVPDS VKES------ --------D- ---------- ----VS-PQE NYFLDELL-- ---LAQWEDR MARGLFRYDV TACETKMLPG DCGFIAQLNE GRHLQKRPTE
PpGGP-2     -G-VHTN--- NGDH-CIPGG VGKPNGYG-- -------ID- ---------- ----DS-QQE NYFLDELL-- ---LAQWEDR MVRGLFRYDV TACETKMLPG EYGFIAQLNE GRHSQKRPTE
PpGGP-3     -S-VHDI--- DESH-CVPDD VGCH------ --------A- ---------- ----NP-PQE NYFLDE-L-- ---LAQWEDR MERGFFRYDV TACETKMLPG EYGFIAQLNE GRHSQKRPTE
PaGGP-1     ---YADK--- D--------- ---------- ---------- ---------- ----AE-VAN IPFLDSVL-- ---LGEWEER MQRGLFRYDV TTCETKVIPG NYGFIAQLNE GRHLKKRPTE
PaGGP-2     ---HAVK--- D--------- ---------- ---------- ---------- ----AK-VED ITLLDTVL-- ---LGQWEER MQRGLFRYDV TTCETKVIPG NYGFIAQLNE GRHLKKRPTE
PaGGP-3     ---HVDN--- N--------- ---------- ---------- ---------- ----VK-LAD IPSLDSLL-- ---LGLWEER MQRGLFRYDV TICETKVIPG NYGFIAQLNE GRHLKKRPTE
PsGGP-1     ---YADK--- D--------- ---------- ---------- ---------- ----AE-VAN IPFLDSVL-- ---LGEWEER MQRGLFRYDV TTCETKVIPG NYGFIAQLNE GRHLKKRPTE
PsGGP-2     ---HVDK--- D--------- ---------- ---------- ---------- ----VE-MAD TPFLDTLL-- ---IGQWEER MQRGLFRYDV TTCETKVIPG NYGFIAQLNE GRHLKKRPTE
PsGGP-3     ---HVDN--- D--------- ---------- ---------- ---------- ----VK-LAD IPSLDSLL-- ---LGLWEER MQRGLFRYDV TICETKVIPG NYGFIAQLNE GRHLKKRPTE
PtGGP-1     ---DADK--- D--------- ---------- ---------- ---------- ----AE-VAH ISFLDSVL-- ---LGEWEER MQRGLFRYDV TTCETKVIPG NYGFIAQLNE GRHLKKRPTE
PtGGP-2     ---HVDN--- D--------- ---------- ---------- ---------- ----VK-LTD VPSLDSLL-- ---LGLWEER MQRGLFRYDV TICETKVIPG NYGFIAQLNE GRHLKKRPTE
PtGGP-3     ---DAVK--- D--------- ---------- ---------- ---------- ----SK-VED ITLLDAVL-- ---VGQWEER MQRGLFRYDV TTCETKVIPG KYGFIAQLNE GRHLKKRPTE
PtGGP-4     ---HFDK--- D--------- ---------- ---------- ---------- ----VE-MAV TPFLDSLL-- ---IGQWEER MQRGLFRYDV TTCETKVIPG NYGFIAQLNE GRHLKKRPTE
PtGGP-5     ---HVDN--- D--------- ---------- ---------- ---------- ----VK-LTD VPSLDSLL-- ---LGLWEER MQRGLFRNTV TNSETKVIPG NYGFIAQLNE GRHLKKRPTE
PotGGP-1    VG-VFEY--- D--------- ---------- ---------- ---------- ----KG-EPP VAFLDSLL-- ---LGEWEDR VQRGLFRYDV TACETKVIPG QHGFIAQLNE GRHLKKRPTE
PotGGP-2    -D-VFEH--- D--------- ---------- ---------- ---------- ----KS-EPP VAFLNSLL-- ---LGEWEDR MQRGLFRYDV TTCETKVIPG RNGFIAQLNE GRHLKKRPTE
PotGGP-3    -S-VEKS--- S--------- ---------- ---------- ---------- ----EE-QPH MCFLHNLL-- ---LGQWEDR MSRGLFRYDV TACDTKIIPG RYGFIAQLNK GRHLKKRPTE
PrpGGP      -E-LPGS--- E--------- ---------- ---------- ---------- ----KR-EPP VDFLDSLL-- ---LGEWEDR MQRGLFRYDV TACETKVIPG QFGFIAQLNE GRHLKKRPTE
RcGGP-1     -E-VIEH--- E--------- ---------- ---------- ---------- ----NT-EPP VAFLDSLL-- ---LGEWEER VQRGLFRYDV TACETKVIPG QYGFIAQLNE GRHLKKRPTE
RcGGP-2     -E-EIEH--- E--------- ---------- ---------- ---------- ----NI-EPP VAFLDSLL-- ---LGEWEER MQRGLFRYDV TACETKVIPG QYGFIAQLNE GRHLKKRPTE
RcGGP-3     -G-EMKF--- S--------- ---------- ---------- ---------- ----KE-QPQ ICFLHNLL-- ---LGQWEDR MCRGLLRYDV TACETRIIPG KYGFIAQLNE GRHLKKRPTE
SpGGP-1     ---VPEH--- G--------- ---------- ---------- ---------- ----KS-EPP VSFLNSLL-- ---LGEWEER MQRGLFRYDV TTCETKVIPG SHGFIAQLNE GRHLKKRPTE
SpGGP-2     VG-VSED--- D--------- ---------- ---------- ---------- ----KG-ETT VAFLDSLL-- ---LGEWEER MQRGLFRYDV TACETKVIPG QYGFIAQLNE GRHLKKRPTE
SpGGP-3     -S-VEKS--- S--------- ---------- ---------- ---------- ----ED-QPQ ICFLHNLL-- ---LGQWEDR MSRGLFRYDV TACDTKIIPG RYGFIAQLNK GRHLKKRPTE
SmGGP-1     ---GEIQ--- S--------- ---------- ---------- ---------- ----KE-LGQ NSFLDSAI-- ---LGQWADK QAEGLFRYDV TACATKVLPG KYGFIAQLNE GRHLKKRPTE
SmGGP-2     ---------- ---------- ---------- ---------- ---------- ----TK-GET IDFLHSFI-- ---LAPWMEK QKQGLFRYDV TSCETKILSG DCGFIAQLNE GRHSKKRPTE
SmGGP-3     ---EVPS--- S--------- ---------- ---------- ---------- ----PP-TIE RSFLGTLL-- ---LSKWEER ASQGLFRYDV TACESRVLSG NYGFIAQLNE GRHLKKRLTE
SiGGP-1     -----PS--- Q--------- ---------- ---------- ---------- ----ED-ASS TKSLVNIL-- ---LTEWEDR MARGLFRYDV TACETKVIPG NLGFVAQLNE GRHLKKRPTE
SiGGP-2     -GCSLGM--- E--------- ---------- ---------- ---------- ----ND-SLS LSPFLNKL-- ---FREWEDR KARGLFHHDI SSCETKVLPG QHNFVATLIE GRDQKKRPTE
SvGGP-1     -----PS--- Q--------- ---------- ---------- ---------- ----ED-ASS TKSLVNIL-- ---LTEWEDR MARGLFRYDV TACETKVIPG NLGFVAQLNE GRHLKKRPTE
SvGGP-2     -GCSLGM--- E--------- ---------- ---------- ---------- ----ND-SLS LSPFLNKL-- ---FREWEDR KARGLFHHDI SSCETKVLPG QHNFVATLIE GRDQKKRPTE
SlGGP-1     ---SVAD--- E--------- ---------- ---------- ---------- ----TK-ESP IDFLESLV-- ---LGEWEDR QQKGLFRYDV TACETKVIPG EYGFVAQLNE GRHLKKRPTE
SlGGP-2     -D-VQTL--- P--------- ---------- ---------- ---------- ----EE-ECQ MSFLNDLL-- ---LGLWEER MSQGLFRYDV TTCETKVIPG RCGFIAQLNE GRHLKKRPTE
StGGP-1     -S-VAAD--- E--------- ---------- ---------- ---------- ----TK-ESP IDFLESLV-- ---LGEWEDR QQKGLFRYDV TACETKVIPG EYGFVAQLNE GRHLKKRPTE
StGGP-2     -D-IQTL--- P--------- ---------- ---------- ---------- ----EE-ECQ MSFLNNLL-- ---LGLWEER MSQGLFRYDV TTCETKVIPG RCGFIAQLNE GRHLKKRPTE
SbGGP-1     -----RS--- Q--------- ---------- ---------- ---------- ----EG-AAS TKLLVDIL-- ---LSEWEDR MARGLFRYDV TACETKVIPG NLGFVAQLNE GRHLKKRPTE
SbGGP-2     ------T--- E--------- ---------- ---------- ---------- ----HE-GSN LSPFLHRL-- ---FNEWDDR KTRGLFHHDI SSCETKVLPG EHNFVATLIE GRDQKKRPTE
SfGGP-1     CS-TNGG--- GDGHDTLLNG FPVRSSSEEH DNMSQGLLAG ---------- ----DS-DGD NGFLDSLL-- ---LAQWEDR MDKGLFRYDV TACETMVLPG EHGFIAQLNE GRHSKKRPTE
SfGGP-2     -N-AYSN--- GDSH-KVANG DTALHNYELH E-LSQGLVE- ---------- ----NS-EAA NGFLDSLL-- ---LAQWEDR MERGLFRYDV TACETMVLPG EHGFIAQLNE GRHSKKRPTE
SppGGP-1    KS-LPAA--- P--------- ---------- ---------- ---------- ----DE-DSP VDFLNNLV-- ---LGQWEDR MSRGLFRYDV TACETKVIPG EQGFIAQLNE GRHLKKRPTE
SppGGP-2    KK-ALSI--- P--------- ---------- ---------- ---------- ----DE-DPS IDFLNTLV-- ---LGQWEDR LSKGLFRYDV TACESKVIEG QHGFIAQLNE GRHLKKRPTE
TcGGP-1     -G-VRGN--- E--------- ---------- ---------- ---------- ----NK-EPP VAFLDSLV-- ---LGEWEDR MQRGLFRYDV TACETKVIPG EYGFIAQLNE GRHLKKRPTE
TcGGP-2     -G-ELYP--- S--------- ---------- ---------- ---------- ----EE-QPQ PSFL-NLL-- ---LEQWGDR MRRGLFRYDV TNCKTKIIPG KYGFIAQLNE GRHLKKRPTE
TpGGP       -L-AIDE--- C--------- ---------- ---------- ---------- ----QE-ELP LAFLDSLV-- ---LGEWEDR MQKGLFRYDV TACETKVIPG ECGFIAQLNE GRHLKKRPTE
VvGGP-1     -A-SSGD--- E--------- ---------- ---------- ---------- ----NK-EQP VPFLDSLV-- ---LGEWEDR MQKGLFRYDV TACETKVIPG EYGFIAQLNE GRHLKKRPTE
VvGGP-2     -R-VEVS--- S--------- ---------- ---------- ---------- ----EG-QPP AAFLDNLL-- ---LGQWEDR MSQGLFRYDV TLCETRIMPG NYGFIAQLNE GRHMKKRPTE
VcGGP       SSYSAFS--- SSIA------ ---------- -------VSG SEDGKV---- ----LE-VPS RSLLESAL-- ---MTLWEDR ADRGLFRYDV TLCPTRVLPG SRGFIAQLNE GRATKKRPTE
ZmGGP-E-1   -----GS--- Q--------- ---------- ---------- ---------- ----ED-AAS TKLLVDIL-- ---LSEWEDR MTRGLFRYDV TACETKVIPG NLGFVAQLNE GRHLKKRPTE
ZmGGP-E-2   ------T--- E--------- ---------- ---------- ---------- ----HE-GSN LSPFLLKL-- ---FNEWDDR KTRGLFHHDI SSCETKVLPG VHNFVATLIE GRDQKKRPTE
ZmGGP-1     -----GS--- Q--------- ---------- ---------- ---------- ----ED-AAS TKLLILSLVI CFAFWQWEDR MTRGLFRYDV TACETKVIPG NLGFVAQLNE GRHLKKRPTE
ZmGGP-2     ------T--- E--------- ---------- ---------- ---------- ----HE-GSN LSPFLLKL-- ---FNEWDDR KTRGLFHHDI SSCETKVLPG VHNFVATLIE GRDQKKRPTE
ZomGGP      ---GSAV--- A--------- ---------- ---------- ---------- ----GK-NSP IDFLNTLV-- ---LGQWEDR MAQGMFRYDV TACETKIIPG NHGFIAQLNE GRHLQKRPTE


            ....|....| ....|....| ....|....| ....|....| ....|....| ....|....| ....|....| ....|....| ....|....| ....|....| ....|....| ....|....|
                   610        620        630        640        650        660        670        680        690        700        710        720
AcGGP       FRVDKVLQPF DESKFNFTKV GQEEVLFQFE --A----SDD N--------- ------EV-- ------QFFP NAPVDV---- -----ENSPS VVAIN-VSP- IEYGHVLLIP RILECLPQRI
AcGGP-R-1   FRVDKVLQPF DGSKFNFTKV GQEEVLFQFE --A----SND N--------- ------EV-- ------QFFP NAPVDV---- -----ENSPS VVAIN-VSP- IEYGHVLLIP SILECLPQRI
AcGGP-R-2   FRVDQVLQPF DENKFNFTKV GQEEVLFRFE --Q----SND S--------- ------KS-- ------HFFT SSSVNL-D-- -----SNSPN VVAIN-VSP- IEYGHVLLIP RVLDCLPQRI
AdGGP       FRVDKVLQPF DESKFNFTKV GQEEVLFQFE --A----SDD N--------- ------EV-- ------QFFP NAPVDV---- -----ENSPS VVAIN-VSP- IEYGHVLLIP SILECLPRRI
AeGGP       FRVDKVLQPF DESKFNFTKV GQEEVLFQFE --A----SVD N--------- ------EV-- ------QFFP NAPVDV---- -----ENSPS VVAIN-VSP- IEYGHVLLIP RILECLPQRI
ArGGP       FRVDKVLQPF DESKFNFTKV GQEEVLFQFE --A----SDD N--------- ------EV-- ------QFFP NAPVDV---- -----ENSPS VVAIN-VSP- IEYGHVLLIP RILECLPQRI
AmhGGP-1    FRVDKVLQPF DGNKFNFTKV GQEEVLFQFE --A----SED G--------- ------EA-- ------YFTP GAPIDV---- -----ENSPS FVAIN-VSP- IEYGHVLLIP RIFECLPQRI
AmhGGP-2    FRVDQVLQPF DDNKFNFTKV GQEEALFRFE --P----SND M--------- ------TC-- -------YHP SAPVSL-E-- -----SESPN I-----VSP- IEYGHVLLIP RIFDCLPQRI
AncGGP-3    FRIDEVLQPF DGDKFNFTKI GQEELIFRFE --E----SSN G--------- ------KA-- ------GYFQ SAPIMS---- -----SSSPN IIAIN-VSP- IAHGHVLLIP RILDFLPQRI
AncGGP-1    FRVDCVLQPF DPAKFNFTKV GQEEVLFRFE --A----SGS H--------- ------KS-- ------SFSE RAPVDG---- -----TKAPN VLAIN-VSP- IEYGHVLLIP RVLDCLPQRI
AncGGP-2    FRVDQVLQPF DANKFNFTKV GQEEALFRFE --P----ATN A--------- ---------- ---------- -TPVDG---- -----TRSPS VVVIN-VSP- IEYGHVLLIP RVLDCLPQRI
AqcGGP      FRVDKVLQPF DENKFNFTKV GQEEVLFQFE EKV----NDD F--------- ------GA-- ------QFVA SAPIDT---- -----ENSPS VVAIN-VSP- IEYGHVLLIP KILEHFPQRI
AhGGP-1     FRVDKVLQSF DGTKFNFTKV GQEELLFQFE --A----GED A--------- ------QV-- ------QFFP CMPLDP---- -----ENSPN VVAIN-VSP- IEYGHVLLIP RVLDCLPQRI
AhGGP-2     FRVDKVLQPF DGNKFNFTKV GQEELLFQFE --A----SNN DDD------- -----SKI-- ------QFLP SIPLDA---- -----DNSPS VVAIN-VSP- IEYGHVLLIP RVLDCLPQRI
AlGGP-1     FRVDKVLQSF DGSKFNFTKV GQEELLFQFE --A----GED G--------- ------QV-- ------QFFP CMPLDP---- -----ENSPS VVAIN-VSP- IEYGHVLLIP RVLDCLPQRI
AlGGP-2     FRVDKVLQPF DGNKFNFTKV GQEELLFQFE --A----SDN DDD------- -----SQI-- ------QFLP SIPLDA---- -----DNSPS VVAIN-VSP- IEYGHVLLIP RVLDCLPQRI
AtGGP-1     FRVDKVLQSF DGSKFNFTKV GQEELLFQFE --A----GED A--------- ------QV-- ------QFFP CMPIDP---- -----ENSPS VVAIN-VSP- IEYGHVLLIP RVLDCLPQRI
AtGGP-2     FRVDKVLQPF DGNKFNFTKV GQEELLFQFK --A----STN DDD------- -----SEI-- ------QFLA SMPLDA---- -----DNSPS VVAIN-VSP- IEYGHVLLIP RVLDCLPQRI
BosGGP-1    FRVDKVLQSF DGSKFNFTKV GQEELLFQFE --A----GED G--------- ------QV-- ------QFFP CMPLDP---- -----ENSPS VVAIN-VSP- IEYGHVLLIP RVLDCLPQRI
BosGGP-2    FRVDKVLQPF DGNKFNFTKV GQEELLFHFE --A----SND DDD------- -----SRI-- ------QFFP STPLDA---- -----DNSPS VVAIN-VSP- IEYGHVLLIP RVLDSLPQRI
BdGGP-1     FRVDRVLQPF DAAKFNFTKV GQEEVLFQFE --N----GGG D--------- ------DS-- ------YFLK SAPTTV-A-- -----DRAPN VVAIN-VSP- IEYGHVLLIP RVLDRLPQRI
BdGGP-2     FRVDRVLQPF HSAKFNFTKV GQEEVLFRFE --N----GGG D--------- ------SS-- ------YFLA NVPNTE-S-- -----SHPPS VVAIN-VSP- IEYGHVLLIP RVLDRLPQRI
BdGGP-3     FGMNQVLQPF DSGKFNFTKV RPEEVIFTFH --E----TED E--------- ------SD-- ------RYFD GAPPTV---- -----LASPS SILIN-VSP- IGYCHVLLIP RILECLPQRV
BsGGP-1     FRVDRVLQPF DAAKFNFTKV GQEEVLFQFE --N----GGG D--------- ------DS-- ------YFLK SAPITV-A-- -----DCAPN VVAIN-VSP- IEYGHVLLIP RVLDRLPQMI
BsGGP-2     FRVDRVLQPF DSAKFNFTKV GQEEVLFRFE --N----GGG D--------- ------RS-- ------YFLA NAPNTE-S-- -----SHPPS VVAIN-VSP- IEYGHVLLIP RVLDRLPQRI
BsGGP-3     FGMNQVLQPF DSGKFNFTKV RPEEVIFRFQ --E----TEN E--------- ------SD-- ------RYFD GAPPTV---- -----LGSPS SILIN-VSP- IGYCHVLLIP RIQECLPQRV
BoGGP-1     FRVDKVLQSF DGNKFNFTKV GQEELLFQFE --A----GED S--------- ------EV-- ------QFFP CMPLDA---- -----ENSPS VVAIN-VSP- IEYGHVLLIP RVLDCLPQRI
BoGGP-2     FRVDKVLQSF DGSKFNFTKV SQEEMLFQFE --A----GHD D--------- ------EA-- ------RFFP CMPLVA---- -----ENSPS VVAII-VSP- IEYGHVLLIP RVLDCLPQRM
BoGGP-3     FRVDQVLQPF DANKFNFTKV SPEELLFQFE --A------- ---------- ---------- ---------- NMPLDA---- -----DNSPR VVAIN-VSP- IEYGHVLLIP QVFDCLPQRI
BrGGP-1     FRVDKVLQSF DGNKFNFTKV GQEELLFQFE --A----GED G--------- ------EV-- ------QFFP CMPLDS---- -----ENSPS VVAIN-VSP- IEYGHVLLIP RVLDCLPQRI
BrGGP-2     FRVDKVLQSF DGNKFNFTKV GQEELLFQFE --A----GED C--------- ------DV-- ------QFFP CMPLDA---- -----ENSPS VVAIN-VSP- IEYGHVLLIP RVLDCLPQRI
BrGGP-3     FRVDKVLQSF DGNKFNFTKV SQEELLFQFE --A----GEY E--------- ------EA-- ------QFLP CMPLLA---- -----ENSPS VVAIN-VSP- IEYGHVLLIP RVLDCLPQRM
BrGGP-4     FPVDQVLQPF DVNKFNFTKV SQEELLFQFE --A------- ---------- ---------- ---------- DLPLEA---- -----GNSPS VVAIN-VSP- IEYGHVLLIP QVLDCLPQRI
CgGGP-1     FRVDKVLQSF DGSKFNFTKV GQEELLFQFE --A----GED D--------- ------QV-- ------QFFP CMPLDP---- -----ENSPS VVAIN-VSP- IEYGHVLLIP RVLDCLPQKI
CgGGP-2     FRVDKVLQPF DGNKFNFTKV GQEELLFQFE --A----RSD DDD------- -----SGI-- ------QFLP SIPLDA---- -----DNSPS VVAIN-VSP- IEYGHVLLIP RVLECLPQRI
CrGGP-1     FRVDKVLQSF DGSKFNFTKV GQEELLFQFE --A----GED D--------- ------QV-- ------QFFP CMPLDP---- -----ENSPS VVAIN-VSP- IEYGHVLLIP RVLDCLPQKI
CrGGP-2     FRVDKVLQPF DGNKFNFTKV GQEELLFQFE --A----RSD DDD------- -----SGI-- ------QFFP SIPLDA---- -----DNSPS VVAIN-VSP- IEYGHVLLIP RVLECLPQRI
ChrGGP      FSADRVMQPF DPARFHFNKA AMGEVLFAFQ --A----DAT ASA------- ------TSAT ATAAPRLLLP SAPMAKSALL ASNPVSGSPN LVLIN-VSP- IDHCHVLLVP RVLDCLPQAL
CcGGP-1     FRVDKVLQPF DGNKFNFTKV GQEEVLFQFE --A----SED G--------- ------EV-- ------QFHP SAPIDV---- -----ENSPS VVAIN-VSP- IEYGHVLLIP RVLECLPQRI
CcGGP-2     FRIDQVLQPF DENKFNFTKI GQEEMLFRLD --D------- ---------- ---------- ---------- ---------- -----SKSPS VVAIN-VSP- IEYGHVLLIP RVLDCLPQRI
CsGGP-1     FRVDKVLQPF DGNKFNFTKV GQEEVLFQFE --A----SED G--------- ------EV-- ------QFHP SAPIDV---- -----ENSPS VVAIN-VSP- IEYGHVLLIP RVLECLPQRI
CsGGP-2     FRIDQVLQSF DENKFNFTKI GQEEMLFRLD --D------- ---------- ---------- ---------- ---------- -----SKSPS VVAIN-VSP- IEYGHVLLIP RVLDCLPQRI
CosGGP      FCVDQVVQRF DNGKFNFTKA LQKEVLFQFE --A----ADM SSK------- ------GS-- ------AFLP LAPV------ -----SGSPN LVFIN-VSP- IEYGHVLLVP RALDRLNQLV
CusGGP      FRVDKVLQPF DGNKFNFTKV GQEEVLFRFE --A----NEN G--------- ------NT-- ------QFIP NDAIDL---- -----ENSPS VVAIN-VSP- IEYGHVLLIP RILDCLPQRI
DcGGP-1     FRVDKVLQPF DGSKFNFTKV GQEEVLFQFK --A----SEE D--------- ------EI-- ------HFFP DASIDV---- -----GNSPS VVAIN-VSP- IEYGHVLLIP HVLECLPQRI
DcGGP-2     FRVDKVLQPF DGSKFNFTKV GQEEVIFQFE --A----SED G--------- ------EI-- ------QFFP NAPIDV---- -----ANSPS VVAIN-VSP- IEYGHVLLIP RVLECLPQRI
DsGGP       VVVDKVCQPF TEAKFNFKKA YLKEVLFQFE --P----RSA SGAHINDPSA MANKDDEA-- --SYEPLLLD SAAA------ -----SNSPN LVLIN-VSP- IEYGHVLLVP RVLDSLPQAA
EgGGP-1     FRVDKVLQPF DGSKFNFTKV GQEEVLFQFE --A----SVD G--------- ------EA-- ------QFFP NAPIDV---- -----ESSPS VVAIN-VSP- IEYGHVLLIP RVLECLPQRI
EgGGP-2     FRVDKVLQPF DGSKFNFTKV GQEEILFQFE --A----SED G--------- ------EV-- ------QFFA KAPIDA---- -----DNSPS VVAIN-VSP- IEYGHVLLIP RVLECMPQRI
```

```
EgGGP-3      FRVDQVLQDF DEKKFNFTKV GHEEVLFMFE --Q----SDD C--------- ------YS-- ------HFFP SAPAIF-K-- -----PTSPN VVAIN-VSPS IQYGHILLIP RVLDCLPQRI
EgGGP-4      FRVDQVLQDF DEKKFNFTKV GQEEVLFMFE --Q----SDD C--------- ------YS-- ------HFFP SAPAIF-K-- -----PTSPN V-----VSP- IEYGHVLLIP RVLDCLPQRI
EgGGP-5      FRVDQVLQDF DEKKFNFTKV GHEEVLFMFE --Q----SDD C--------- ------YS-- ------HFFP SAPASF-K-- -----PTSPN VVAIN-VSP- IEYGHILLIP HVLDCLPQRI
EsGGP-1      FRVDKVLQSF DGNKFNFTKV GQEELLFQFE --A----GED G--------- ------EV-- ------QFFP CMPLDA---- -----ENSPS VVAIN-VSP- IEYGHVLLIP RVLDCLPQRI
EsGGP-2      FRVDKVLQPF DGNKFNFTKV GQEELLFQFE --A----TDD DDD------- -----REI-- ------QFFP NMPLDA---- -----DNSPS VVAIN-VSP- IEYGHVLLIP CVLDCLPQRI
FvGGP-1      FRVDKVLQPF DGSKFNFTKV GQEEVLFQFE --A----SED G--------- ------EV-- ------QFHP NAPIDV---- -----ENSPS VVAIN-VSP- IEYGHVLLIP RILESLPQRI
FvGGP-2      FRVDKVLQAF DGSKFNFTKV GQEEVLFQFE --A----SED G--------- ------EV-- ------QFHP NAPIDV---- -----ENSPN VVAIN-VSP- IEYGHVLLIP RILESLPQRI
FvGGP-3      FRVDKVLQPF DGSKFNFSKV GQEEVLFQFE --A----SED G--------- ------EV-- ------QFHP NAPIDI---- -----ATSPS VVAIN-VSP- IEYGHVLLIP RILESLPQRI
GmGGP-1      FRVDKVLQPF DENKFNFTKV GQEEVLFQFE --A----SED G--------- ------QV-- ------QFFP NAPVDV---- -----DNSPS FVAIN-VSP- IEYGHVLLIP RIFECLPQRI
GmGGP-2      FRVDKVLQPF DENKFNFTKV GQEEVLFQFE --A----SED G--------- ------QV-- ------QFFP NAPVDV---- -----DNSPS FVAIN-VSP- IEYGHVLLIP RIFECLPQRI
GmGGP-3      FRVDKVLQPF DENKFNFTKV GQEEVLFQLE --A----SND G--------- ------EV-- ------QFFP NAPIDV---- -----ENSPS FVAIN-VSP- IEYGHVLLIP RIFECLPQRI
GmGGP-4      FRVDKVLQPF DENKFNFTKV GQEEVLFQLE --A----SND G--------- ------EA-- ------QFFP NVPIDV---- -----ENSPS FVAIN-VSP- IEYGHVLLIP QIFECLPQRI
GnmGGP       FRVDKVLQDF DPKKFNFTKV GQEEMLFRFE --E----SAA G--------- ------IT-- ------EFLE KGPV------ -----LDSPN VIVIN-VSP- IEYGHVLLVP RVLDCLPQRI
GrGGP-1      FRVDKVLQPF DGNKFNFTKV GQEEVLFQFE --A----SED G--------- ------EV-- ------QFYP NAPIDV---- -----ESSPS VVAIN-VSP- IEYGHVLLIP RIFECLPQRI
GrGGP-2      FRVDKVLQPF DGNKFNFTKV GQEEVLFQFE --A----SED G--------- ------EV-- ------QFYP SAPIDV---- -----ENSPS VVAIN-VSP- IEYGHVLLIP RILECLPQRI
GrGGP-3      FRVDKVLQPF DGNKFNFTKV GQEEVLFQFE --A----SED G--------- ------EV-- ------QFFP NAPIDV---- -----ENHPS VVAIN-VSP- IEYGHVLLIP RILECLPQRI
KfGGP-1      FRVDKVLQPF DEKKFNFTKI GQEEIVFQFE --A----SEC G--------- ------EV-- ------QFFP EAPIEV---- -----ENRPN VVAIN-VSP- IEYGHVLLIP RIFECLPQRI
KfGGP-2      FRVDRVLQPF DQKKFNFTKI GQEEILFQFE --A----SEC G--------- ------EI-- ------QYFP EAPIHV---- -----ENHPS VVAIN-VSP- IEYGHVLLIP RIFECLPQRI
KlGGP-1      FRVDKVLQPF DEKKFNFTKI GQEEIVFQFE --A----SEC G--------- ------EV-- ------QFFP EAPIEV---- -----ENRPN VVAIN-VSP- IEYGHVLLIP RIFECLPQRI
KlGGP-2      FRVDKVLQPF DQKKFNFTKI GQEEILFQFE --A----SEC G--------- ------EI-- ------QYFP EAPIHV---- -----ENHPS VVAIN-VSP- IEYGHVLLIP RIFECLPQRI
KlGGP-3      FRVDKVLQPF DQKKFNFTKI GQEEILFQFE --A----SEC G--------- ------EI-- ------QYFP EAPIHV---- -----ENHPS VVAIN-VSP- IEYGHVLLIP RIFECLPQRI
LuGGP        FRVDKVLQPF DGNKFNFTKV GQEEVLFQFD --A----SEN G--------- ------QV-- ------EFFP DAPVDA---- -----DKSPS VVAIN-VSP- IEYGHVLLIP RILECLPQRI
MdGGP        FRVDKVLQPF DSSKFNFTKV GQEEVLFRFE --A----SED G--------- ------EV-- ------HFFP SAPIDV---- -----ENSPS VVAIN-VSP- IEYGHVLLIP RIFERLPQRI
MeGGP-1      FRVDKVLQPF DGNKFNFTKV GQEEVLFQFE --P----SEN G--------- ------EV-- ------QFFP SASIDV---- -----DNSTS VVAIN-VSP- IEYGHVLLIP RILECLPQRI
MeGGP-2      FRVDKVLQPF DGNKFNFTKV GQEEVLFQFE --S----SED G--------- ------DV-- ------QFFP SAPIDV---- -----DNSPS VVAIN-VSP- IEYGHVLLIP RILECLPQRI
MeGGP-3      FRVDKVLQAF DETKFNFTKI GQEEVLFRFE --P----GND L--------- ------KS-- ------EFVP TAPPTA-D-- -----STSPS VVAIN-VSP- IEFGHVLLIP RVLDCLPQRI
MpGGP        FRVDQVLQPY DAKKFNFTKV DQQEILFAFD --E----SSD H--------- ------CS-- ------EYSE QAFV------ -----GESPN VVAIN-VSP- IEYGHVLLVP QVKSQIPQRI
MtGGP-1      FRVDKVLQPF DENKFNFTKV GQEEVLFQFE --A----SED G--------- ------EV-- ------QFYP NAPIDV---- -----DNYPS FVAIN-VSP- IEYGHVLLIP RIFECLPQRI
MtGGP-2      FRVDKVLQPF DETKFNFTKV GQEEILFQFK --S----SSD G--------- ------ET-- ------QFFP DAPIDV---- -----ENSPS FVAIN-VSP- IEYGHVLLIP RIFECLPQRI
MpGGP        FKVDQVCQEF DAGKFNFTKA DKAEILFRFS --P----GGV GQT------- ------RS-- ------EYVA SAPIEYADAD RVRGESDAPT VVFIN-VSP- IEYGHVLLTP RVTDCLPQRI
MsGGP        FAVDEVVQAF DGGKFNFTKA DKAEILFAFE --R----GDR AMK------- ------SS-- ------AYNS AKTI------ -----ESSPN VMLIN-VSP- IEYGHVLLCP RVTDCLPQRI
MgGGP-1      FRVDKVLQPF DETKFNFTKV GQEEVLFQFE --P----SEN N--------- ------HV-- ------QFFP NAPIDL---- -----ENSPS VVAIN-VSP- IEYGHVLLIP RIFECLPQRI
MgGGP-2      FRVDKVLQPF DETKFNFTKV GQEEVLFQFE --A----SED N--------- ------EV-- ------HYFP NAPIDS---- -----DNSPS VVAIN-VSP- IEYGHVLLIP RILEQLPQRI
MaGGP-1      FRVDRVLQPF DPAKFNFTKV GQEEVLFRFE --A----GEG D--------- ------KA-- ------RFLE SAAVGE---- -----TNTPS VVAIN-VSP- IEYGHVLLIP RVLDCLPQRI
MaGGP-2      FRVDRVLQAF DPAKFNFTKV GQEEVLFRFE --A----AGG D--------- ------EA-- ------HLFE SDGNEG---- -----TKSPS VVAIN-VSP- IEYGHVLLIP RVLDSLPQRI
MaGGP-3      FRVDRVLQPF DRAKFNFTKV GQEEVLFRFE --P----GES G--------- ------SG-- ------RLLE DDAVGD---- -----ANAPN VVAIN-VSP- IEYGHVLLIP RVLDCLPQRI
MaGGP-4      FRIDRVLQPF DGKKFNFTKI AQEEMLIRFE --E----SEN D--------- ------DR-- ------GFFE NTPIAD---- -----SESPN VVAIN-VSP- IDYGHVLLIP RILDCLPQRL
OtGGP        FRVDRVLQPF DPAKFNFTKV GQEEVLFQFE --S----STG D--------- ------DS-- ------YFLE SASIPA---- -----NRAPN VVAIN-VSP- IEYGHVLLIP RVLDRLPQRI
OsGGP-1      FRVDRVLQPF DAAKFNFTKV GQEEVLFQFE --N----GGG D--------- ------DS-- ------FFVE SSPISV-A-- -----DRAPN VVAIN-VSP- IEYGHVLLIP RVLDRLPQRI
OsGGP-2      FGMNQVLQPF DSVKFNFTKV SPEEVIFTFK --E----SQN D--------- ------SV-- ------KYFD NVPHAV---- -----AASPT AILIN-VSP- IGYCHVLLIP RIQDCLPQRV
OlGGP        FSVDKVCQDF DANKFNFTKA DLKEVLFSFT --KLAGEADE NVS------- ------RS-- ------VFEP SAAV------ -----GESPT VVLIN-VSP- IEYGHVLLCP RVTDMLPQQI
PhGGP-1      FRVDRVLQPF DPAKFNFTKV GQEEVLFQFE --N----GAG D--------- ------DS-- ------YFLN NAPVTA-A-- -----DRAPN VVAIN-VSP- IEYGHVLLIP RVLDRLPQRI
PhGGP-2      FGMNQVLQPF DSGKFNFTKV RPEEVIFRFC --E----TDK D--------- ------SA-- ------QYFG GAPDTI---- -----SASSS AILIN-VSP- IGYCHVLLTP KIQDCLPQRI
PvGGP-1      FRVDRVLQPF DPAKFNFTKV GQEEVLFQFE --N----GAG D--------- ------DS-- ------YFLN NAPITA-A-- -----DRAPN VVAIN-VSP- IEYGHVLLIP HVLDRLPQKI
PvGGP-2      FRVDRVLQPF DPAKFNFTKV GQEEVLFQFE --N----GAG D--------- ------DS-- ------YFLN NAPITA-A-- -----DRAPN VVAIN-VSP- IEYGHVLLIP RVLDRLPQRI
PvGGP-3      --------PF DSGKFNFTKV RPEEVIFRFC --E----TDK D--------- ------SA-- ------QYFG GAPDTI---- -----SASSS VILIN-VSP- IGYCHVLLTP KIQDCLPQRI
PhvGGP-1     FRVDKVLQPF DESKFNFTKV GQEEVLFQFE --A----SDD G--------- ------QI-- ------QFFP NAPVDV---- -----DNSPS FVAIN-VSP- IEYGHVLLIP RIFECLPQRI
PhvGGP-2     FRVDKVLQPF DESKFNFTKV GQEEVLFQLE --P----SDD G--------- ------EA-- ------QFSP NAPIDV---- -----DNSPS FVAIN-VSP- IEYGHVLLIP RIFECLPQRI
PpGGP-1      FRIDQVLQPF DPKKFNFTKV GQEEVLFQFM --P----NDS D--------- ------MS-- ------EYYE KATV------ -----SSSPN VVAIN-VSP- IEYGHILLVP RVLDRLPQRI
PpGGP-2      FRVDQVLQAF DPKKFNFTKV GQEEVLFQFG --P----SED E--------- ------VS-- ------DYYE KAPV------ -----LASPN VIAIN-VSP- IEYGHVLLVP RVLDRLPQRI
PpGGP-3      FRVDQVLQPF DSKKFNFTKV GQEEVLFQFG --P----SED G--------- ------VS-- ------EYYE KALV------ -----LNSPN VVAIN-VSP- IEYGHALLVP RVLDCLPQRI
PaGGP-1      FRVDKVLQDF DPSKFNFTKV GQEEVLFRFE --E----SEE N--------- ------KV-- ------QYLE KALV------ -----LDSPN VIAIN-VSP- IDYGHVLLVP RVLDCLPQRI
PaGGP-2      FRVDKVLQGF DPSKFNFTKV GQEEVLFTFE --E----SEE N--------- ------KV-- ------QYLE KAPV------ -----LDSPN VIAIN-VSP- IEYGHVLLVP RVLDCLPQRI
PaGGP-3      FRVDKVQQDF DPSKFNFTKV GQEEVLFRFE --E----SGE D--------- ------KV-- ------QYLE KAPV------ -----LDSPN VIAIN-VSP- IEYGHVLLVP RVLDCLPQRI
PsGGP-1      FRVDKVLQDF DPSKFNFTKV GQEEVLFRFE --E----SAE N--------- ------KV-- ------QYLE KALV------ -----LDSPN VIAIN-VSP- IDYGHVLLVP RVLDCLPQRI
PsGGP-2      FRVDKVQQDF DRSKFNFTKV GQEEVLFRFE --E----SEE G--------- ------KV-- ------QYLE KAPV------ -----LDSPN VIAIN-VSP- IEYGHVLLVP RVLDCLPQRI
PsGGP-3      FRVDKVQQDF DPSKFNFTKV GQEEVLFRFE --E----SGE D--------- ------KV-- ------QYLE KAPV------ -----LDSPN VVAIN-VSP- IEYGHVLLVP RVLDCLPQRI
PtGGP-1      FRVDKVLQDF DPSKFNFTKV GQEEVLFRFE --E----SEE N--------- ------KV-- ------QYLE KALV------ -----LDSPN VIAIN-VSP- IEYGHVLLVP RVLDCLPQRI
PtGGP-2      FRVDKVLQDF DPCKFNFTKV GQEEVIFRFE --E----SEE D--------- ------KV-- ------QYLE KAPV------ -----LDSPN VIAIN-VSP- IEYGHVLLVP RVLDCLPQRI
PtGGP-3      FRVDKVLQGF DPSKFNFTKV GQDEVLFRFE --E----SEE N--------- ------KI-- ------QYLE KAPV------ -----FDSPN VIAIN-VSP- IEYGHVLLVP RVLDCLPQRI
PtGGP-4      FRVDKVQQDF DPSKFNFTKV GQEEVLFRFE --E----SEE D--------- ------KV-- ------QYLE KAPV------ -----LDSPN VIAIN-VSP- IEYGHVLLVP RVLDCLPQRI
PtGGP-5      FRVDKVLQDF DPCKFNFTKV GQEEVLFRFE --E----NEE G--------- ------KV-- ------QYLE KAPV------ -----LDSPN VIAIN-VSP- VEYGHVLLVP RVLDCLPQRI
PotGGP-1     FRVDKVLQPF DGNKFNFTKV GQEEVLFQFG --A----SED G--------- ------EV-- ------QFFP DAPIDP---- -----ENSPS MVAIN-VSP- IEYGHVLLIP RVLDCLPQRI
PotGGP-2     FRVDKVLQPF DGNKFNFTKV GQEEILFQFG --E----SED G--------- ------EV-- ------KFFP DATIDA---- -----ENSPS VVAIN-VSP- IEYGHVLLIP RVLDCLPQRI
PotGGP-3     FRVDKVLQDF DETKFNFTKV GH----FRFE --K----SID H--------- ------NR-- ------HFFP SAPPITAD-S ---NSSNSSS VVAIN-VSP- IEYGHVLLIP QVLNCLPQRI
PrpGGP       FRVDKVLQPF DGNKFNFTKV GQEEVLFQFE --A----SED G--------- ------EV-- ------QFIP SAPIEP---- -----ENSPS VVAIN-VSP- IEYGHVLLIP RILEHLPQRI
RcGGP-1      FRVDKVLQPF DGNKFNFTKV GQEEVLFQFE --A----SED G--------- ------DI-- ------QFFP SAPIDL---- -----KNSPS VVAIN-VSP- IEYGHVLLIP RILECLPQRI
RcGGP-2      FRVDKVLQPF DGNKFNFTKV GQEEVLFQFE --A----SED D--------- ------DI-- ------QFFP SAPIDV---- -----ENSPS VVAIN-VSP- IEYGHVLLIP RILDCLPQRI
RcGGP-3      FRVDNVLQSF DDSKFNFTRI GQEEVLFRFE --Q----GQE N--------- ------MS-- ------HFFP NPPPSADD-- -----SSSPS IVAIN-VSP- IEFGHVLLIP RVLDCFPQRI
SpGGP-1      FRIDKVLQPF DENKFNFTKV GQEEMLFQFG --E----SED G--------- ------EG-- ------RFFT DATIDP---- -----ENSPS VVAIN-VSP- IEYGHVLLIP RVLDCLPQRI
SpGGP-2      FRVDKVLQPF DGNKFNFTKV GQEEVLFQFG --A----SEE G--------- ------EA-- ------QFFP DAPVDP---- -----ENSPT MVAIN-VSP- IEYGHVLLIP RVLDCLPQRI
SpGGP-3      FRVDKVLQDF DETKFNFTKV GQEEVLFRFE --K----SND H--------- ------KR-- ------RFFP TAPPVTAD-- -----SDSSR VVAIN-VSP- IEYGHVLLIP QVLNCLPQKI
SmGGP-1      FRVDQVLQPF DHKKFNFTKV GQEEMLFCFE --Q----SNN D--------- ------KS-- ------HYHS KAYI------ -----RGSPN VVVIN-VSP- IEYGHILLVP RVLDCIPQHL
SmGGP-2      FKMDQVLQDF DPSKFNFTKV GQEEVLFCFD --P------- ---------- ---------- ---------- ---------- -----ENSPF ATSSNMVSP- IEFGHILLIP RLLNCLPQRL
SmGGP-3      FRVDQVLQEF DPKKFNFSKV NRNEVLFQFG --E----GE- ---------- ------EA-- ------GYYD ITPI------ -----LSSPD FVLIN-VSP- IEYGHVLLVP RLFDFVPQRL
SiGGP-1      FRVDRVLQPF DPAKFNFTKV GQEEVLFQFG --N----GAG D--------- ------DS-- ------YFLN NAPITV-A-- -----DRAPN VVAIN-VSP- IEYGHVLLIP RVLDGLPQRI
SiGGP-2      FGMNQVLQPF DSGKFNFTKV RPEEVIFRFC --E----ADK D--------- ------SA-- ------QYFN DAPDTV---- -----SSSSS AILIN-VSP- IGYCHVLLTP QIQDCLPQRI
SvGGP-1      FRVDRVLQPF DPAKFNFTKV GQEEVLFQFE --N----GAG D--------- ------DS-- ------YFLN NAPITV-A-- -----DRAPN VVAIN-VSP- IEYGHVLLIP RVLDGLPQRI
SvGGP-2      FGMNQVLQPF DSGKFNFTKV RPEEVIFRFC --E----ADK D--------- ------SA-- ------QYFN DAPDTI---- -----SSSSS AILIN-VSP- IGYCHVLLTP QIQDCLPQRI
SlGGP-1      FRVDKVLQPF DGSKFNFTKV GQEELLFQFE --A----SEE D--------- ------EV-- ------QLYP DAPIDP---- -----EKSPS VVAIN-VSP- IEYGHVLLIP KVLECLPQRI
SlGGP-2      FCIDKVLQPF DENKFNFTKV GQEEVLFRFE --P----STD Y--------- ------KA-- ------HYFS GMRVNS---- -----GISPS IVAIN-VSP- IEYGHVLLIP RVLDCLPQRI
StGGP-1      FRVDKVLQPF DGSKFNFTKV GQEELLFQFE --A----SEE D--------- ------EV-- ------QLYP DAPIDP---- -----EKSPS VVAIN-VSP- IEYGHVLLIP KVLECLPQRI
StGGP-2      FCIDKVLQPF DENKFNFTKV GQEEVLFRFE --P----STD Y--------- ------KP-- ------HYFS GMRVNG---- -----GISPS IVAIN-VSP- IEYGHVLLIP RVLDCLPQRI
SbGGP-1      FRVDRVLQPF DPAKFNFTKV GQEEVLFQFE --N----GDG D--------- ------DS-- ------YFLN DAPIIA-V-- -----DRAPN VVAIN-VSP- IEYGHVLLIP RVLDRLPQKI
SbGGP-2      FTMNQVLQPF VSEKFNFTKV SPLEVIFRFN --E----TEK D--------- ------SA-- ------QYFD GVPDTV---- -----SASSS AILIN-VSP- IGYCHALLTP KIQDCLPQRI
SfGGP-1      FRVDQVLQPF DGKKFNFTKV GQEEVLFCFE --E----SED G--------- ------TS-- ------QYYE KAPV------ -----SWSPN VIAIN-VSP- IEYGHVLLIP CVLDCTPQRI
SfGGP-2      FRVDQVLQPY DGKRFNFTKV GQEEVLFCFE --E----SED G--------- ------TN-- ------QYYE KAPV------ -----SESPN VIAIN-VSP- IEYGHVLLVP RVLNCTPQRI
SppGGP-1     FRVDKVLQAF DSSKFNFTKV GQEEVLFRFE --P----SPD G--------- ------GAS- ------RYSE AAPVGA---- -----AASPN VVAIN-VSP- IEYGHVLLIP RVLDRFPQRI
SppGGP-2     FRVDQVLQPF DPSKFNFTKV GQEEVLFRFE --E----SAD G--------- ------KA-- ------HYFD HYPVGT---- -----ATHPN VVAIN-VSP- IEYGHVLLIP RVLECLPQRI
TcGGP-1      FRVDKVLQPF DGNKFNFTKV GQEEVLFQFA --A----SED G--------- ------EV-- ------QFFP NAPIDV---- -----ENSPS VVAIN-VSP- IEYGHVLLIP RILECLPQRI
TcGGP-2      FRIDQVLQPF DDSKFNFTKV GQEEVLFRFE --Q----SND C--------- ------KS-- ------HFLA SAPVTA-K-P -----NYSPN VIAIN-VSP- IEYGHVLLIP HVLDCLPQRI
TpGGP        FRVDKVLQPF DETKFNFTKV GQEEVLFQFE --A----SED G--------- ------EI-- ------QFFP NAPIDV---- -----DNSPS FVAIN-VSP- IEYGHVLLIP RIFERLPQRI
VvGGP-1      FRVDKVLQPF DGNKFNFTKV GQEEVLFQFE --P----SND E--------- ------EP-- ------EFIP NAPIDV---- -----ENSTS VVAIN-VSP- IEYGHVLLIP RIFECLPQRI
VvGGP-2      FRVDQVLQPF DENKFNFTKI GQEEVLFRFE --Q----SND N--------- ------NA-- ------HYFP VSPVTA-D-- -----SNSSS VVAIN-VSP- IEYGHVLLIP HVLDCLPQRI
VcGGP        VTLDRVLQPF DSAKFNFKKA AMAEALVGFF --P----DDA GSG-GGAGGG NGG---RS-- ------LLPS VAPLGAAAVA A----GGSPN LVLIN-VSP- IDYGHVLLVP RVLDNLPQAL
ZmGGP-E-1    FRVDRVLQPF DPAKFNFTKV GQEEVLFQFE --N----SGG D--------- ------DS-- ------YFLN NAPIIA-V-- -----DRAPN VIAIN-VSP- IEYGHVLLIP RVLDRLPQRI
ZmGGP-E-2    FTMNQVLQPF VSEKFNFTKV SPLEVIFRFN --D----TEK D--------- ------SA-- ------QYFD GVPDTV---- -----SASSS AILIN-VSP- IGYCHVLLTP KIQDCLPQRI
ZmGGP-1      FRVDRVLQPF DPAKFNFTKV GQEEVLFQFE --N----SGG D--------- ------DS-- ------YFLN NAPIIA-V-- -----DRAPN VIAIN-VSP- IEYGHVLLIP RVLDRLPQRI
ZmGGP-2      FTMNQVLQPF VSEKFNFTKV SPLE------ ---------- ---------- ---------- ---------- ---------- ---------- ------VSP- IGYCHVLLTP KIQDCLPQRI
ZomGGP       FRVDCVLQNF DSKKFNFTKV GQKEVLFRFE --P----S-D G--------- ------EP-- ------RYFE DAKVSP---- -----DSSGN VLAIN-VSP- IEYGHVLLIP RVLDCLSQKI

             ....|....| ....|....| ....|....| ....|....| ....|....| ....|....| ....|....| ....|....| ....|....| ....|....| ....|....| ....|....|
                    730        740        750        760        770        780        790        800        810        820        830        840
AcGGP        DRESFLLALH MAAE------ --AGNPYFRL GYNSLGAFAT INHLHFQAYY LAVPFPIEKA PTRK-ITT-- ---------- ---------- ---------- -----LNGGV KISDLLNYPV
AcGGP-R-1    DRESFLLALH MAAE------ --AGNPYFRL GYNSLGAFAT INHLHFQAYY LAVPFPIEKA PTRK-ITT-- ---------- ---------- ---------- -----LNGGV KISELLNYPV
AcGGP-R-2    DHESFLLALH MAKE------ --TSEPFFRI GYNSLGAFAT INHLHFQGYY LSVSFPVEKA PTQR-IMIGK ---------- ---------- ---------- ---ELSDSGV KVSQLLNYPV
AdGGP        DRESFLLALH MAAE------ --AGNPYFRL GYNSLGAFAT INHLHFQAYY LXVPFPIEKV PTRK-ITT-- ---------- ---------- ---------- -----LNGGV KISELLNYPV
AeGGP        DRESFLLALH MAAE------ --AGNPYFRL GYNSLGAFAT INHLHFQAYY LAVPFPIEKA PTRK-ITT-- ---------- ---------- ---------- -----LNGGV KISELLNYPV
ArGGP        DRESFLLALH MAAE------ --AGNPYFRL GYNSLGAFAT INHLHFQAYY LAVPFPIEKA PTRK-ITT-- ---------- ---------- ---------- -----LNGGV KISDLLNYPV
AmhGGP-1     DYDSFLLALY MAIE------ --AGSPYFRL GYNSLGAFAT INHLHFQAYY LAAPFPIEKA PTKK-ITT-- ---------- ---------- ---------- -----TSNGV EIFELLKFPV
AmhGGP-2     DHASFLAALH FAKE------ --AANPFFRV GYNSLGAFAT INHLHFQAYY LQAPFPVEKA PTEA-ISKAN ---------- ---------- ---------- ---DMITTGI VISQLLEYPV
AncGGP-3     DCQSFLLAMY MVKA------ --ARNPYFRM GYNSLGAFAT INHLHFQAYY LEVKFPVEKA PTQI-IAT-- ---------- ---------- ---------- -----VGSGV NVAELLEYPV
AncGGP-1     DPESFLLAFH MAAE------ --AASPYFRL GYNSLGAFAT INHLHFQAYY LAVPFPVEKA PTQK-IPLVE ---------- ---------- ---------- ---GESKSGV KVSKLLNYPV
AncGGP-2     DRESFLLALH LAVE------ --AGNPYFRV GYNSLGAFAT INHLHFQAYY LSVPFPVEKA PTRR-IETLE ---------- ---------- ---------- ---G-PDGGV KVSQLLEYPV
AqcGGP       DRESFLLALH MAAE------ --VGNPYFRL GYNSLGAFAT INHLHFQAYY VYVPFPIEKA PTRK-ITT-- ---------- ---------- ---------- -----NSGGV KISELLNYPV
AhGGP-1      DHKSLLLAVH MAAE------ --AANPYFRL GYNSLGAFAT INHLHFQAYY LAMPFPLEKA PSKK-ITT-- ---------- ---------- ---------- -----TVSGV KISELLSYPV
                                                       HIT motif
```

```
AhGGP-2     DHKSLLLALQ MAAE------ --AANPYFRL GYNSLGAFAT INHLHFQAYY LAMQFPIEKA SFLK-ITT-- ---------- ---------- ---------- -----TNNGV KISKLLNYPV
AlGGP-1     DHKSLLLAVH MAAE------ --AANPYFRL GYNSLGAFAT INHLHFQAYY LAMPFPLEKS PSKK-ITT-- ---------- ---------- ---------- -----TVSGV KISELLSYPV
AlGGP-2     DHKSLLLALQ MAAE------ --AANPYFRL GYNSLGAFAT INHLHFQAYY LAMQFPIEKA SSLK-ITT-- ---------- ---------- ---------- -----TNNGV KISKLLNYPV
AtGGP-1     DHKSLLLAVH MAAE------ --AANPYFRL GYNSLGAFAT INHLHFQAYY LAMPFPLEKA PTKK-ITT-- ---------- ---------- ---------- -----TVSGV KISELLSYPV
AtGGP-2     DHKSLLLALQ MAAE------ --ADNPYFRL GYNSLGAFAT INHLHFQAYY LAMQFPIEKA SSLK-ITT-- ---------- ---------- ---------- -----TNNGV KISKLLNYPV
BosGGP-1    DHKSLLLAVH MAAE------ --AANPYFRL GYNSLGAFAT INHLHFQAYY LALPFPLEKA PSKK-MIT-- ---------- ---------- ---------- -----TVSGV KISELLNYPV
BosGGP-2    DHKSLLLALQ MATE------ --AANQYFRL GYNSLGAFAT INHLHFQAYY LAMQFPIEKA PSLK-ITT-- ---------- ---------- ---------- -----TNNGV KISKILNYPV
BdGGP-1     DQESLLLALH MAAE------ --AASPYFRL GYNSLGAFAT INHLHFQAYY LTVPFPVEKA ATQR-IPLAD ---------- ---------- ---------- ---DGMKSGV KVSKLMNYPV
BdGGP-2     DPESFLLALH MAAE------ --AASPYFRL GYNSLGAFAT INHLHFQAYY LSVPFPVEKA PTKK-IPLAK ---------- ---------- ---------- ---CELKSGV KVSKLMNFPV
BdGGP-3     DQENLLLAMY VARE------ --ARNPFFRV GYNSLGGFAT INHIHFQAYY LEVKYPVEKA PKEK-LTV-- ---------- ---------- ---------- -----LKNGV SISELVQYPV
BsGGP-1     DQESLLLALH MAAE------ --AASPYFRL GYNSLGAFAT INHLHFQAYY LTVPFPVEKA AVQR-IPLAD ---------- ---------- ---------- ---DGMKSGV KVSKLMDYPV
BsGGP-2     DPESFLLALH MAAE------ --AASPYFRL GYNSLGAFAT INHLHFQAYY LSVPFPVEKA PTKK-IPLAK ---------- ---------- ---------- ---CVLKSGV MVSKLMNFPV
BsGGP-3     DQESLLLAMY VARE------ --AKNPFFRV GYNSLGGFAT INHIHFQAYY LKVQYPVEKA PIEK-LAV-- ---------- ---------- ---------- -----IRNGV SICQLVQYPV
BoGGP-1     DHKSLLLALH MAAE------ --AANPYFRL GYNSLGAFAT INHLHFQAYY LAMPFPLEKA PSKK-MVT-- ---------- ---------- ---------- -----TASGV KISELLSYPV
BoGGP-2     DHKSMLLALH MASE------ --AKNPYFRV GYNSLGAFAT INHLHFQAYY LAMPFPLEKA SSKEMMIT-- ---------- ---------- ---------- -----TDNGV KISELLSYPM
BoGGP-3     DHKSLLLALH MAVE------ --AANPYFRV GYNSLSTFAT INHLHFQAYY LAMPFPIEKA HSLK-IST-- ---------- ---------- ---------- -----TNDGV RISKLMSYTV
BrGGP-1     DHKSLLLALH MAAE------ --AANPYFRL GYNSLGAFAT INHLHFQAYY LAMPFPLEKA PSKK-MIT-- ---------- ---------- ---------- -----TASGV KISELLSYPV
BrGGP-2     DHKSLLLALH MAAE------ --AANPYFRL GYNSLGAFAT INHLHFQAYY LAMPFPLEKA PSKK-MIT-- ---------- ---------- ---------- -----TVSGV KISELVNYPV
BrGGP-3     DHKSMLLALH MASE------ --AANPYFRV GYNSLGAFAT INHLHFQAYY LAMPFPLEKA ASRQ-MIT-- ---------- ---------- ---------- -----TDHGV KVSELLSYPM
BrGGP-4     DHKSLLLALH MAVE------ --AANPYFRV GYNSLGAFAT INHLHFQAYY LAMPFPIEKA HSLK-ITT-- ---------- ---------- ---------- -----TNDGV RISKLMSYPV
CgGGP-1     DHKSLLLAVH MAAE------ --AANPYFRL GYNSLGAFAT INHLHFQAYY LAMPFPLERA PSKK-MIT-- ---------- ---------- ---------- -----TASGV KISELLNYPV
CgGGP-2     DHKSLLLALQ MATE------ --ANNPYFRL GYNSLGAFAT INHLHFQAYY LAMQFPIEKA SSLQ-ITS-- ---------- ---------- ---------- -----TKDGV KISKLLNYPV
CrGGP-1     DHKSLLLAVH MAAE------ --AANPYFRL GYNSLGAFAT INHLHFQAYY LAMPFPLEKA PSKK-MIT-- ---------- ---------- ---------- -----TASGV KISELLNYPV
CrGGP-2     DHKSLLLALQ MATE------ --AANPYFRL GYNSLGAFAT INHLHFQAYY LAMQFPIEKA SSLK-ITS-- ---------- ---------- ---------- -----TKDGV KISKLLNYPV
ChrGGP      TPDTALLALQ FAAELGGSSS SRSGSGAFRV GYNSLGAFAT INHLHFHAYH LPAALPCERA PTCP-LPGAL ARP------- ------LAAS QQPRKRGAEE VAGAGSAGSV RVSRLVGYPV
CcGGP-1     DRDSFLLALY MAAE------ --AGNPYFRL GYNSLGAFAT INHLHFQAYY MALPFPIEKA PTKK-IIS-- ---------- ---------- ---------- -----TGSGV KISELLNYPV
CcGGP-2     DHESFLLALY VAKE------ --AADPFFRL GYNSLGAFAT INHLHFQAYY LPLPFPVEKA PISR-IVTVK ---------- ---------- ---------- ---GYQDTEV IISQLLNYPV
CsGGP-1     DRDSFLLALY MAAE------ --AGNPYFRL GYNSLGAFAT INHLHFQAYY MALPFPIEKA PTKK-IIS-- ---------- ---------- ---------- -----TGSGV KISELLNYPV
CsGGP-2     DHESFLLALY VAKE------ --AADPFFRL GYNSLGAFAT INHLHFQAYY LPLPFPVEKA PTSR-IVTVK ---------- ---------- ---------- ---GHQDTQV IISQLLNYPV
CosGGP      QPDTLKLALQ FAHE------ --AANPYFRL AFNSLGAYGT VNHLHFQAYY MAAPFAVERA PTVD-LCCLR ---------- ---------- ---------- -----KYRHV RVAMLADYPV
CusGGP      DRESFLLALH MATE------ --AGNTYFRL GYNSLGAFAT INHLHFQAYY LGVPFPIEKA PTKK-IMT-- ---------- ---------- ---------- -----LKDGV IISELLKYPV
DcGGP-1     DHESFLLALY MAAE------ --AGSPYFRL GYNSLGAFAT INHLHFQAYY LAVPFPIEKA STRK-VMN-- ---------- ---------- ---------- -----LDDGV EISELLGYPV
DcGGP-2     DHESLLLALY MAAE------ --AGSPYFRL GYNSLGAFAT INHLHFQAYY LAVPFPIEKT STKN-ITT-- ---------- ---------- ---------- -----LSGGI EISQLLNYPV
DsGGP       TSATFMQALH FARE------ --ADNPYFRV GYNSLGAYAT INHLHFQAYY LAAPFPCERA PTAL-LSRQQ T--------- ---------- ---------- ---ARLSPNL AVSRLASYPV
EgGGP-1     DRASFLLALY MASE------ --AGNPYFRL GYNSLGAFAT INHLHFQAYY LAVPFPVEKA STRN-ITA-- ---------- ---------- ---------- -----LDSGV NVSELVNYPV
EgGGP-2     DRESFSLALY MAAE------ --ARNPYFRL GYNSLGAFAT INHLHFQAYY LAVPFPIEKA PTQK-LTA-- ---------- ---------- ---------- -----LESGV TIFELVNYPV
EgGGP-3     DHMSFFLALS LAKE------ --AADPFFRV GYNSLGTFAT INHLHFQAYY LASPFPVEKV PTLR-VMTST ---------- ---------- ---------- ---SMQRNGV VVSQLLKFPV
EgGGP-4     DHMSFFLALS LAKE------ --AADPFFRV GYNSLGAFAT INHLHFQAYY LASPFPVEKV PTLR------ ---------- ---------- ---------- ---------- ---QLLKFPV
EgGGP-5     NRMSFFLALS LAKE------ --AADPFFRV GYNSLGTFAT INHLHFQAYY LASPFPVEKV PTLR-VMTST ---------- ---------- ---------- ---SMLRNGV VVSQLLKFPV
EsGGP-1     DHKSLLLAVH MAAE------ --AANPYFRL GYNSLGAFAT INHLHFQAYY LAMPFPLEKA LSKK-MIT-- ---------- ---------- ---------- -----TVSGV KISELLNYPV
EsGGP-2     DHKSLLLAIQ MATV------ --AANSYFRL GYNSLGAFAT INHLHFQAYY LAMQFPIEKA PSLK-ITT-- ---------- ---------- ---------- -----TNDCV KISKLLKYPV
FvGGP-1     DRESFLLALH MAAE------ --AGNPYFRL GYNSLGAFAT INHLHFQAYY LAVTFPIEKA PTKK-ITS-- ---------- ---------- ---------- -----LDGGV RISELLNYPV
FvGGP-2     DRESFLLALH MAAE------ --AGNPYFRL GYNSLGAFAT INHLHFQAYY LAVTFPIEKA PTKK-ITS-- ---------- ---------- ---------- -----LDGGV RISELLNYPV
FvGGP-3     DRESFLLALH MAAE------ --AGSPYFRL GYNSLGAFAT INHLHFQAYY LALTFPIEKA PTNK-ITS-- ---------- ---------- ---------- -----LDVGV KISELVNYPV
GmGGP-1     DRESFLLALQ MAVE------ --ADNPYFRL GYNSLGAFAT INHLHFQAYY LALPFPIEKA PTKR-IAS-- ---------- ---------- ---------- -----VNGGV KISELLKYPV
GmGGP-2     DRESFLLALQ MAVE------ --ADNPYFRL GYNSLGAFAT INHLHFQAYY LALPFPIEKA PTKK-IAS-- ---------- ---------- ---------- -----LNGGV KISELLKYPV
GmGGP-3     DHASFLLALQ MAVE------ --ARNPYFRL GYNSLGAFAT INHLHFQAYY LALPFPIEKA PTKK-IAK-- ---------- ---------- ---------- -----LSGGV IISKLLNYPV
GmGGP-4     DHASFLLALQ MAVE------ --ARNPYFRL GYNSLGAFAT INHLHFQAYY LALPFPIEKA PTKK-IAK-- ---------- ---------- ---------- -----LSGGV KISKLLNYPV
GnmGGP      DHDSFLLALH MAAE------ --ACNPFFRV GYNSLGAFAT INHLHFQAYF IAQPFPVEKA QTKK-VAWKS ---------- ---------- ---------- -----SGSGV RISELCNYPV
GrGGP-1     DRESFLLALH MAAE------ --AGNPYFRL GYNSLGAFAT INHLHFQAYY LAVPFPIERA PTKE-LTT-- ---------- ---------- ---------- -----LSDGV IVSELLKYPV
GrGGP-2     DRESFLLALY MTAE------ --AGNPYFRL GYNSLGAFAT INHLHFQAYY LAVPFPIEKA PTKK-ITT-- ---------- ---------- ---------- -----LYDEV IISELLSYPV
GrGGP-3     DHESFLLALH MAAE------ --AGNPYFRL GYNSLGAFAT INHLHFQAYY LAVAFPIEKA PTKK-KTS-- ---------- ---------- ---------- -----LNDGV IISELLKYPV
KfGGP-1     DHESFLLALH MAVE------ --AANPYFRL GYNSLGAFAT INHLHFQAYY LAVPFPIEKA PTKE-VAT-- ---------- ---------- ---------- -----LSSGV KISRLLQYPV
KfGGP-2     DNESLLLALH MAVE------ --AANPYFRL GYNSLGAFAT INHLHFQAYY LAVPFPIEKA PTNE-VVT-- ---------- ---------- ---------- -----LSSGV KIYKLLKYPV
KlGGP-1     DHESFLLALH MAVE------ --ASNPYFRL GYNSLGAFAT INHLHFQAYY LAVPFPIEKA PTKE-VAT-- ---------- ---------- ---------- -----LSSGV KISRLLQYPV
KlGGP-2     DNESLLLALH MAVE------ --AANPYFRL GYNSLGAFAT INHLHFQAYY LAVPFPIEKA PTNE-VVT-- ---------- ---------- ---------- -----LSSGV KIYKLLKYPV
KlGGP-3     DNESLLLALH MAVE------ --AANPYFRL GYNSLGAFAT INHLHFQAYY LAVPFPIEKA PTNE-VVT-- ---------- ---------- ---------- -----LSSGV KIYKLLKYPV
LuGGP       YRDSFLLALY MAVE------ --AGNPYFRL GYNSLGAFAT INHLHFQAYY LALPFPIEKA PTKK-VTT-- ---------- ---------- ---------- -----MGDGV KISYLIDYPV
MdGGP       DRESFLLALH MAAE------ --AGSPYFRL GYNSLGAFAT INHLHFQAYY LAVTFPIEKA PTKK-IST-- ---------- ---------- ---------- -----LNAEV KVSELLNYPV
MeGGP-1     DRESFLLALY MAAE------ --AANPYFRL GYNSLGAFAT INHLHFQAYY LAVQFPIEKA PTKK-ITT-- ---------- ---------- ---------- -----LDSGV KISELVNYPV
MeGGP-2     DRESFLLALH MAAE------ --AGNPYFRL GYNSLGAFAT INHLHFQAYY LAVQFPIEKA PTKK-ITT-- ---------- ---------- ---------- -----LDSGV KIYELVNYPV
MeGGP-3     DHESFLLALH MAKE------ --ASNPFFRI GYNSLGAFAT INHLHFQAYY LAVPFPVEKS PTKR-IKTVK ---------- ---------- ---------- ---GMQDRGV IVSRLSNFPV
MpGGP       CPETFLLALH MTVE------ --VNNPYFRL GYNSLGAFAT INHLHFQAYY LHVPFPIERA ETSR-LYQTR ---------- ---------- ---------- -----KKGGC KISQLVNYPV
MtGGP-1     DHESFLLALH MAAE------ --AANPYFRL GYNSLGAFAT INHLHFQAYY LAMPFPIEKA PTKK-IAT-- ---------- ---------- ---------- -----SNGGV KVSELLKYPV
MtGGP-2     DHANFSLALH MAVE------ --AGNPYFRL GYNSLGAFAT INHLHFQAYY LALPFPIEKA STKK-IAK-- ---------- ---------- ---------- -----LNGGV KVSKLLNYPV
MpGGP       SKDALLPALF MAAE------ --SRNPYFRV GYNSLGAYAT INHLHFQAYY LMEAFPIERA PTRA-LGLHV HAE------- ---------- ---------- --GGALSSGV VVKNVVDYPA
MsGGP       SPELLLPPLY MAAE------ --SRNPYFRV GYNSLGAYAT INHLHFQAYY LMEAFPIERA QTTR-LPQRV YK-------- ---------- ---------- --KRHRHGV AVNQVTGYPV
MgGGP-1     DRESFLLALH MAVE------ --AGNPYFRL GYNSLGAFAT INHLHFQAYY LATPFPIERA PSKK-ITT-- ---------- ---------- ---------- -----TSNGV EISYILNYPV
MgGGP-2     DGESFLLALH MAVE------ --TANPYFRL GYNSLGAFAT INHLHFQAYY LATPFPIERA PSKR-ITT-- ---------- ---------- ---------- -----TSGGV KISDILNYPV
MaGGP-1     DPDSFLLALH MAAE------ --AGSPYFRL GYNSLGAFAT INHLHFQAYF LTMPFPVEKA PTQR-IPVVK ---------- ---------- ---------- ---GLSQSKV KVSKLLNYPV
MaGGP-2     DQESFLLALR MASE------ --AGSPYFRL GYNSLGAFAT INHLHFQAYY LSVPFPVEKA PTAK-IPTNQ ---------- ---------- ---------- ---GRSKSGV TVSRLLNYPV
MaGGP-3     DPDSFLLALH MAAE------ --AGSPYFRL GYNSLGAFAT INHLHFQAYF LSMPFPVEKN STQR-IPIVG ---------- ---------- ---------- ---GLSHNGV KVSRLLNYPV
MaGGP-4     DEQSFSLAVY VARE------ --AETPHFRV AYNSLGAFAT INHLHFQAFY LVGAFPVEKA PTQR-IMT-- ---------- ---------- ---------- -----LASGV EIFQLSHYPV
OtGGP       DQESFLLALQ MAAE------ --AGSPYFRL GYNSLGAFAT INHLHFQAYY LSVPFPVEKA ATQK-IPLAE ---------- ---------- ---------- ---DSMKSGV NVSKLINYPV
OsGGP-1     DQESFLLALH MAAE------ --AASPYFRL GYNSLGAFAT INHLHFQAYY LTVPFPVEKA ATKR-IFLAE ---------- ---------- ---------- ---GTMNSGV KVSKLMNYPV
OsGGP-2     DKESFLLAMY VASE------ --AKDPFFRV GYNSLGGFAT INHLHFQAYY LKVQYPVEKA LTEK-LTT-- ---------- ---------- ---------- -----LGNGV SIIQLVQYPV
OlGGP       SPENLLPALY MAAE------ --SRNPYFRV GYNSLGAYAT INHLHFQAYY LMEAFPIERA NTVE-IFPG- ---------- ---------- ---------- -----THGDC TVYRVNGYPV
PhGGP-1     DPESFLLALQ MAAE------ --AASPYFRL GYNSLGAFAT INHLHFQAYY LSVPFPVEKA PTHK-IPLSE ---------- ---------- ---------- ---DTMKSGV TVSKLINYPV
PhGGP-2     DEESFLIAMY IARE------ --TRNPFFRV GYNSLGGFAT INHLHFQAYY LKVPYPVEKA TTEK-LTT-- ---------- ---------- ---------- -----LVNGV SISQLVDYPV
PvGGP-1     DPESFLLALQ MAAE------ --AASPYFRL GYNSLGAFAT INHLHFQAYY LSVPFPVEKA ATHK-IPLSE ---------- ---------- ---------- ---DTRKSGV TVSKLINYPV
PvGGP-2     DPESFLLALQ MAAE------ --AASPYFRL GYNSLGAFAT INHLHFQAYY LSVPFPVEKA ATHK-IPLSE ---------- ---------- ---------- ---DTMKSGV TVSKLINYPV
PvGGP-3     DEESFLIAMY VARE------ --ARNPFFRV GYNSLGGFAT INHLHFQAYY LKVKYPVEKA TTEK-LTT-- ---------- ---------- ---------- -----LVNGV SIAQLVDYPV
PhvGGP-1    DRESFLLALH MAVE------ --ADNPYFRL GYNSLGAFAT INHLHFQAYY LALPFPIEKA PTKK-IAN-- ---------- ---------- ---------- -----LNGGV KISELLKYPV
PhvGGP-2    DHESFLLALQ MAVE------ --AGNPYFRL GYNSLGAFAT INHLHFQAYY LAMPFPIEKA PTRK-ITT-- ---------- ---------- ---------- -----LSGGV KVSKLLHYPV
PpGGP-1     DQESFLLALY MAAE------ --ANNPYFRL GYNSLGAFAT INHLHFQAYY LAAPFPIERA PATR-VSY-- ---------- ---------- ---------- ---GRKKSGV KVYELTMFPV
PpGGP-2     DQDSFLLALH MAAE------ --ANNPYFRL GYNSLGAFAT INHLHFQAYY LAAPFPIERA PTVR-IPY-- ---------- ---------- ---------- ---GRKKGGV KVYELTEFPV
PpGGP-3     DQDSFLLALY MAAE------ --ANNPYFRL GYNSLG---- --------AYY LVAPFPIERA PTSR-ITY-- ---------- ---------- ---------- ---GRKKCGV KVHELTKFPV
PaGGP-1     DHDSLLLAMH LAAE------ --AGNTSFRL GYNSLGAFAT INHLHFQAYY LALPFPVEKA PTKR-APWKS ---------- ---------- ---------- -----EKGGV KISELCNYPV
PaGGP-2     DHDSLLLALH LAAE------ --VGNPSFRL GYNSLGAFAT INHLHFQAYY LNLPFPVEKA PKRR-VPWKS ---------- ---------- ---------- -----EKGGI NIFELLNYPV
PaGGP-3     DHDSLLLALH LAAE------ --AGNPSFRL GYNSLGAFAT INHLHFQAYY LALPFPVEKA PTKR-VPWKS ---------- ---------- ---------- -----EKAGV KIFELYNYPV
PsGGP-1     DHDSLLLAMH LAAE------ --AGNTSFRL GYNSLGAFAT INHLHFQAYY LALPFPVEKT PTKR-APWKS ---------- ---------- ---------- -----EKGGV KISELCNYPV
PsGGP-2     DHDSLLLALH LAAE------ --AGNPSFRL GYNSLGAFAT INHLHFQAYY LALPFPVEKA LTKR-VPWRS ---------- ---------- ---------- -----EKGGV KIFELLNYPV
PsGGP-3     DHDSLLLALH LAAE------ --ARNPSFRL GYNSLGAFAT INHLHFQAYY LALPFPVEKA PTKR-VPWKS ---------- ---------- ---------- -----EKAGV KIFELYNYPV
PtGGP-1     DHDSLLLAMH LAAE------ --AGNPYFRL GYNSLGAFAT INHLHFQAYY LALPFPVEKA PTKR-APWKS ---------- ---------- ---------- -----EKGGI KIFELCNYPV
PtGGP-2     DHDSLLLALH LAVE------ --AGNPSFRL GYNSLGAFAT INHLHFQAYY LARPFPVEKA STKR-VPWKS ---------- ---------- ---------- -----EKTGV KIFELCNYPV
PtGGP-3     DHDSLLLALH LAAE------ --AGNPYFRL GYNSLGAFAT INHLHFQAYY LSIPFPIEKA PTKR-VPWKS ---------- ---------- ---------- -----EKGGV NILELLDYPV
PtGGP-4     DHDSLLLALH LAAE------ --AGNPSFRL GYNSLGAFAT INHLHFQAYY LALPFPVEKA HTKR-VPLKS ---------- ---------- ---------- -----EKGGV KISKLCNYPV
PtGGP-5     DHDSLLLALH LAVE------ --AGNPSFRL GYNSLGAFAS INHLHFQAYY LAHPFTVEKA PTKR-VPWKS ---------- ---------- ---------- -----EKTGV KIFELCNYPV
PotGGP-1    DRDSFMLAIY MAAE------ --AGNPYFRL GYNSLGAFAT INHLHFQAYY LAVPFPIEKA PTKE-ITT-- ---------- ---------- ---------- -----SDGGV KISELVNYPV
PotGGP-2    DRDSFLLALH MAAE------ --AGDPYFRL GYNSLGAFAT INHLHFQAYY LTVPFPIEKA STKK-ITT-- ---------- ---------- ---------- -----LDGGV KISELVNYPV
PotGGP-3    DHGSFLLALH MAKE------ --AADPFFRV GYNSLGAFAT INHLHFQAYY LAAPFPVEKA PTRR-IMTMK ---------- ---------- ---------- ---SPQDEGV IVSQLLNYPV
PrpGGP      DRESFLLALH MAAA------ --AGNPYFRL GYNSLGAFAT INHLHFQAYY LAVTFPIEKA PTKK-ITV-- ---------- ---------- ---------- -----SGAGV RVSELLNYPV
RcGGP-1     DRESLLLALY MAAE------ --AGNPYFRL GYNSLGAFAT INHLHFQAYY LAMQFPIEKA PTNK-IAT-- ---------- ---------- ---------- -----LDSGV KISELVNYPV
RcGGP-2     DRDSLLLALY MAAE------ --AGNPYFRL GYNSLGAFAT INHLHFQAYY LAVQFPIEKA PTKK-ITT-- ---------- ---------- ---------- -----LDCGV KIAELVNYPV
RcGGP-3     DLDSFLLAVH LAKE------ --ASDPFFRV GYNSLGAFAT INHLHFQAYY LAATFLVEKA PSKK-IMIVE ---------- ---------- ---------- ---GSEGRGV IVSQLLNYPV
SpGGP-1     DRDSFLLALH MAAE------ --AGDPYFRL GYNSLGAFAT INHLHFQAYY LTMPFPIEKA STKQ-IAT-- ---------- ---------- ---------- -----LDNGV KISELMNYPV
SpGGP-2     DCDSLMLAIY MAAE------ --AGNPYFRL GYNSLGAFAT INHLHFQAYY LAVPFPIEKA PAKE-IGT-- ---------- ---------- ---------- -----LNGGM KISELVNYPV
SpGGP-3     DHGSFLLALH FAIE------ --AAEPFFRV GYNSLGAFAT INHLHFQAYF LAASFPVEKA PTRR-IMTMK ---------- ---------- ---------- ---GREDKGV IISQILNYPV
SmGGP-1     ETDTFLLAIY MAAE------ --ASSPFFRL GYNSLGAFAT INHLHFQAYY LGNCFPVEKA PTKL-ILH-- ---------- ---------- ---------- -----HYKGF KVYQLENYPV
SmGGP-2     EVNTFIIALQ MAKQ------ --ANNIYFRL GFNSLGAFAT INHLHFQAYY LEYIFPVEKA SKKL-LVN-- ---------- ---------- ---------- -----NVKGF NIYKLENYPV
SmGGP-3     CANDLLLALY FTAE------ --VDCPYFRV GYNSLGAFAT INHLHFQAYY LAYPFPVECA SITR-INK-- ---------- ---------- ---------- ---GSLKSSL RISELVDYPV
SiGGP-1     DPESFLLALQ MAAE------ --AASPYFRL GYNSLGAFAT INHLHFQAYY LSVPFPVEKA ATHK-IPLSE ---------- ---------- ---------- ---DTMKSGV TVSKLINYPV
SiGGP-2     DHESFLMAMY VARE------ --ARNPFFRV GYNSLGGFAT INHLHFQAYY LKVQYPVEKA TTEK-LTT-- ---------- ---------- ---------- -----LVNGV SIAQLVGYPV
SvGGP-1     DPESFLLALQ MAAE------ --AASPYFRL GYNSLGAFAT INHLHFQAYY LSVPFPVEKA ATHK-IPLSE ---------- ---------- ---------- ---DTMKSGV TVSKLINYPV
SvGGP-2     DHESFLMAMY VARE------ --ARNPFFRV GYNSLGGFAT INHLHFQAYY LKVQYPVEKA TTEK-LTT-- ---------- ---------- ---------- -----LVNGV SIAQLVGYPV
SlGGP-1     DRDSFLLALH MAAE------ --AANPYFRL GYNSLGAFAT INHLHFQAYF LAVQFPIEKA PTQK-ITV-- ---------- ---------- ---------- -----TDAGV KISEMLHYPV
```

HIT motif

```
SlGGP-2       DRDSFAIALH FARE------ --VADPFFRV GYNSLGAFAT INHLHFQAYY LSVPFPVEKA PIQK-ILARK ---------- ---------- ---------- ---GLGGAGV IVSKLLNYPV
StGGP-1       DRDSFLLALH MAAE------ --AANPYFRL GYNSLGAFAT INHLHFQAYF LAVQFPIEKA PTQK-ITV-- ---------- ---------- ---------- -----TDAGV KISEMLNYPV
StGGP-2       DRDSFAIALH FARE------ --VADPFFRV GYNSLGAFAT INHLHFQAYY LSVPFPVEKA PIQK-ILARK ---------- ---------- ---------- ---GLGGAGV IVSKLLNYPV
SbGGP-1       DPESFLLALQ MAAE------ --AASPYFRL GYNSLGAFAT INHLHFQAYY LSVPFPVEKA ATLK-IPLSE ---------- ---------- ---------- ---DTMKNGV TVSKLINYPV
SbGGP-2       DQESFLIAMY VARE------ --ARNPFFRV GYNSLGGFAT INHLHFQAYY LRVQYPVEKA TTEK-LTS-- ---------- ---------- ---------- -----LGNGV SIFHLLDYPV
SfGGP-1       DPDTFLLALQ MAVE------ --ADNPYFRL GYNSLGAFAT INHLHFQAYY LAAPFPVERA PTTL-ICH-- ---------- ---------- ---------- ---RWKKGGV KILELGNFPV
SfGGP-2       DPDTFLLALH MAAE------ --ADNPYFRL GYNSLG---- --------AYY LTAPFPVERA PTTP-ICH-- ---------- ---------- ---------- ---HCKKGGI KILELGNFPV
SppGGP-1      DRESFLLALH MTAE------ --AGSPYFRV GYNSLGAFAT INHLHFQAYF LAVPFPIEKA PTRR-IPLS- ---------- ---------- ---------- -----PKSGV KVSSLLNYPV
SppGGP-2      DSESFFLAVK MAAE------ --AGSPYFRV GYNSLGAFAT INHLHFQAYY LSVPFPVEKA PTKK-IARTK G--------- ---------- ---------- ---GPTKGGV QVSSLLNYPV
TcGGP-1       DRQSFLLALC MAAE------ --AGNPYFRL GYNSLGAFAT INHLHFQAYY LAVPFPIEKA PTRK-ITT-- ---------- ---------- ---------- -----LSDGV IISELLKYPV
TcGGP-2       DHDSFLLAIH LARE------ --AADPFFRV GYNSLGAFAT INHLHFQAYY LEVPFPIEKA PIQR-ISTKE ---------- ---------- ---------- ---GLQDTGV IVSQLLNYPV
TpGGP         DHESLLLALH MAAE------ --AANPYFRL GYNSLGAFAT INHLHFQAYY LAMPFPIEKA PTKK-IAM-- ---------- ---------- ---------- -----SNGGV KISKLLKYPV
VvGGP-1       DRESFLLALD MAVE------ --AGNPYFRL GYNSLGAFAT INHLHFQAYY LATPFPIEKA PTRK-ITT-- ---------- ---------- ---------- -----AGNGV KIFELLKYPV
VvGGP-2       DHDSFLLALH MAKE------ --AADPFFRL GYNSLGAFAT INHLHFQAYY LMAPFPVEKA PTER-IIRRG ---------- ---------- ---------- ---KLPNSGV MISKMLNYPV
VcGGP         SCGTVLLALQ FAGE------ --LGNSHFRV GYNSLGAYAT INHLHFQSYF LAKTMPCEAA ATVP-LPGVL AVPPVTGGGG GGSTAALLPL LRKRHFDDDA VSGVLAGAAV RVSRLVDYPV
ZmGGP-E-1     DPESFLLALQ MAAE------ --GGSPYFRL GYNSLGAFAT INHLHFQAYY LSVPFPVEKA ATHK-IPLSE ---------- ---------- ---------- ---DTKKNGV TVSKLINYPV
ZmGGP-E-2     DQESFLIAMY VARE------ --ARTPFFRV GYNSLGGFAT INHLHFQAYY LKVQYPVEKA ITEK-LKT-- ---------- ---------- ---------- -----LGNSV SIFHLVDYPV
ZmGGP-1       DPESFLLALQ MAAE------ --GGSPYFRL GYNSLGAFAT INHLHFQAYY LSVPFPVEKA ATHK-IPLSE ---------- ---------- ---------- ---DTKKNGV TVSKLINYPV
ZmGGP-2       DQESFLIAMY VARE------ --ARTPFFRV GYNSLGGFAT INHLHFQAYY LKVQYPVEKA ITEK-LKT-- ---------- ---------- ---------- -----LGNSV SIFHLVDYPV
ZomGGP        DSESFLLAVH MASE------ --AASPYFRL GYNSLGAFAT INHLHFQAYY LSVAFPVEKS PTKP-ISISK S--------- ---------- ---------- ---SNKSIGV TVSQLFGYPV
                                                     HIT motif

              ....|....| ....|....| ....|....| ....|....| ....|....| ....|....| ....|....| ....|....| ....|....| ....|....| ....|....| ....|....|
                      850        860        870        880        890        900        910        920        930        940        950        960
AcGGP         RGLVFE---- --GGN----S LE-------- --------DL SNAVSDSSIC LQGNNIPYNV LISDSGKCIF LLP------- ---QCYAEKQ ALGEVSSDLL DTQVNPAVWE ISGHMVLKRK
AcGGP-R-1     RGLVFE---- --GGN----T LE-------- --------DL SNAVSDSSIC LQGNNIPYNV LISDSGKRIF LLP------- ---QCYAEKQ ALGEVSSELL DTQVNPAVWE ISGHMVLKRK
AcGGP-R-2     RGLVFE---- --GGT----N LR-------- --------NL SNAVANACIC LQKKNIPFNV LIADCGKRIF LFP------- ---QCYAGKQ ALGEVSEELM ETQVNPAVWE ISGHMVLKRR
AdGGP         RGLVFE---- --GGN----T LE-------- --------DL SNAVSDSSIC LQGNNIPYNV LISDSGKRIF LLP------- ---QCYAEKQ ALGEVSSELL DTQVNPAVWE ISGHMVLKRK
AeGGP         RGLVFE---- --GGN----S LE-------- --------DL SNAVSDSSIC LQCNNIPYNV LISDSGKRIF LLP------- ---QCYAEKQ ALGEVSSELL DTQVNPAVWE ISGHMVLKRK
ArGGP         RGLVFE---- --GGN----S LE-------- --------DL SNAISDSSIC LQGNNIPYNV LISDSGKRIF LLP------- ---QCYAEKQ ALGEVSPDLL DTQVNPAVWE ISGHMVLKRK
AmhGGP-1      RGLVLE---- --GGN----S LQ-------- --------DL AKVVSDCCVF LQDTNIPFNV LIAESGKRIF LLP------- ---QCYAEKQ ALGEVNPELL DTQVNPAVWE ISGHIVLKRR
AmhGGP-2      RGFVFE---- --GGK----T LK-------- --------DL SDAVASACIR LQNTNFPFNV LISGCGKKVF LFP------- ---QCYAEKQ ALGQVSQEVL NTQVNPAVWE ISGHIVLKRR
AncGGP-3      RGLVFE---- --GGK----N ML-------- --------DL SDTVSSACIF LQESNRPFNV LFSECGKRIF LLL------- ---QCYAMKQ AHGKVSPEIL ETRVNPACWE LGGHFALKRR
AncGGP-1      RGLVYE---- --GGN----T LK-------- --------EL SDVVANACIC LQDNNIPFNV LISDAGRRIF LFP------- ---QCYAEKQ ALGEVSQELL DTQVNPAVWE ISGHMVLKRK
AncGGP-2      RGLVFE---- --CGN----T MK-------- --------DL SNAVANSCIY LQENNIPFNV LICRSGGRIF LFP------- ---QCYAEKQ ALGKVSQELL DTQVNPAAWE IGGHMVLKRR
AqcGGP        RGLVFE---- --GGI----S IE-------- --------DL SIAVSDSCIC LQENNIPYNV LIADSGKRTF LLP------- ---QCYAEKQ ALGEVSPELL DTQVNPAVWE ISGHMVLKRK
AhGGP-1       RSLLFE---- --GGS----S MQ-------- --------EL SDTVSDACVC LQNNNIPFNI LISDCGRQIF LMP------- ---QCYAEKQ ALGEVSPEVL ETQVNPAVWE ISGHMVLKRK
AhGGP-2       RGLLFE---- --GGN----S IK-------- --------DL SDTVSDASVC LQNNNIPFNI LISDSGKQIF LLP------- ---QCYAEKQ ALGEVSSKLL DTQVNPAVWE MSGHMVLKRK
AlGGP-1       RSLLFE---- --GGS----S MQ-------- --------EL SDTVSDACVC LQNNNIPFNI LISDCGRQIF LMP------- ---QCYAEKQ ALGEVSPEVL ETQVNPAVWE ISGHMVLKRK
AlGGP-2       RGLLFE---- --GGN----S IK-------- --------DL SDTVSDASVC LQNNNIPFNI LISDSGKQIF LLP------- ---QCYAEKQ ALGEVSSKLL DTQVNPAVWE MSGHMVLKRK
AtGGP-1       RSLLFE---- --GGS----S MQ-------- --------EL SDTVSDCCVC LQNNNIPFNI LISDCGRQIF LMP------- ---QCYAEKQ ALGEVSPEVL ETQVNPAVWE ISGHMVLKRK
AtGGP-2       RGLLVE---- --GGN----T IK-------- --------DL ADTVSDASVC LQNNNIPFNI LISDSGKRIF LLP------- ---QCYAEKQ ALGEVSSTLL DTQVNPAVWE MSGHMVLKRK
BosGGP-1      RSLLFE---- --GGS----S MQ-------- --------DL SDTVSDACVC LQNNNIPFNI LISDCGRQIF VMP------- ---QCYAEKQ ALGEVSPKVL QTQVNPAVWE ISGHMVLKRK
BosGGP-2      RGLRFE---- --GGH----S IK-------- --------DL SDTVSDASIC LQNNNAPFNI LISDSGKQIF LLP------- ---QCYAEKQ ALGEVSSKLL DTQVNPAVWE MSGHMVLKRK
BdGGP-1       RGLVFE---- --GGN----T LN-------- --------DL ADVVSGACIW LQENNVPYNV LISDCGRKVF LFP------- ---QCYAEKQ ALGEVSQELL DTQVNPAVWE ISGHIVLKRR
BdGGP-2       RGLVFE---- --RGN----T LK-------- --------DL GDVVTNACIW LQDNNVPFNV LISDSGQKIF IFP------- ---QCYAEKQ ALGEVSQDLL DTQVNPAVWE ISGHIVLKRR
BdGGP-3       SGFVFE---- --GEV----S LE-------- --------DL SYVVSNACTF MQDNNRPFNV LISESAKRVF LLP------- ---QCYAEKQ ALGKASQEFL DMRINPAVWE LSGHLVLKRR
BsGGP-1       RGLVFE---- --GGN----T LN-------- --------DL ADVVSGACIW LQDNNVPYNV LISDCGRKVF VFP------- ---QCYAEKQ ALGEVSQELL DTQVNPAVWE ISGHIVLKRR
BsGGP-2       RGLVFE---- --RGN----T LK-------- --------DL GDVVTNACIW LQDNNVPFNV LISDSGRKIF IFP------- ---QCYAEKQ ALGEVSQDLL DTQVNPAVWE ISGHIVLKRR
BsGGP-3       SGFVFE---- --GGT----S LE-------- --------DL SHVVSNACIF LQENNRPFNV LISESATRVF LLP------- ---QCYAEKQ ALGKASQEFL DMRINPAVWE LSGHLVLKRR
BoGGP-1       RSLLFE---- --GGS----S MQ-------- --------DL SDTVSDACVC LQNNNIPFNI LIADCGRQIF LMP------- ---QCYAEKQ ALGEVSPEVL ETQVNPAVWE ISGHMVLKRK
BoGGP-2       RGLLFE---- --GGN----S MQ-------- --------DL SDTVSDACVC LQNNNVPFNV LISDCGRRIF LMP------- ---QCYAEKQ ALGEVSPEVF ESQVNPAVWE ISGHMVLKRK
BoGGP-3       RGLLFE---- --GGN----S IK-------- --------DL SVAVSNASVC LQNNNIPFNF LISDSGKRIF LLL------- ---QCYAEKQ ALGEVSSELL DTQVNPAVWE MSGHMVLKRK
BrGGP-1       RSLLFE---- --GGS----S MQ-------- --------DL SDTVSDACVC LQNNNIPFNI LIADCGRQIF LMP------- ---QCYAEKQ ALGEVSPEVL ETQVNPAVWE ISGHMVLKRK
BrGGP-2       RSLLFE---- --GGN----S MQ-------- --------DL SDTVSDACVC LQENNIPFNI LISDCGRQIF LMP------- ---QCYAEKQ ALGEVSPELL ETQVNPAVWE ISGHMVLKRK
BrGGP-3       RGLLFE---- --GGN----S MQ-------- --------DL SDTVSDACVC LQNNNVPFNV LISDRGRQIF LMP------- ---QCYAEKQ ALGEVSTEVW EAQVNPAVWE ISGHMVLKRK
BrGGP-4       RGLLFE---- --GGN----S IK-------- --------EL SDAVSNASVC LQNNNISFNI LISDSGKRIF LLP------- ---QCYAEKQ ALGGVSSELL DTQVNPAVWE MSGHMVLKRK
CgGGP-1       RSLLFE---- --GGS----S MQ-------- --------DL SDTVSDACVC LQNNNIPFNI LIADCGRQIF VMP------- ---QCYAEKQ ALGEVSPEVL ETQVNPAVWE ISGHMVLKRK
CgGGP-2       RGLLFE---- --GGN----S IK-------- --------DL SDTVSDASVC LQNNNIPFNI LISDSGRQIF LLP------- ---QCYAEKQ ALGEVCSKLL DTQVNPAVWE MSGHMVLKRK
CrGGP-1       RSLLFE---- --GGS----S MQ-------- --------DL SDTVSDACVC LQNNNIPFNI LIADCGRQIF VMP------- ---QCYAEKQ ALGEVSPEVL ETQVNPAVWE ISGHMVLKRK
CrGGP-2       RGLLFE---- --GGN----S IK-------- --------DL SDTVSDASVC LQNNNIPFNI LISDSGRQIF LLP------- ---QCYAEKQ ALGEVCSKLL DTQVNPAVWE MSGHMVLKRK
ChrGGP        RSFVVE---- AEAGA----A LE-------- --------AV AAVVARAADA MQAANQPFNI IASDGGRRVF LFP------- ---QCYAERQ AAGEVPEELL DTGVNPASFE IAGHLVLKRA
CcGGP-1       RGLVFE---- --GGN----S LE-------- --------DL SNTVSDACIC LQENNIPYNV LIADCGKRIF LLP------- ---QCYAEKQ ALGEVSSELL DTQVNPAVWE ISGHMVLKRK
CcGGP-2       RGLVFE---- --VGK----S LR-------- --------DL SDAVASSCIC LQDNNIPFNV LISDCGRRII LFP------- ---QCYAEKQ ALGEVSQELL DTQVNPAAWE ISGHIVLKRR
CsGGP-1       RGLVFE---- --GGN----S LE-------- --------DL SNTVSDACIC LQENNIPYNV LIADCGKRIF LLP------- ---QCYAEKQ ALGEVSSELL DTQVNPAVWE ISGHMVLKRK
CsGGP-2       RGLVFE---- --VGK----S LR-------- --------DL SHAVASSCIC LQNNNIPFNV LISDCGRRII LFP------- ---QCYAEKQ ALGEVSQELL DTQVNPAAWE ISGHIVLKRR
CosGGP        CGLVFE---- --ASD----S ID-------- --------EL ASVVGEACQR LSAANIPHNL FVVDCGQRIF LFP------- ---NAFARAK AKGLVPEDLL DSQVDPAAFE ISGHIIYKRS
CusGGP        RGLVFE---- --GGN----T LQ-------- --------SL SNSVSDACIC LQENNIPYNI LIADCGQRIF LLP------- ---QCYAEKQ ALGEVSAELL DTQVNPAVWE ISGHMVLKRK
DcGGP-1       RGLVFE---- --GLC----T LE-------- --------DL STAVSDACIC LQNSNIPYNV LISDSGRRVF LLP------- ---QCYAERQ ALGEVSPELL DTQVNPAVWE ISGHMVLKRK
DcGGP-2       RGLVFE---- ---GS----S LE-------- --------NL SAAVADACIC LQINNIPYNV LISDSGRRIF LLP------- ---QCYAEKQ ALGEVSSELL DTQVNPAVWE ISGHMVLKRK
DsGGP         RGFAIE---- --VLSSAVGA LE-------- --------EA AELVGYMVST MQRADIPHNV LITDSGMRIF IWP------- ---QRYAEAQ ALGQVPAEQL DTGVNPAVFE IAGHMVLKRA
EgGGP-1       RGLVYE---- --GGN----T IQ-------- --------DL ANAVSDSCIC LQDNNIPYNV LISDCGKRIF LFP------- ---QCYAEKQ ALGEVDAEVL DTQVNPAVWE ISGHMVLKRK
EgGGP-2       RGLVFE---- --GGD----T VQ-------- --------DL SNAVSDSCIC LQDNNIPYNV LISDCGKRIF LLP------- ---QCYAEKQ ALGEVNSELL DTQVNPAVWE ISGHIVLKRK
EgGGP-3       RSLVFE---- --GRD----T LW-------- --------DL SSAIANSCIR LQHNNIPFNV LIADCGKRVF LFP------- ---QCYAKKQ ARGEVSQELL ETQVNPAIWE IGGHIVLKRK
EgGGP-4       RSLVFE---- --GGD----T LW-------- --------DL SSAVANSCIL LQYNNIPFNI LIADCGKRVF LFP------- ---QCYAEKQ ARGEVSQELL ETQVNPAVWE IGGHIVLKRK
EgGGP-5       RSLVFE---- --GRD----T LW-------- --------DL SSAVANSFIR LQHNNIPFNV LIADCGKRVF LFP------- ---QCYAEKQ ARGEVSQELL ETQVNPVVWE IGGHIVLKRR
EsGGP-1       RSLLFE---- --GGN----S MQ-------- --------DL SDTVSDACVC LQNNNIPFNI LISDCGRQIF LMP------- ---QCYAEKQ ALGEVSPEVL ETQVNPAVWE ISGHMVLKRK
EsGGP-2       RGLLFE---- --GGS----S IK-------- --------DL SDTVANASVC LQNNNIPFNI LISDSGKQIF LLP------- ---QCYAEKQ ALGEVSSKLL DTQVNPAVWE MSGHMVLKRK
FvGGP-1       RGLVFE---- --GGN----T LE-------- --------DL SNSVSDACIC LQENNIPYNV LISDCGKRIF LLP------- ---QCYAEKQ ALGEVRAELL DTQVNPAVWE ISGHMVLKRK
FvGGP-2       RGLVFE---- --GGN----T LE-------- --------DL SNSVSDACIC LQENNIPYNV LISDCGKRIF LLP------- ---QCYAEKQ ALGEVRAELL DTQVNPAVWE ISGHMVLKRK
FvGGP-3       RGLVFE---- --GGN----T LE-------- --------DL SNSVSDACIC LQENNIAYNV LISDFGNRIF LLP------- ---QCYAEKQ ALGEVRAELL DTQVNPAVWE ISGHMVLKRK
GmGGP-1       RGFVFE---- --GGE----M LE-------- --------DL SNVVSDACIC LQNNNIPFNV LISDCGKQVY LLP------- ---QCYAEKQ ALGEVNAELL DTQVNPAVWE ISGHMVLKRR
GmGGP-2       RGLVFE---- --GGD----T LE-------- --------DL SNVVSDACIC LQNNNIPFNV LISDCGKQVY LLP------- ---QCYAEKQ ALGEVDAELL DTQVNPAVWE ISGHMVLKRK
GmGGP-3       RGLVFE---- --GGH----T LE-------- --------DL ANAVSEACIC LQHNNIPYNV LISDCGRQIF LLP------- ---QCYAEKQ ALGEVSPELL ETQVNPAVWE ISGHMVLKRK
GmGGP-4       RGLVFE---- --GGH----S LE-------- --------GL ANVVSEACIC LQLNNIPYNI LISDCGRQIF LLP------- ---QCYAEKQ ALGEVSAELL ETQVNPAVWE ISGHLVLKRE
GnmGGP        RGLVFE---- --GGC----S LD-------- --------DL SNVVANSCLY LQDNNIPFNV LIADCGKRIF LFP------- ---QCYAEKQ ALGEVDQEIL DTQVNPAVWE ISGHMVLKRK
GrGGP-1       KGLVFD---- --GGN----T LQ-------- --------DL SDAVSDACIC LQDNNIPYNV LISDCGKRIF LLP------- ---QCYAEKQ ALGEVSPELL DTQVNPAVWE ISGHMVLKRK
GrGGP-2       RGLVFE---- --GGN----T LQ-------- --------AL SDTVSDACIC LQENNIPYNV LISDCGKRIF LVP------- ---QCYAEKQ ALGEVSPELL DTQVNPAVWE ISGHMVLKRR
GrGGP-3       RGVVFE---- --GGN----T LQ-------- --------DL SDAVSDACIC LQDNNIAYNV LISDCGKRIF LLP------- ---QCYAEKQ ALGEVSSELL DTQVNPAVWE ISGHMVLKRK
KfGGP-1       RGLVFE---- --GGQ----T LD-------- --------DL SKAVSDSCIC LQNNNIPHNV LISDCGKRIF LLP------- ---QCYAEKQ ALGEVSAELL DTQVNPAVWE ISGHMVLKRK
KfGGP-2       RGLVFE---- --GGQ----T LE-------- --------DL SKAVSDSCIC LQNNNIPHNV LISDSGKRIF LLP------- ---QCYAEKQ ALGEVSAELL DTQVNPAVWE ISGHMVLKRK
KlGGP-1       RGLVFE---- --GGQ----T LD-------- --------DL SKAVSDSCIC LQNNNIPHNV LISDCGKRIF LLP------- ---QCYAEKQ ALGEVSAELL DTQVNPAVWE ISGHMVLKRK
KlGGP-2       RGLVFE---- --GGQ----T LE-------- --------DL SKAVSDSCIC LQNNNIPHNV LISDSGKRIF LLP------- ---QCYAEKQ ALGEVSAELL DTQVNPAVWE ISGHMVLKRK
KlGGP-3       RGLVFE---- --GGQ----T LE-------- --------DL SKAVSDSCIC LQNNNIPHNV LISDSGKRIF LLP------- ---QCYAEKQ ALGEVSAELL DTQVNPAVWE ISGHMVLKRK
LuGGP         RGLVFE---- --GGN----T LQ-------- --------DL SNTVSDACIC LQDNNVPHNV LIADCGKRVF LLP------- ---QCYAEKQ ALGEVSAELL DTQVNPAVWE ISGHMVLKRK
MdGGP         RGLVFE---- --GGN----T LE-------- --------DL SYTVSDACIC LQENNVPYNV LISDCGKRIF LLP------- ---QCYAEKQ ALGEVSAEVL DTQVNPAVWE ISGHMVLKRK
MeGGP-1       RSLLFE---- --GGN----T LQ-------- --------DL SNTVSDACIC LQDNSIPYNV LISDCGNRIF LLP------- ---QCYAEKQ ALGEVSPELL HTQVNPAVWE ISGHMVLKRK
MeGGP-2       RGLLFE---- --GGN----T LQ-------- --------DL SNTVSDACIC LQDNNIPYNV LISDCGKRIF LLP------- ---QCYAEKQ ALGEVSPELL DTQVNPAVWE ISGHMVLKRK
MeGGP-3       RGFVFE---- --GGN----T MQ-------- --------CL SDSVASACIY LQNNNIPYNV LIADSGNKIF LFP------- ---QCFAEKQ ALGKVSQELL ETLVNPAVWE IAGHIVLKRR
MpGGP         RTIVYD---- --VGE----S LE-------- --------EF ADIIANACIR LQNDNIPFNV LIADCGARVF LIP------- ---QCYAERQ ARGEVDQAVL DTQVNPAVWE ISGHIVLKRR
MtGGP-1       RGLVFE---- --GGD----T LD-------- --------DL SKIVSDACIS LQNNNIPYNV LISDCGTQVF LLP------- ---QCYAEKQ ALGEVDAELL DTQVNPAVWE ISGHMVLKRK
MtGGP-2       RGLVFE---- --GGH----A LD-------- --------DL ANTVSEACIC LQHNNIPYNV LISDCGRRVF LLP------- ---QCYAEKQ ALGEVRAELL DTQVNPAVWE ISGHMVLKRK
MpGGP         RCLCFERADP SRGSD----G FE-------- --------SL AASLAVCCER LQARDVPFNL LVADHGARVF LIP------- ---NQFSQRA AKGALPADVV STGVNPAVFE ISGHLLYKQR
MsGGP         RCLCFE---- --RKD----AT FE-------- --------AL ADLLGNACER LQERNIPFNL LIADHGARVF LLP------- ---QVFSHRV AKGEIPEDVA ATGVNPAVFE ISGHLLYKQQ
MgGGP-1       RGLVFE---- --HGD----N IE-------- --------DL SNAVSESCIC LQENNIPHNV LIADSGKRVF LFP------- ---QCYAEKQ ALGEVSAELL DTQVNPAVWE ISGHMVLKRK
MgGGP-2       RGLVFE---- --GGN----T LE-------- --------DL SNIVSNSCIC LQENNIPYNV LIADCGKRVF LFP------- ---QCYAEKQ ALGEVSSELL DTQVNPAVWE ISGHMVLKRK
MaGGP-1       RGLVYE---- --GGN----T LK-------- --------DL SDVVANSCIC LQENNIPFNV LISDSGRRIF LFP------- ---QCYAEKQ ALGEVSQEIL DTQVNPAVWE ISGHMVLKRK
MaGGP-2       RGLVYE---- --GGK----H LK-------- --------DL ADMVANSCIW LQSNNIPFNV LISDSGRRVF LFP------- ---QCYAEKQ ALGEVGQELL DTQVNPAVWE ISGHMVLKRK
MaGGP-3       RGLVYE---- --GGN----T LK-------- --------DL SDVVANSCIC LQENNIPFNV LISDSGRRIF LFP------- ---QCYAEKQ ALGEVGQDLL DTQVNPAVWE ISGHIVLKRK
MaGGP-4       RGLVFG---- --GGS----N LE-------- --------DL SIAVSIACIF LQDNDIPYNV IISDAGKRIF LLL------- ---QCYAEKR TRGEVNQEFL EMQINPAAWE LGGYMVVKRR
OtGGP         RGLVFE---- --GGN----T LN-------- --------DL ANVVSSACIW LQDNNVPYNV LISDCGKRIF LFP------- ---QCYAEKQ ALGEVSQELL DTQVNPAAWE ISGHIVLKRR
OsGGP-1       RGLVFE---- --GGN----S LS-------- --------DL ANVVSSACIW LQDNNVPYNV LISDCGKKIF LFP------- ---QCYAEKQ ALGEVSQELL DTQVNPAVWE ISGHIVLKRR
OsGGP-2       SGFVFE---- --GGA----C LE-------- --------DL SDVVSKVCIF LQENNKPFNA LISESGKRVF LLP------- ---QCYAEKQ ALGRVSQEFL DMRINPAVWE LSGHLVLKRR
OlGGP         RCLCFE---- --VGD----S FE-------- --------EL ASLVGGCAVK LQGANIPFNL LIADHGARVF LIP------- ---QVFSIRI ANNKIPEHVI HTGVNPAVFE ISGHLLYKQE
PhGGP-1       RGLVFE---- --GGN----T LN-------- --------DL ANVVSSACIW LQDNNVPYNV LISDGGKRIF LFP------- ---QCYAEKQ ALGEVSQELL DTQVNPAVWE ISGHIVLKRR
PhGGP-2       SGFVFE---- --GGA----S LE-------- --------DL SDVVAKVCIF LQENNRPFNV LISESGNSVF LLP------- ---QCYAEKQ RLGKASQEFL DMRINPAIWE LSGHLVLKRR
PvGGP-1       RGLVFE---- --AGN----T LN-------- --------DL ANAVSSACIW LQDNNVPYNV LISDCGKRIF LFP------- ---QCYAEKQ ALGEVSQELL DTQVNPAVWE ISGHIVLKRR
```

```
PvGGP-2       RGLVFE---- --GGS----T LN-------- --------DL ANVVSSACIW LQDNNVPYNV LISDCGKRIF LFP------- ---QCYAEKQ ALGEVSQELL DTQVNPAVWE ISGHIVLKRR
PvGGP-3       SGFVFE---- --GGA----S LL-------- --------YL SDVVARVCIF LQENNRPFNV LISESGNRVF LLP------- ---QCYAEKQ LLGKASQEFL EMRINPAIWE LSGHLVLKRK
PhvGGP-1      RGLVFE---- --GGD----T VE-------- --------DL SNVVSDACIC LQNNNIPFNV LISDCGKQVF LLP------- ---QCYAEKQ ALGEVDAELL DTQVNPAVWE ISGHMVLKRK
PhvGGP-2      RGLVFE---- --GGH----T LE-------- --------NL ANAVSEACIC LQHNNVPYNV LISDCGRQIF LLP------- ---QCYAEKQ ALGEVSAELL ETQVNPAVWE ISGHMVLKRK
PpGGP-1       RGLVFE---- --MCN----S MV-------- --------DL SIAVANACIY LQNENIPYNV LITDRGSRVF LLP------- ---QCFAERQ ARGEVDEDIL ETQVNPAVWE ISGHIVLKRR
PpGGP-2       RGLVFE---- --ICN----S ME-------- --------DL SEAVANACIY LQDNNIPYNV LIADRGSRVF LLP------- ---QCFAERQ ARGEVDQEIL DTQVNPAVWE ISGHIVLKRR
PpGGP-3       RGLVFE---- --VCT----S LE-------- --------DL SIAVGNACVY LQNENIPYNV LIADRGSRVF LLP------- ---QYFAERQ ARGEVDQEIL ETQVNPAVWE ISGHIVLKRR
PaGGP-1       RGLVFE---- --GCN----T LE-------- --------NL SNAVGSACIY LQDNNIPYNV LIADCGKRVF LFP------- ---QCYAEKQ ALGEVDQEIL DTQVNPAVWE ISGHIVLKRK
PaGGP-2       RGLVFE---- --GGN----T LE-------- --------DL SNAVGSACIC LQDNNIPYNV LIADCGKRIF LFP------- ---QCYAEKQ ALGEVDQEIL DTQVNPAVWE ISGHIVLKRK
PaGGP-3       RGLVFE---- --GGN----T LE-------- --------DL SNAVGSSCIC LQDNNIPYNV LVADCGKRVF LFP------- ---QCYAEKQ ALGEVDQEIL DTQVNPAVWE ISGHMVLKRK
PsGGP-1       RGLVFE---- --GCN----T LE-------- --------NL SNAVGSACIC LQDNNIPYNV LIADCGKRVF LFP------- ---QCYAEKQ ALGEVDQEIL DTQVNPAVWE ISGHIVLKRK
PsGGP-2       RGLVFE---- --GGN----T LE-------- --------DL CNAVGSSCIC LQDNNIPYNV LIADCGKRVF LFP------- ---QCYAEKQ ALGEVNQGIL DTQVNPAVWE ISGHIVLKRK
PsGGP-3       RGLVFE---- --GGN----T LE-------- --------DL SNAVGSSCIC LQDNNIPYNV LVADCGKRVF LFP------- ---QCYAEKQ ALGEVDQEIL DTQVNPAVWE ISGHMVLKRK
PtGGP-1       RGIVFE---- --GGN----T LD-------- --------NL SNAVGNACIC LQDNNIPYNM LIADCGKRVF LFP------- ---QCYAEKQ ALGEVDQEIL DTQVNPAVWE ISGHIVLKRK
PtGGP-2       RGLVFE---- --GGN----T LE-------- --------DL CNAVGSSCIC LQDNNIPYNV LIADCGKRVF LFP------- ---QCYAEKQ ALGEVNQEIL DTQVNPAVWE ISGHMVLKRK
PtGGP-3       RGLVFE---- --GGN----T LK-------- --------DL SNAVGSACIC LQDNNIPYNF LIANCGKKVF LFP------- ---QCYAEKQ ALGEVDQEIL DTQVNPAVWE ISGHIVLKRK
PtGGP-4       RGLVFE---- --GGN----T LE-------- --------DL CNAVGSSCIC LQDNNIPYNV LIADCGKRVF LFP------- ---QCYAEKQ ALGEVDQEIL DTQVNPAVWE ISGHIVLKRK
PtGGP-5       RGLVFE---- --GGN----T LE-------- --------DL CNAVGSSCIR LQDNNIPYNV LIGDCGKRVF LFP------- ---QCYAEKQ ALGEVNQEIL DTQVYPGAWE ISGHMALKWR
PotGGP-1      RGLVFE---- --GGN----A LL-------- --------DL SNGVSDACIC LQENNIPYNV LIADCGNRIF LLP------- ---QCYAEKQ ALGEVSPELL DTQVNPAVWE ISGHMVLKRK
PotGGP-2      RGLFFE---- --GGN----A LQ-------- --------DL SNTVSDACIC LQENNIPYNV LIADCGNHIF LLP------- ---QCYAEKQ ALGEVSSELL DTQVNPAVWE ISGHMVLKRK
PotGGP-3      RGLVFE---- --GGS----T VQ-------- --------DL SDSVASSCIF LQNNNIPFNV LITDCGRRIF LFP------- ---QCYAEKQ ARGEASQELL DTQVNPAVWE ISGHIVLKRQ
PrpGGP        RGLVFE---- --GGN----T LQ-------- --------DL SNTVSDACIC LQENNIPYNV LISDCGKRIF LLP------- ---QCYAEKQ ALGEVRAELL DTQVNPAVWE ISGHMVLKRK
RcGGP-1       RGLLFE---- --DGN----T LQ-------- --------DL SSTISDACIC LQDNSIPYNV LISDCGKRLF LLP------- ---QCYAEKQ ALGEVSPELL ETQVNPAVWE ISGHMVLKRK
RcGGP-2       RGLLFE---- --GGN----T LQ-------- --------DL SNTISDACIC LQDNNIPYNV LISDCGKCVF LLP------- ---QCYAEKQ ALGEVSAELL DTQVNPAVWE ISGHMVLKRK
RcGGP-3       RGLVFE---- --GGN----T MQ-------- --------DL SDSVASSCIY LQNNNVAFNV LIAECGRRVF LFPQIFLSVS ANVQCYAEKQ ALGEVSQELL DIQVNPAVWE ISGHIVLKRR
SpGGP-1       RGLVFE---- --GGN----A LQ-------- --------DL SNTVSDACIC LQENNIPYNV LIADCGNRIF VLP------- ---QCYAEKQ ALGEVSSELL DTQVNPAVWE ISGHMVLKRK
SpGGP-2       RGLVFE---- --GGN----T LL-------- --------DL SRGVSDACIC LQENNIPYNV LIADCGNRIF LLP------- ---QCYAEKQ ALGEVSSELL DTQVNPAVWE ISGHMVLKRK
SpGGP-3       RGLVFE---- --GGN----T IQ-------- --------DL SDSVASSCIF LQNNNIPFNV LIADCGRLIF LFP------- ---QCYAEKQ ARGETIQELL DTQVNPAVWE ISGHIVLKRQ
SmGGP-1       KGIVYE---- --VGE---SN FE-------- --------EL AFYIIKVCKG LQGRNIPYNI LIANKGCQIF LFP------- ---QCYAEKQ ARGEVDLEIL ETQVNPAVWE ISGHIVLKRK
SmGGP-2       RGIIYE---- --LGS---SN FQ-------- --------EL SYQIVTVCNI LEEQNIPYNI LIADKGSQIY LFP------- ---QCFAERQ VRGEVEAEIL ETQVNPAVWE ISGHIVLKRK
SmGGP-3       KGVVYE---- --SMD----- LN-------- --------EI AISVASTCQE LELRNIPFNL LICDCGTRVF LFP------- ---QCFARKQ ALHQVSQKVL DTQVNPAAWE IGGHIVLKRK
SiGGP-1       RGLVFE---- --GGN----T LN-------- --------DL ANVVSSACIW LQDNNVPYNV LISDCGKRIF LFP------- ---QCYAEKQ AMGEVSQELL DTQVNPAVWE ISGHIVLKRR
SiGGP-2       SGFVFE---- --GGA----S LE-------- --------DL SCMVSKVCIF LQENNRPFNV LISESGRRVF LLP------- ---QCYAEKQ LLGKASQEFL EMRINPAIWE LSGHLVLKRK
SvGGP-1       RGLVFE---- --GGN----T LN-------- --------DL ANVVSSACIW LQDNNVPYNV LISDCGKRIF LFP------- ---QCYAEKQ ALGEVSQELL DTQVNPAVWE ISGHIVLKRR
SvGGP-2       SGFVFE---- --GGA----S LE-------- --------DL SCMVSKVCIF LQENNRPFNV LISESGRRVF LLP------- ---QCYAEKQ LLGKASQEFL EMRINPAIWE LSGHLVLKRK
SlGGP-1       RGLVFE---- --GGN----T LE-------- --------DL ADVVSDSCIC LQENNIPYNV LISDSGKRIF LLP------- ---QCYAEKQ ALGEVSAELL DTQVNPAVWE ISGHMVLKRK
SlGGP-2       RGFAFE---- --GGNG--ST AR-------- --------DL SDAVVNSCIS LQNKNIPFNI LIAQCGKKIF LLP------- ---QCYAEKQ ALGVVDQELL DTQVNPAVWE ISGHIVLKRT
StGGP-1       RGLVFE---- --GGN----T LE-------- --------DL ANVVSDSCIC LQENNIPYNV LISDSGKRIF LLP------- ---QCYAEKQ ALGEVSAELL DTQVNPAVWE ISGHMVLKRK
StGGP-2       RGFAFE---- --GGNG--ST AR-------- --------DL SDAVVNSCIS LQNKNIPFNI LIAQCGKKIF LLP------- ---QCYAEKQ ALGVVDQELL DTQVNPAVWE ISGHIVLKRT
SbGGP-1       RGLVFE---- --GGN----T LE-------- --------DL ANVVSNACIW LQDNNVPYNV LISDCGKRVF LFP------- ---QCYAEKQ ALGEVSQELL DTQVNPAVWE ISGHIVLKRR
SbGGP-2       SGFLFK---- --GGA----S LE-------- --------DL SDVVSKVCIF LQENNRPFNV LISESGKRVF LLP------- ---QCYAEKQ ILGKASQEFL DMRINPAIWE LSGHLVLKRK
SfGGP-1       RGLVFE---- --VGD----S LE-------- --------DL ANAVANACIY LQNANIPFNV LIADRGGRVF IFP------- ---QCFAERQ ARGEVEQEIL DTQVNPAVWE ISGHIVLKRR
SfGGP-2       RGLVFE---- --IGD----N LE-------- --------DL ANAVANACIY LQNTNIPFNV LIADRGGRVF IFP------- ---QCFAEKQ ARGEVDQEIL DTQVNPAVWE ISGHIVLKRR
SppGGP-1      RGLVFE---- --GGS----S LQ-------- --------EL SDLVADACIC LQESNTPFNV LISDMGRRIF LFP------- ---QCYAEKQ ALGEVDREIL ETQVNPAAWE ISGHIVLKRK
SppGGP-2      RGLVFE---- --GGS----D LQ-------- --------AL SDLVSNACVF LQENNTPFNV LISDSGKRIF VFP------- ---QCYAEKQ ALGEVSQELL ETQVNPAAWE ISGHMVLKRK
TcGGP-1       RGLVFE---- --GGN----T IQ-------- --------DL SDTVSDACIC LQDNNIPYNV LISDCGKRIF LLP------- ---QCYAEKQ ALGEVSPELL DTQVNPAVWE ISGHMVLKRR
TcGGP-2       RGLVFK---- --GGK----S AQ-------- --------DL SDVVANSCIF LQTNNIPFNV LISDCGKRVF LLP------- ---QCYAEKQ AIGEVSLELL HTQVNPAVWE IGGHMVLKRK
TpGGP         RGLVFE---- --GGD----T LE-------- --------DL SKIVSDACIS LQNNNIPYNV LISDCGTQVF LLP------- ---QCYAEKQ ALGEVDAELL DTQVNPAVWE ISGHMVLKRK
VvGGP-1       RGLVFE---- --GGD----T LQ-------- --------DL ANTVADSCIC LQDNNIPFNV LIADAGKRIF LFA------- ---QCYAEKQ ALGEVNQELL DTQVNPAVWE VSGHIVLKRK
VvGGP-2       QGFVFE---- --GGN----L MQ-------- --------DL SDTVANSCIF LQNNNIPYNV LISDCGGRIF LFP------- ---QCFAEKQ ALGEVSQELL DTLVNPAVWE ISGHMVLKRK
VcGGP         NAFVIE---- --ALT----S LETSLYDCGC GSPSSATAAV AAVVGLAAER LQAANQPFNL LISDAGRRVF LFP------- ---QCFAERQ AAGLIPPELL ETGVNPAAFE IAGHLVLKRT
ZmGGP-E-1     RGLVFE---- --GGN----T LD-------- --------DL ATVVSNACIW LQDNNVPYNV LISDCGKRVF LFP------- ---QCYAEKQ ALGEVSQELL DTQVNPAVWE ISGHMVLKRR
ZmGGP-E-2     NGFVFE---- --GGA----S LE-------- --------DL SDVVSKVCIF LQENNRPFNV LISESGKRIF LLP------- ---QCYAEKQ LLGKASQEFL DMRINPAIWE LSGHLVLKRR
ZmGGP-1       RGLVFE---- --GGN----T LD-------- --------DL ATVVSNACIW LQDNNVPYNV LISDCGKRVF LFP------- ---QCYAEKQ ALGEVSQELL DTQVNPAVWE ISGHMVLKRR
ZmGGP-2       NGFVFE---- --GGA----S LE-------- --------DL SDVVSKVCIF LQENNRPFNV LISESGKRIF LLP------- ---QCYAEKQ LLGKASQEFL DMRINPAIWE LSGHLVLKRR
ZomGGP        RGLVFE---- --GGN----T LR-------- --------DI SDMVSAACIC LQETNIPFNV LISDSGRRIF IFP------- ---QCYAEKQ ALGEVSQEIL DTQVNPAVWE ISGHIVLKRK


              ....|....| ....|....| ....|....| ....|....| ....|....| ....|....| ....|....| ....|....| ....|....| ....|....| ....|....| ....|....|
                     970        980        990       1000       1010       1020       1030       1040       1050       1060       1070       1080
AcGGP         EDYEEASEGN AWRLLAEV-S LSEERFEEVK ALI------- ---------- ---------- --------FE A--------- ---------- ---------- -ISCA-DDR- SGS---TAEN
AcGGP-R-1     EDYEEASEGN AWRLLAEV-S LSEERFEEVK ALI------- ---------- ---------- --------FE A--------- ---------- ---------- -ISCA-DDG- SGS---TAEN
AcGGP-R-2     KDYDDASVEN AWRLLAEV-S LSEERFQEVK TLV------- ---------- ---------- --------LE A--------- ---------- ---------- -AELQ-EAG- PEE-------
AdGGP         EDYEEASEGN AWRLLAEV-S LSEERFEEVK ALI------- ---------- ---------- --------FE A--------- ---------- ---------- -ISCA-DDG- SGS---TAEN
AeGGP         EDYQEASEGN AWRLLAEV-S LSEERFEEVK ALI------- ---------- ---------- --------FE A--------- ---------- ---------- -ISCA-DDR- SGS---TAEN
ArGGP         EDYQEASEGN AWRLLAEV-S LSEERFEEVK ALI------- ---------- ---------- --------FE A--------- ---------- ---------- -ISCA-DDR- SGS---TAEN
AmhGGP-1      KDYDEASEEN AWRLLAEV-S LSEARFEEVK AMI------- ---------- ---------- --------FE A--------- ---------- ---------- -IACT-IVE- NGS-----DK
AmhGGP-2      EDFEKASEEY VWKLLSEV-S LSEDRFEEIK AAV------- ---------- ---------- --------LE A--------- ---------- ---------- -SGLQ-ESN- PDR-------
AncGGP-3      KDFDEASEEG ICKFLAQV-A LSEQEFQLVK ESI------- ---------- ---------- --------LK S--------- ---------- ---------- -LSGG-PAG- KKN-------
AncGGP-1      KDYEEASESY AWRLLAEV-S LSEERFQEVK AYI------- ---------- ---------- --------FE A--------- ---------- ---------- -TGLV-EAE- DKE-------
AncGGP-2      KDYEEASEDS AWRLLAEV-S LSAERFRQVK GCI------- ---------- ---------- --------FD A--------- ---------- ---------- -LGLA-VSE- EES-------
AqcGGP        KDYEEASEEN AWRLLAEV-S LSEERFEEVK TLM------- ---------- ---------- --------FE A--------- ---------- ---------- -IGCS-YDG- ID--------
AhGGP-1       EDYEGASEDN AWRLLAEA-S LSEERFKEVI ALA------- ---------- ---------- --------FE A--------- ---------- ---------- -IGSS-NHE- EDL-------
AhGGP-2       EDYEGASEEK AWRLLAEV-S LSEERFKEVN TMI------- ---------- ---------- --------FE A--------- ---------- ---------- -IGFS-SHE- EEEK------
AlGGP-1       EDYEGASEDN AWRLLAEA-S LSAERFKEVI ALA------- ---------- ---------- --------FE A--------- ---------- ---------- -IGCS-NHE- EDL-------
AlGGP-2       EDYEGASEEK AWRLLAEV-S LSEERFKEVN TMI------- ---------- ---------- --------FE A--------- ---------- ---------- -IGFS-SHE- EEE-------
AtGGP-1       EDYEGASEDN AWRLLAEA-S LSEERFKEVT ALA------- ---------- ---------- --------FE A--------- ---------- ---------- -IGCS-NQE- EDL-------
AtGGP-2       EDYEGASEEK AWRLLAEV-S LSEERFREVN TMI------- ---------- ---------- --------FD A--------- ---------- ---------- -IGFS-SHE- EEE-------
BosGGP-1      EDYEGASEEK AWSLLAEA-S LSEERFKEVN ALI------- ---------- ---------- --------FE A--------- ---------- ---------- -IGCS-DQE- ENP-------
BosGGP-2      EDYEEASEEK AWSLLAEV-S LSEERFKEVN TMI------- ---------- ---------- --------FE A--------- ---------- ---------- -IGLS-SHE- EAE-------
BdGGP-1       DDYEEASEAS AWRLLAEV-S LSESRFEEVK AYI------- ---------- ---------- --------FD A--------- ---------- ---------- -AGLV-QSL- AEE-------
BdGGP-2       ADFEEASEAS AWRLLAEV-S LSEERFEEVK ACI------- ---------- ---------- --------FQ G--------- ---------- ---------- -TGLT-ESD- EED-------
BdGGP-3       KDYDDASEAT LCRFLVEA-T LSGAEFQELK SCV------- ---------- ---------- --------LE F--------- ---------- ---------- -LASA-SPQ- RVD-------
BsGGP-1       DDYEEASEAS AWRLLAEV-S LSESRFEEVK AYI------- ---------- ---------- --------FD A--------- ---------- ---------- -AGLV-QSQ- AEE-------
BsGGP-2       ADFKEASEAS AWRLLAEV-S LSEERFEEVK ACI------- ---------- ---------- --------FQ A--------- ---------- ---------- -SGLT-ESD- EED-------
BsGGP-3       KDYAEASEAT LCRFLVEA-S LSAAEFQELK SCV------- ---------- ---------- --------LE F--------- ---------- ---------- -LAVG-ASPE RVD-------
BoGGP-1       EDYEGASEEN AWRLLAEA-S LSEERFKEVN ALI------- ---------- ---------- --------FE A--------- ---------- ---------- -IGCS-YQG- EEL-------
BoGGP-2       EDYEGATEEK ACRLLSDA-S LSEERFKEVN ALI------- ---------- ---------- --------FE A--------- ---------- ---------- -IGCS-KPR- ----------
BoGGP-3       EDYEGASEEK AWSLLAEV-S LSEERFKEVN TMI------- ---------- ---------- --------FE A--------- ---------- ---------- -IGCS-GEE- EEE-------
BrGGP-1       EDYEGASEEN AWRLLAEA-S LSEERFKEVN ALI------- ---------- ---------- --------FE A--------- ---------- ---------- -IGCS-YQG- EEL-------
BrGGP-2       EDYEGASEEN AWRLLAEA-S LSEERFKEVN ALI------- ---------- ---------- --------FD A--------- ---------- ---------- -IGCS-NQE- EEL-------
BrGGP-3       EDYEGATEEK ACRLLADA-S LSEERFKEVN ALI------- ---------- ---------- --------FE A--------- ---------- ---------- -IGC------ ----------
BrGGP-4       EDYGGEGVES TG-------- ---------- ---------- ---------- ---------- ---------- ---------- ---------- ---------- ---------- ----------
CgGGP-1       EDYEGASEEN AWRLLEEA-S LSEERFKEVN VLI------- ---------- ---------- --------FE A--------- ---------- ---------- -IGCS-DQE- EDH-------
CgGGP-2       EDYEEASEEK AWRLLAEV-S LSEERFKEVN TMI------- ---------- ---------- --------FE A--------- ---------- ---------- -IGVS-GHE- EAE-------
CrGGP-1       EDYEGASEEN AWRLLEEA-S LSEERFKEVN ALI------- ---------- ---------- --------FE A--------- ---------- ---------- -IGCS-DQE- EDH-------
CrGGP-2       EDYEEASEEK AWRLLAEV-S LSEERFKEVN TMI------- ---------- ---------- --------FE A--------- ---------- ---------- -IGVS-GHE- EAE-------
ChrGGP        EDFALADEAW AARLLSGV-S LSEERFMEVA NMC------- ---------- ---------- --------FG S--------- ---------- ---------- -SA------- ----------
CcGGP-1       KDYEEASEEN AWRLLAEV-S LSEERYQEVN ALI------- ---------- ---------- --------FE A--------- ---------- ---------- -IARG-DDA- NGG---VAES
CcGGP-2       KDFEEASEEY AWR------- ----RFQEVK AYV------- ---------- ---------- --------LE A--------- ---------- ---------- -VGLQ-KPI- LEE-------
CsGGP-1       KDYEEASEEN AWRLLAEV-S LSEERYQEVN ALI------- ---------- ---------- --------FE A--------- ---------- ---------- -IARG-DDA- NGG---VAES
CsGGP-2       KDFEEASEEY AWRVLSEV-S LSEERFQEVK AYV------- ---------- ---------- --------LE A--------- ---------- ---------- -VGLQ-KPI- LEE-------
CosGGP        QDYEHVSQDA VWRLLSYA-S YSEADVLEMA RKV------- ---------- ---------- --------LA V--------- ---------- ---------- ---------- ----------
CusGGP        KDYEEASEEN AWRLLAVV-S LSEERFQEVC ALI------- ---------- ---------- --------FE V--------- ---------- ---------- -IDTV-DAE- NTE---GSSS
DcGGP-1       EDYEEASEEK AWRLLAEV-S LSEERFKEVK DII------- ---------- ---------- --------FD A--------- ---------- ---------- -VSSA-ANE- NEL---IS--
DcGGP-2       EDYDEASEEN AWRLLAEV-S LSEERFQEVK DII------- ---------- ---------- --------FE A--------- ---------- ---------- -ITCA-VDE- CLV---TTRS
DsGGP         TDYENIDQDW AWRLLEQV-S LSEERFLALV NTI------- ---------- ---------- --------FG K--------- ---------- ---------- ---------- ----------
EgGGP-1       KDYEEASEEN AWRLLAEV-S LSEERFREVS DLI------- ---------- ---------- --------FE A--------- ---------- ---------- -ISCS-IDD- ------VAPS
EgGGP-2       KDFEEASEEN AWRLLAEV-S LSEERFQEVN ALI------- ---------- ---------- --------FE A--------- ---------- ---------- -IASV-ENG- GQD-------
EgGGP-3       KNFENATEDY AWRLLAEV-S LSEESFREVK A--------- ---------- ---------- ---------- ---------- ---------- ---------- ---------- ----------
EgGGP-4       EDFENASEDY AWRLLAEV-S LSEESFREVK AQI------- ---------- ---------- --------SE A--------- ---------- ---------- -AGLC-EID- TEA-------
EgGGP-5       EDFENALEDY AWRLLAEV-S LSEESFREVK A--------- ---------- ---------- ---------- ---------- ---------- ---------- ---------- ----------
EsGGP-1       EDYEGASEEN AWRLLAEA-S LSEERFKEVN DLI------- ---------- ---------- --------FE A--------- ---------- ---------- -IGCS-NQE- EEL-------
EsGGP-2       EDYQGASEEK AWSLLAEV-S LSEERFKEVN TMI------- ---------- ---------- --------FE A--------- ---------- ---------- -MGFS-GEE- EEL-------
FvGGP-1       KDYDEASDEN AWKLLAEV-S LSEERFQEVN ALI------- ---------- ---------- --------FG A--------- ---------- ---------- -IASGVDDG- NAN-------
FvGGP-2       KDYDEASDEN AWKLLAEV-S LSEERFQE-N ALFCSNEQIC GFVPVNLVLQ FAIWVYVSPI PAEPHSGDFQ ACKSSRRDLE ASRLHPSLLL VKNFEGDTVL HVAAKTGEG- DDS---MIRY
FvGGP-3       KDYDEASDEN AWKLLAEV-S LSEERFKEVN ALI------- ---------- ---------- --------IG A--------- ---------- ---------- -IAYG-G--- ----------
```

```
GmGGP-1     KDYDEASEGN AWRLLAEV-S LSQERFQEVN DLV------- ---------- ---------- --------FE A--------- ---------- ---------- -IGCG-ELE- DVN-------
GmGGP-2     KDYDEASEGN AWRLLAEV-S LSQERFQEVN DLI------- ---------- ---------- --------FE A--------- ---------- ---------- -IGCG-ELE- DVN-------
GmGGP-3     KDYEEASEAN AWRLLAEV-S LSEERLLEVT ALV------- ---------- ---------- --------FQ A--------- ---------- ---------- -ITSS-VEM- ----------
GmGGP-4     KDYEEASEAN ACRLLAEV-S LSEERFQESH CSC------- ---------- ---------- --------FS SH-------- ---------- ---------- CIG------- ----------
GnmGGP      EDYDRASEDY AWRLLAEV-S LSEGRFEEVK GYI------- ---------- ---------- --------LE A--------- ---------- ---------- -AAAP-QTI- EIN---PKQ-
GrGGP-1     EDYDEASEGN AWRLLAEV-S LSDERFCEVN ALI------- ---------- ---------- --------FE A--------- ---------- ---------- -IAGS-KDV- IKN---GTKT
GrGGP-2     KDYDEASDEN AWRLLAEV-S LSDERFREVN ALI------- ---------- ---------- --------FE V--------- ---------- ---------- -IASG-KDG- IEH---AAKS
GrGGP-3     KDYDEASDEN AWRLLAEV-S LSDERFDEVN ALI------- ---------- ---------- --------FE A--------- ---------- ---------- -IGGG-KDA- IEN---V---
KfGGP-1     KDFEEASEQN AWRLLAEV-S LSGERFQEVN TLI------- ---------- ---------- --------FD A--------- ---------- ---------- -LSCR-VDE- STN---PAQ-
KfGGP-2     KDFEEASEQN AWRLLAEV-S LSEERFHEVN TLI------- ---------- ---------- --------FE A--------- ---------- ---------- -LSCS-VDE- NIN---SAQ-
KlGGP-1     KDFEEASEQN AWRLLAEV-S LSGERFQEVN TLI------- ---------- ---------- --------FD A--------- ---------- ---------- -LSCR-VDE- STN---PAQ-
KlGGP-2     KDFEEASEQN AWRLLAEV-S LSEERFHEVN TLI------- ---------- ---------- --------FE A--------- ---------- ---------- -LSCS-VDE- NIN---SAQ-
KlGGP-3     KDFEEASEQN AWRLLAEV-S LSEERFHEVN TLI------- ---------- ---------- --------FE A--------- ---------- ---------- -LSCS-VDE- NIN---SAQ-
LuGGP       KDYEEASEEN AWRLLAEV-S LSEERLEEVK ALI------- ---------- ---------- --------FE A--------- ---------- ---------- -ISYS-LSG- SDK---EETQ
MdGGP       KDYDEASDEN AWRLLAEV-S LSEERFQEVN ALI------- ---------- ---------- --------FE R--------- ---------- ---------- -IASG-NNG- NEN-------
MeGGP-1     EDYEGASEEN AWRLLAEV-S LSEARFEEVN ALI------- ---------- ---------- --------FE A--------- ---------- ---------- -ISCS-HSG- SEN---DAQA
MeGGP-2     KDYEEASEEN AWRLLAEV-S LSEARFEEVN ALI------- ---------- ---------- --------FE A--------- ---------- ---------- -ISCS--NG- TEN---DGQD
MeGGP-3     QDFENASEAS AWRLLSDV-S LSEERFRQVK AYV------- ---------- ---------- --------LE A--------- ---------- ---------- -AGFQ-ETS- NTD-------
MpGGP       EDFDIATEDY AKKLLAEV-T LSEDRFTEVK KMC------- ---------- ---------- --------ID A--------- ---------- ---------- -AEDE-KLD- NCE-------
MtGGP-1     KDFDEASEAN AWRLLAEV-S LSKERFQEVN AII------- ---------- ---------- --------FE A--------- ---------- ---------- -IALT-EEL- DDN-------
MtGGP-2     KDFDEASEAN ARELLAEV-S LSKERFEEVN ALI------- ---------- ---------- --------FQ A--------- ---------- ---------- -ITSE----- ----------
MpGGP       EDFETCDEAS ATRLLSCA-S LSEEDYDAAC ADV------- ---------- ---------- --------LA L--------- ---------- ---------- -LDAA-ETG- GAD---DASA
MsGGP       DDYDACTQDA AFKMLACA-S LGEEEFYETC SHI------- ---------- ---------- --------LA E--------- ---------- ---------- -TESA-KGL- -----SDAMH
MgGGP-1     EDYEGASEEN AWRLLAEV-S LSEERLNEVK ELI------- ---------- ---------- --------FE A--------- ---------- ---------- -ASCF-NNV- EENKVVAEVA
MgGGP-2     EDYEEASEEN AWRLLAEV-S LSEERLEEVK ELI------- ---------- ---------- --------FE A--------- ---------- ---------- -ICCN-VEE- GVL---PPI-
MaGGP-1     KDYEEASEEY AWRLLAEV-S LSEARFEEVK AYI------- ---------- ---------- --------FE A--------- ---------- ---------- -TGL--ESE- EEK-------
MaGGP-2     KDYEEASEDY AWRLLAEV-S LSEERFHEVK TYI------- ---------- ---------- --------FE A--------- ---------- ---------- -IGLV-EPE- EEM-------
MaGGP-3     KDYDEASEHY AWRLLAEV-S LSEARFEEVK VYI------- ---------- ---------- --------FE A--------- ---------- ---------- -TGLV-ESE- KEK-------
MaGGP-4     KDYQETSEDT LWRFLDEVAS VSAEQFKEIK ECI------- ---------- ---------- --------LS T--------- ---------- ---------- -LSDK-IVE- ----------
OtGGP       NDYEEASETS AWKLLAEV-S LSEERFEEVK AYV------- ---------- ---------- --------FG A--------- ---------- ---------- -AGLV-QSD- -EE-------
OsGGP-1     SDYEEASEAS AWRLLAEV-S LSEERFEEVK AYI------- ---------- ---------- --------FD A--------- ---------- ---------- -AGLV-QSD- --E-------
OsGGP-2     KDYDEASEAT ICRFLVEA-S LSESEFQELK SCI------- ---------- ---------- --------LE F--------- ---------- ---------- -LSSA-APE- E---------
OlGGP       SDYDQCTETS AEELLACA-S LTEEQF---- ---------- ---------- ---------- ---------- ---------- ---------- ---------- ---------- ----------
PhGGP-1     NDYEEASETS AWKLLAEV-S LSEERFEEVK AYI------- ---------- ---------- --------FT A--------- ---------- ---------- -AGLV-QAD- --E-------
PhGGP-2     KDYDEASEPN ICRFLVEA-A LSETEFQELK RCV------- ---------- ---------- --------LD F--------- ---------- ---------- -LTVS-DAC- NLD-------
PvGGP-1     NDYEEASETS AWKLLAEV-S LSEERFEEVK AYI------- ---------- ---------- --------FT A--------- ---------- ---------- -AGLV-QAD- --E-------
PvGGP-2     NDYEEASETS AWKLLAEV-S LSEERFEEVK AYI------- ---------- ---------- --------FT A--------- ---------- ---------- -AGLV-QAD- --E-------
PvGGP-3     KDYDEASEPN ICRFLVEA-A LSVTEYQELK RCV------- ---------- ---------- --------LD F--------- ---------- ---------- -LAVS-YAC- NSD-------
PhvGGP-1    KDYDEASEGN AWRLLAEV-S LSEERFQEVN DLI------- ---------- ---------- --------FE A--------- ---------- ---------- -IDCG-DN-- ----------
PhvGGP-2    KDYDEASEAN AWRLLAEV-S LSEERYEEVT ALV------- ---------- ---------- --------FQ A--------- ---------- ---------- -IASV----- ----------
PpGGP-1     KDYDLATEGY AWRLMAEV-S LSQERFEEVK AKC------- ---------- ---------- --------LK A--------- ---------- ---------- -AFQV-ISV- TSD---YLAG
PpGGP-2     KDYDMATEDY AWKLLAEV-S LPQERFDEVK AEC------- ---------- ---------- --------LK A--------- ---------- ---------- -AFDG-CHQ- GDV---VNVG
PpGGP-3     TDYVLATEEY AWKLLAEV-S LSRGRFEEVK VKC------- ---------- ---------- --------LK A--------- ---------- ---------- -AFHE-VSD- GDD---HEMA
PaGGP-1     QDFDRASEDY ACKLLAEV-S LSEQRFEEVK DYI------- ---------- ---------- --------LE A--------- ---------- ---------- -AATG-EMQ- TEE-------
PaGGP-2     QDFDRASEDY AWRLLAEV-S LSEERFKEVK DYI------- ---------- ---------- --------LE A--------- ---------- ---------- -ATSG-EVQ- TEE-------
PaGGP-3     QDFDRASEDY AWKLLAEV-S LSEERFEEVK AYI------- ---------- ---------- --------LE A--------- ---------- ---------- -AATG-QVQ- VVE-------
PsGGP-1     QDFDRASEDY ACKLLAEV-S LSEERFEEVK DYI------- ---------- ---------- --------LE A--------- ---------- ---------- -AATG-EMQ- TEE-------
PsGGP-2     QDFDRASEDY AWRLLAEV-S LSEERFEEVK AYI------- ---------- ---------- --------FE A--------- ---------- ---------- -VASG-EVQ- TEE-------
PsGGP-3     QDFDRASEDY AWKLLAEV-S LSEERFEEVK AYI------- ---------- ---------- --------LE A--------- ---------- ---------- -AATG-QVQ- VVE-------
PtGGP-1     QDFDRASEDY ACKLLAEV-S LSEERFEEVK DYI------- ---------- ---------- --------LE A--------- ---------- ---------- -VATG-EVQ- TKE-------
PtGGP-2     QDFDRASEDY AWRLLAEV-S LSEERFEEVK AYI------- ---------- ---------- --------LE A--------- ---------- ---------- -AATE-EVQ- VEE-------
PtGGP-3     QDFDRASEDY AWKLLAEV-S LSEERFKEVK DNI------- ---------- ---------- --------LE A--------- ---------- ---------- -ATSG-EVQ- TEE-------
PtGGP-4     QDFDRASEDY AWKLLAEV-S LSEERFEEVE AYI------- ---------- ---------- --------LE A--------- ---------- ---------- -VASG-EVQ- TEE-------
PtGGP-5     QDFDRASEDY AWKLLAEA-S LSEERFEEVK AYI------- ---------- ---------- --------LE A--------- ---------- ---------- -AVTE-EVQ- VEE-------
PotGGP-1    KDYEEASEEN AWRLLAEV-S LSEERFQEVT ALI------- ---------- ---------- --------FE A--------- ---------- ---------- -ISYR-SCV- IGT---DSEN
PotGGP-2    KDYEEASEEN AWRLLAEV-S LSEERFQEVN ALI------- ---------- ---------- --------FK A--------- ---------- ---------- -ISNS-GNC- SGT---DSEN
PotGGP-3    EDFDDASETY AWRLLAEV-S LSNKRFHQVK AYL------- ---------- ---------- --------LE A--------- ---------- ---------- -AGFQ-TEI- EEN-------
PrpGGP      KDYEEASDEN AWKLLAEV-S LSEERFQEVN SLI------- ---------- ---------- --------FE A--------- ---------- ---------- -IASD-DNG- NAN-------
RcGGP-1     EDYEEASEEN AWRLLSEV-S LSEARFQEVN ALI------- ---------- ---------- --------FE A--------- ---------- ---------- -ISYA-GSG- SDH---EAQN
RcGGP-2     KDYEEASEEN AWRLLAEV-S LSEARFQEVN ALI------- ---------- ---------- --------FE A--------- ---------- ---------- -ISYA-GSS- SDN---EAQN
RcGGP-3     KDFEDASEIS AWRLLAEV-S LSEERFKEVK ACI------- ---------- ---------- --------LE A--------- ---------- ---------- -AGFQ-EEV- LAE-------
SpGGP-1     KDYEDASEEN AWRLLAEV-S LSEERFQEVN ALI------- ---------- ---------- --------FE A--------- ---------- ---------- -IYYS-GNG- SGA---DSEN
SpGGP-2     KDYEEASEEN AWRLLAEV-S LSEERFQEVS ALI------- ---------- ---------- --------FE A--------- ---------- ---------- -ISYS-SHL- DGT---DSEK
SpGGP-3     EDFDDASETC AWRILAEV-S LSNKRFNEVK AHI------- ---------- ---------- --------LE A--------- ---------- ---------- -AGFQ-TET- EEK-------
SmGGP-1     EDFERATEEY AWRLLAEV-S VSEKVFDEII KLV------- ---------- ---------- --------FS S--------- ---------- ---------- -AEDKFDAK- ----------
SmGGP-2     QDFENATQEY AWKLLAEV-S LNDKAFNKII NLV------- ---------- ---------- --------TN D--------- ---------- ---------- -KYYS----- ----------
SmGGP-3     EDYEHATEEF VTALLAEV-S LSDPDFQDVK NII------- ---------- ---------- --------CK ---------- ---------- ---------- ---------- ----------
SiGGP-1     NDYEEASETS AWKLLAEV-S LSEERFEEVK AYI------- ---------- ---------- --------FT A--------- ---------- ---------- -AGLV-QAD- --E-------
SiGGP-2     KDYDEASELY ICRFLVEA-A VSETEFQQLK QCV------- ---------- ---------- --------LD F--------- ---------- ---------- -LTAS-AAC- NSD-------
SvGGP-1     NDYEEASETS AWKLLAEV-S LSEERFEEVK AYI------- ---------- ---------- --------FT A--------- ---------- ---------- -AGLV-QAD- --E-------
SvGGP-2     KDYDEASELY ICRFLVEA-A VSETEFQQLK QCV------- ---------- ---------- --------LD F--------- ---------- ---------- -LTAS-AAC- NSD-------
SlGGP-1     EDYEGATEAN AWRLLAEV-S LSEARFQEVT ALI------- ---------- ---------- --------FE A--------- ---------- ---------- -ISLS-VEE- NED---GTDG
SlGGP-2     KDYNDASEEY AWKLLSEV-S ISEERFEEVK GYI------- ---------- ---------- --------SE A--------- ---------- ---------- -ADLQ-ADE- DEN-------
StGGP-1     EDYEGATEAN AWRLLAEV-S LSEARFQEVT ALI------- ---------- ---------- --------FE A--------- ---------- ---------- -ISLS-VEE- NEN---ATDG
StGGP-2     KDYNDASEEY AWKLLSEV-S LSEERFEEVK GYI------- ---------- ---------- --------SE A--------- ---------- ---------- -DGLQ-AEE- DKN-------
SbGGP-1     SDYEEASETS AWKLLAEV-S LSEERFEEVK AYI------- ---------- ---------- --------FS A--------- ---------- ---------- -AGLV-LSD- EEE-------
SbGGP-2     KDYDEASEAN ICRFLVEA-A LSETEFQELN RCL------- ---------- ---------- --------LD F--------- ---------- ---------- -VTSW----- ----------
SfGGP-1     LDYESATEES AWRLLAEV-S LTQERFDEVK KHC------- ---------- ---------- --------LK S--------- ---------- ---------- -VFNA-PNK- VKV-------
SfGGP-2     CDYDTATEES AWRLLAEV-S LTQERFNEVK KHC------- ---------- ---------- --------LT A--------- ---------- ---------- -VLKS-SPD- GDEC------
SppGGP-1    QDYEEASEAY AWRLLAEV-S LSEERFEEVK ASL------- ---------- ---------- --------FE A--------- ---------- ---------- -AGLV-VAA- GEE-------
SppGGP-2    KDYDDASEDY AWRLLAEV-S LSEERFKEVQ AYI------- ---------- ---------- --------FE A--------- ---------- ---------- -TGLV-EEP- EEE-------
TcGGP-1     KDYDEASDEN AWRLLAEV-S LSEERFREVN ALI------- ---------- ---------- --------FE A--------- ---------- ---------- -IAIG-ETI- -------AET
TcGGP-2     EDYEDTSEAY AWRLLAEV-S LSEERFQEVK AYI------- ---------- ---------- --------LE A--------- ---------- ---------- -AALQ-ATN- MKD-------
TpGGP       KDFDEASEAN AWRLLAEV-S LSEERFQEVN ALI------- ---------- ---------- --------FE A--------- ---------- ---------- -IALT-EEL- DDN-------
VvGGP-1     EDYEGASEQN AWRLLAEV-S LSEERFQEVN ALI------- ---------- ---------- --------FE A--------- ---------- ---------- -IAYA----- ----------
VvGGP-2     EDYENASEEY AWRLLAEV-S LSEERFQEVK GYV------- ---------- ---------- --------LE A--------- ---------- ---------- -SGLQ-EAD- MEE-------
VcGGP       QDYTEATEDL AMQLLAQA-S LSEERFMSLA RLC------- ---------- ---------- --------FG G--------- ---------- ---------- -AAGA-DIC- ----------
ZmGGP-E-1   MDYEEASETS AWKLLAEV-S LSEERFEEVK AYI------- ---------- ---------- --------FS A--------- ---------- ---------- -AGLV-QYD- EEE-------
ZmGGP-E-2   KDYEEASEAN ICRFLVEA-A LSETEFQELN LCV------- ---------- ---------- --------LD F--------- ---------- ---------- -LTSS----- ----------
ZmGGP-1     MDYEEASETS AWKLLAEV-S LSEERFEEVK AYI------- ---------- ---------- --------FS A--------- ---------- ---------- -AGLV-QYD- EEE-------
ZmGGP-2     KDYEEASEAN ICRFLVEA-A LSETEFQELN RCV------- ---------- ---------- --------LD F--------- ---------- ---------- -LTSS----- ----------
ZomGGP      KDFEETSEVH ASRLLSEV-S LSEERFDEVK SYI------- ---------- ---------- --------FE A--------- ---------- ---------- -LGLV-AAV- DVD-------


            ....|....| ....|....| ....|....| ....|....| ....|....| ....|....| ....|....| ....|....| ....|....| ....|....| ....|....| ....|....|
                  1090       1100       1110       1120       1130       1140       1150       1160       1170       1180       1190       1200
AcGGP       LLE------- ---------- ---------- -EPDDN-PQ- SR-K------ -------VAN ---------- ----DALN-- -----KGSHR -G-------- ---------M VPGKQECLVQ
AcGGP-R-1   LLE------- ---------- ---------- -EPDDN-PQ- SR-E------ -------EAN ---------- ----DALN-- -----KGSHC -G-------- ---------M VPGKQECLVQ
AcGGP-R-2   ---------- ---------- ---------- -------KS- MN-------- -------EEE ---------- -----SLQ-- -----KPRAP YV-------- ---------A SQLSQDCLVL
AdGGP       LLE------- ---------- ---------- -EPDDN-PQ- SR-E------ -------EAN ---------- ----DALN-- -----KGSHC -G-------- ---------M VPGKQECLVQ
AeGGP       LVE------- ---------- ---------- -EPDND-PQ- SR-E------ -------VAN ---------- ----DALN-- -----KASHR -G-------- ---------M VPGKQECLVQ
ArGGP       LLE------- ---------- ---------- -EPDEN-PQ- SH-E------ -------VTN ---------- ----DALN-- -----KGSHR -G-------- ---------M VPGKQECLVQ
AmhGGP-1    FLE------- ---------- ---------- -EADLISSP- AL-R------ -------DMD ---------- -----SIN-- ------SNAS -A-------- ---------M VTGNHECVVL
AmhGGP-2    ---------- ---------- ---------- -------TS- IP-GTKGK-- -------EKS ---------- ---------- -----SLDAC DS-------- ---------T RLLLRDGIVL
AncGGP-3    ---------- ---------- ---------- ---------- ---------- -------TGE ---------- ---------- ---------- ---------- ---------- ----------
AncGGP-1    ---------- ---------- ---------- -------VG- KK-E------ -------SEE ---------- -----ASC-- -----QS-TP VG-------- ---------P PHFPEGCLVL
AncGGP-2    ---------- ---------- ---------- --------E- AN-E------ -------SLD ---------- -----STR-- -----MCDSS VK-------- ---------- ----------
AqcGGP      ---------- ---------- ---------- -TDNNE-AE- FE-K------ -------EAD ---------- -----ASS-- -----DGAHP -T-------- ---------I VPGTQECMVN
AhGGP-1     ---------- ---------- ---------- -EGTIV-HQ- QN-P------ -------SGN ---------- ------VNQK ----SNRTHG GP-------- --------IT NGTAAECLVL
AhGGP-2     ---------- ---------- ---------- ---------- EL-E------ -------EQS ---------- -----SMN-- -----GSSFI GV-------- ---------H CPSVKEEAVS
AlGGP-1     ---------- ---------- ---------- -EGTIV-HQ- QN-P------ -------SGN ---------- ------VNQK ----SNQTHG GP-------- --------IT NGTAAECLVL
AlGGP-2     ---------- ---------- ---------- ---------- VL-E------ -------EQS ---------- -----SMN-- -----GSSFI RV-------- ---------H CPSVKEEAVS
AtGGP-1     ---------- ---------- ---------- -EGTIV-HQ- QN-S------ -------SGN ---------- ------VNQK ----SNRTHG GP-------- --------IT NGTAAECLVL
AtGGP-2     ---------- ---------- ---------- -------EE- EL-E------ -------EQN ---------- -----SMN-- -----GGSFT IV-------- ---------H CPSVKEEAVS
BosGGP-1    ---------- ---------- ---------- -EGTIV-HQ- QS-P------ -------NGG ---------- ------VNQK ----SNRIHG GP-------- --------IT NGTASECLVL
BosGGP-2    ---------- ---------- ---------- -------EE- EL-E------ -------EQT ---------- -----SMN-- -----SG-FI RV-------- ---------H CPSVKEEAVS
BdGGP-1     ---------- ---------- ---------- --------E- TG-E------ -------ADD ---------- -----AI--- -----YTPVT VA-------- ---------P QAVTEGCLVL
```

```
BdGGP-2    ---------- ---------- ---------- --------E- TN-E------ -------ESP ---------- -----YAS-- -----SSSAP VA-------- ---------S SHIPEGCLVL
BdGGP-3    ---------- ---------- ---------- -------IK- ---------- ---------- ---------- ---------- ---------- ---------- ---------- -PF-------
BsGGP-1    ---------- ---------- ---------- --------E- TG-E------ -------ADD ---------- -----AI--- -----YTPVT VA-------- ---------P PAVAEGCLVL
BsGGP-2    ---------- ---------- ---------- --------E- TN-E------ -------ESP ---------- -----YAS-- -----SSSAP VA-------- ---------S LHIPEGCLVL
BsGGP-3    ---------- ---------- ---------- -------IK- ---------- ---------- ---------- ---------- ---------- ---------- ---------- -PF-------
BoGGP-1    ---------- ---------- ---------- -EGTVI-GQ- DN-P------ -------SGS ---------- ------VNQK ----SNRTHG GP-------- --------IT NGTASECLVL
BoGGP-2    ---------- ---------- ---------- -------GR- ---------- ---------- ---------- ---------- ---------- -A-------- ---------- ----------
BoGGP-3    ---------- ---------- ---------- -------LE- ---E------ -------ESS I--------- -----RVN-- ---------- ---------- ---------- ----------
BrGGP-1    ---------- ---------- ---------- -EGTVV-GQ- DN-P------ -------SGS ---------- ------VNQK ----SNRTHE GP-------- --------IT NGTASECLVL
BrGGP-2    ---------- ---------- ---------- -EGILV-Q-- ---P------ -------SGS ---------- ------VNQT ----GNRTLG GP-------- --------IA NGAASECLVL
BrGGP-3    ---------- ---------- ---------- ---------- ---------- ---------- ---------- ---------- ---------- ---------- ---------- ----------
BrGGP-4    ---------- ---------- ---------- ---------- ---------- ---------- ---------- ---------- ---------- ---------- ---------- ----------
CgGGP-1    ---------- ---------- ---------- -EGTVV-HQ- QS-P------ -------NGG ---------- -----VVNKN ----SNRIHG GV-------- --------IT NGTAAECIVL
CgGGP-2    ---------- ---------- ---------- -------DE- ---E------ -------QQT ---------- -----SMN-- -----SG-FI RV-------- ---------H CGSVKEDAVS
CrGGP-1    ---------- ---------- ---------- -EGTVV-HQ- QS-P------ -------NGG ---------- -----VVNQN ----SNRIHG GV-------- --------IT NGTAAECIVL
CrGGP-2    ---------- ---------- ---------- -------EE- ---E------ -------EQT ---------- -----SMN-- -----SG-FI RV-------- ---------H CPSVKEEAVF
ChrGGP     ---------- ---------- ---------- ---------- ---------- ---------- ---------- ---------- ---------- ---------- ---------- ----------
CcGGP-1    VIG------- ---------- ---------- -EADAK-PK- SG-G------ -------EVD ---------- -----AIN-- -----KNSCP -A-------- ---------M VSGTPECLVL
CcGGP-2    ---------- ---------- ---------- -------NG- II-Q------ -------EED ---------- -----SLD-- -----KLPAL EA-------- ---------- QRVVQNCTVQ
CsGGP-1    VIG------- ---------- ---------- -EADAK-PK- SG-G------ -------EVD ---------- -----AIN-- -----KNSCP -A-------- ---------M VSGTPECLVL
CsGGP-2    ---------- ---------- ---------- -------NG- II-Q------ -------EED ---------- -----SLD-- -----KLPAL EA-------- ---------- QR--------
CosGGP     ---------- ---------- ---------- ---------- ---------- ---------- ---------- ---------- ---------- ---------- ---------- ----------
CusGGP     MPE------- ---------- ---------- -EHD---DG- CL-K------ -------EVE ---------- -----ARN-- -----NTTRP -L-------- ---------P MTGTKECLV-
DcGGP-1    ---------- ---------- ---------- -QNDQE-HH- TN-Q------ -------ESG ---------- -----THN-- -----NGSSA -A-------- ---------L V--SHECLVL
DcGGP-2    SDE------- ---------- ---------- -DQDVE-GR- SH-E------ ---------- ---------- ---------- -----KTVNL -A-------- ---------I VPGKPECLVQ
DsGGP      ---------- ---------- ---------- ---------- ---------- -------E-- ---------- ---------- ---------- ---------- ---------- ----------
EgGGP-1    SLD------- ---------- ---------- -EPNAE-PL- AD-------- ---------- ---------- ---------- -F---AAPCP -N-------- ---------M VRGKKDCLVL
EgGGP-2    ---------- ---------- ---------- -NPDVE-PQ- AP-K------ -------DVD ---------- -----VAK-- -----QSSRP -A-------- ---------M VAGSQECLVR
EgGGP-3    ---------- ---------- ---------- ---------- ---------- ---------- ---------- ---------- ---------- ---------- ---------- ----------
EgGGP-4    ---------- ---------- ---------- -------HN- LH-Q------ -------NEE ---------- -----AVS-- -----TSSAA TA-------- ---------T SHLHQDCLVV
EgGGP-5    ---------- ---------- ---------- ---------- ---------- ---------- ---------- ---------- ---------- ---------- ---------- ----------
EsGGP-1    ---------- ---------- ---------- -EGTIV-HQ- QS-P------ -------SGS ---------- ------VNQK ----SNRTHG GP-------- --------IT NGTASECLVL
EsGGP-2    ---------- ---------- ---------- ---------- ---E------ -------EQS ---------- -----SMD-- -----GG-FI RA------Q- ---------C CPSVKEEAVS
FvGGP-1    LLE------- ---------- ---------- -DPDVN-PR- SH-D------ -------EVN ---------- -----AIN-- -----KSSHH -A-------- ---------M VSGTQECLVL
FvGGP-2    IMEHV---SL GSGQGQVTDI EK-------- VDISVF-RM- KN-D------ -------EEN ---------I PLHEALIN-- -----EAGLI -E-------- ---------I VTPIVDSIAI
FvGGP-3    ---------- ---------- ---------- -DPDVK-PR- PH-E------ -------RSH ---------- ---------- ---------- ---------- ---------- ----------
GmGGP-1    ---------- ---------- ---------- -------VQ- SVKK------ -------EVD ---------- -----AVS-- -----TSEHP -T-------- -------VVV VAGSQECVVL
GmGGP-2    ---------- ---------- ---------- -------VE- SV-K------ -------EVD ---------- -----AVS-- -----TSEHP -T-------- --------VV VAGSQECVIL
GmGGP-3    ---------- ---------- ---------- ---NVK-PQ- RL-E------ -------EVD ---------- -----AVS-- -----PNAQH -A-------- ---------M VAGSHECLVL
GmGGP-4    ---------- ---------- ---------- ---------- ---------- ---------- ---------- ---------- ---------- ---------- ---------- ----------
GnmGGP     ---------- ---------- ---------- -------EN- ES-TII---- -------EDE ---------V PLY-DALK-- -----DNHGS S--------- ---------T MVVAEECLVI
GrGGP-1    LLE------- ---------- ---------- -EP------- CL-E------ -------EDD ---------- -----AIT-- -----KSSHR GA-------- ---------M VAGTQQCLVL
GrGGP-2    LPK------- ---------- ---------- -EPNTK-AE- ST-E------ -------EES ---------- -----AIT-- -----KTSHH -A-------- ---------M VGGTQECVVL
GrGGP-3    --E------- ---------- ---------- -EPDTK-AQ- SI-E------ -------KEN ---------- -----GIT-- -----ETSHH -A-------- ---------M VAGTQQCLVL
KfGGP-1    -LD------- ---------- ---------- -EPDAE-SQ- PQ-D------ -------DK- ---------- ---------- -----EAAYQ -A-------- ---------M VTRKHECIVQ
KfGGP-2    -LD------- ---------- ---------- -APDAK-PR- PQ-D------ -------DK- ---------- ---------- -----EASCP -A-------- ---------M VASKHECIVQ
KlGGP-1    -LD------- ---------- ---------- -EPDAE-SQ- PQ-D------ -------DK- ---------- ---------- -----EAAYQ -A-------- ---------M VTRKHECIVQ
KlGGP-2    ---------- ---------- ---------- -------PR- PQ-D------ -------DK- ---------- ---------- -----EASCP -A-------- ---------M VASKHECIVQ
KlGGP-3    -LD------- ---------- ---------- -APDAK-PR- PQ-D------ -------DK- ---------- ---------- -----EASCP -A-------- ---------M VASKHECIVQ
LuGGP      V--------- ---------- ---------- -AGAKK-PE- SS-E------ -------GVG ---------- -----GIN-- -----KGPNC -A-------- ---------V VSGTPECLVL
MdGGP      LPE------- ---------- ---------- -DPEVK-PR- SH-E------ -------EVD ---------- ----ATIN-- -----KSSRA -A-------- ---------M VGETQECIVL
MeGGP-1    VQ-------- ---------- ---------- -DTNV--NN- SL-E------ -------EVD ---------- -----AIN-- -----KGSHR -A-------- ---------S VTGTQECLVQ
MeGGP-2    LLE------- ---------- ---------- -DPND--NK- SL-E------ -------EVD ---------- ----AAIN-- -----KGSHC -A-------- ---------M VSGTHECLVQ
MeGGP-3    ---------- ---------- ---------- ---------- ---Q------ -------EDD ---------- -----STY-- -----DQLSP RA-------- ---------I SHLPKDCVVI
MpGGP      ---------- ---------- ---------- -------VM- RS-------- -------NDD ---------- PYRGASVK-- -----AKNYK FAA------- --------KF QNLYDECAV-
MtGGP-1    ---------- ---------- ---------- -------VQ- CL-P------ -------KDD ---------- -----SVD-- -----SSTYP -T-------- ---------V VAGSQECVVL
MtGGP-2    ---------- ---------- ---------- -KIDVT-PQ- CL-E------ -------EVD ---------- -----AVS-- -----SSTKP -A-------- ---------M VPGSHECLVL
MpGGP      TSSPRGGLGL GLGGGRVSPL DDGWGGVGRG GSIPPS-SEY AN-DVAMTVP GRPRTMTTTT TTTTTSPGGS GIGMDAAKRR ----SAAESP THPP------ --------LV GSSDDEAVVP
MsGGP      AAV------- ---------- ---------- -------PEH TN-AVV---- --PDSNATTE ---------- AIEIAARKHR DTADADPGSP TSPP------ -------LLS DGDSADGVVP
MgGGP-1    TTA------- ---------- ---------- -SSEDL-GE- AC-E------ -------DGD ---------- -----ALK-- -----GSGEP -A-------- ---------M VHV-------
MgGGP-2    TVE------- ---------- ---------- -ESDDA-AE- SH-D------ -------DAD ---------- -----ALK-- ---------- ---------- --------ML ASCYGACVV-
MaGGP-1    ---------- ---------- ---------- -------IE- PE-E------ -------NKG ---------- -----TPHQ- -----SSTTA VA-------- ---------A NHFPEGCLIL
MaGGP-2    ---------- ---------- ---------- -------IH- EE-E------ -------EKG ---------- -----TPS-- -----NSSDP MH-------- ---------A PQFPEGCLIL
MaGGP-3    ---------- ---------- ---------- -------ME- PG-D------ -------DRG ---------- -----APF-- -----QSPSP VGT------- --------AS PHLKVA----
MaGGP-4    ---------- ---------- ---------- ---------- RN-------- -----MGRED ---------- ---------- ---------- ---------- ---------- ---YHKC---
OtGGP      ---------- ---------- ---------- -------EE- VS-E------ -------GED ---------- -----TT--- -----YAPVP VT-------- ---------P LPVTEGCLVL
OsGGP-1    ---------- ---------- ---------- -------EE- VS-E------ -------DED ---------- -----AT--- -----YTPVS IA-------- ---------P PAVAEGCLVL
OsGGP-2    ---------- ---------- ---------- ---------- ---------- ---------- ---------- ---------- ---------- ---------- ---------- ----------
OlGGP      ---------- ---------- ---------- ---------- ---------- ---------- ---------- ---------- ---------- ---------- ---------- ----------
PhGGP-1    ---------- ---------- ---------- -------EE- AS-E------ -------GEE ---------- -----AT--- -----YAPVP VA-------- ---------P LPVAEGCLVL
PhGGP-2    ---------- ---------- ---------- -------ID- ---------- ---------- ---------- ---------- ---------- ---------- ---------- -S--------
PvGGP-1    ---------- ---------- ---------- -------EE- VS-E------ -------GEE ---------- -----AT--- -----YTPVP VA-------- ---------P LPVAEGCLVL
PvGGP-2    ---------- ---------- ---------- -------EE- VC-E------ -------GEE ---------- -----AT--- -----YAPVP VA-------- ---------P LPVAEGCLVL
PvGGP-3    ---------- ---------- ---------- -------ID- ---------- ---------- ---------- ---------- ---------- ---------- ---------- -S--------
PhvGGP-1   ---------- ---------- ---------- -------VQ- RD-K------ -------EED ---------- -----AVS-- -----SSAHS -T-------- -------VVV VAGSQECVVL
PhvGGP-2   ---------- ---------- ---------- -DMNVK-PQ- CA-E------ -------EVD ---------- -----AVS-- -----PSTQP -S-------- ---------M VVGSQKCLVL
PpGGP-1    TSKRG----- ---------- ---------- -SFDFMVNE- DI-P------ -------AKD ---------- -----YLK-- -----TNSLD AVVPNHGDDL SVEA------ ----------
PpGGP-2    ACN------- ---------- ---------- -GIENG-NH- KA-S------ -------VKD ---------- -----SFK-- -----TSSMD SIVPGEG--V GVEG------ ----------
PpGGP-3    ATNAE----- ---------- ---------- -SINFM-AK- FA-P------ -------SKV ---------- -----NLQ-- -----SNMLD VVL-NHGEEL SVEG------ ----------
PaGGP-1    ---------- ---------- ---------- -------EE- RA-DVV---- -------EDE ---------V PVY-DSLK-- -----NNHNA ---------- ---------P MHMTEGRPIL
PaGGP-2    ---------- ---------- ---------- -------EE- QT-DVV---- -------EEK ---------V AVY-ESLK-- -----NKHNA ---------- ---------P MHMNEGCIVI
PaGGP-3    ---------- ---------- ---------- -------EE- QA-DIVE--- -------EDE ---------V PLY-DSSK-- -----NNHNA ---------- ---------P MPL---CI--
PsGGP-1    ---------- ---------- ---------- -------EE- RA-VVV---- -------EDE ---------V PVY-DSLK-- -----NNHNA ---------- ---------P MHITEGRPIL
PsGGP-2    ---------- ---------- ---------- -------EE- QA-DVV---- -------EDE ---------D PLH--SLK-- -----NNHDA ---------- ---------P MHLNEGCLVL
PsGGP-3    ---------- ---------- ---------- -------EE- QA-DIVE--- -------EDE ---------I PLY-DSSK-- -----NNHNA ---------- ---------P MPL---CI--
PtGGP-1    ---------- ---------- ---------- -------EE- RA-DIV---- -------EDE ---------V PVL-DSLK-- -----INHNT ---------- ---------P MHMNEGHPIF
PtGGP-2    ---------- ---------- ---------- -------EE- HA-DIVV--- -------EDG ---------V PLY-DSSK-- -----NT--- ---------- ---------I MPL---CT--
PtGGP-3    ---------- ---------- ---------- -------EEK QA-DVV---- -------EDE ---------V DVY-ESLK-- -----NNHNA ---------- ---------P MHMNEGFLVL
PtGGP-4    ---------- ---------- ---------- -------EE- EA-AVV---- -------EDE ---------D PLH-HSLK-- -----NNHDA ---------- ---------P VHLNEGCLVL
PtGGP-5    ---------- ---------- ---------- -------EE- HA-DIVV--- -------EDG ---------V PLY-DSSK-- -----NT--- ---------- ---------I MPL---CT--
PotGGP-1   LLE------- ---------- ---------- -DVNVE-HQ- LV-E------ -------EVN ---------- -----AIN-- -----ESSHQ -A-------- ---------M VTGNQDCLVL
PotGGP-2   VLE------- ---------- ---------- -DANVE-HT- SL-E------ -------EVN ---------- -----AIN-- -----ENSHS -A-------- ---------M VTGSPGCLVL
PotGGP-3   ---------- ---------- ---------- -------NR- DL-------- -------ERE ---------- -----PIY-- -----EQPSP EA-------- ---------V AHLPQDCMVF
PrpGGP     LLE------- ---------- ---------- -DPEVK-PH- SR-E------ -------EVD ---------- -----TIN-- -----TSSHA -A-------- ---------M VTGTQQCLVL
RcGGP-1    VLL------- ---------- ---------- -EDKN--VK- SA-E------ -------EVD ---------- -----AIN-- -----QSSHH -T-------- ---------M VTGNQECLIQ
RcGGP-2    MLE------- ---------- ---------- -DEDV---N- SV-G------ -------EVG ---------- -----AIN-- -----QSSHC -T-------- ---------M VTGNQECLVQ
RcGGP-3    ---------- ---------- ---------- -------NN- RN-H------ -------AEE ---------- -----SICS- -----DQQSP KA-------- ---------I SHLLQDCAVF
SpGGP-1    VLE------- ---------- ---------- -DANAK-HK- ST-E------ -------EVN ---------- -----AIN-- -----ETSHS -A-------- ---------M VTGSQGCLVL
SpGGP-2    LLE------- ---------- ---------- -DVNVK-HQ- PV-E------ -------EVN ---------- -----AIT-- -----ERSHP -A-------- ---------M VTG-----IL
SpGGP-3    ---------- ---------- ---------- -------NR- DH-------- -------EGE ---------- -----SIH-- -----DQPSP KA-------- ---------V AHLPQDCMVF
SmGGP-1    ---------- ---------- ---------- ----LY-HK- ---SLI---- -------STD ---------- ---------- ---------- ---------- --------IT YYTVGENVIM
SmGGP-2    ---------- ---------- ---------- ---------- KM-------- ---------- ---------- ---------- ---------- ---------- ---------- ----------
SmGGP-3    ---------- ---------- ---------- ---------- ---------- ---------- ---------- ---------- ---------- ---------- ---------- ----------
SiGGP-1    ---------- ---------- ---------- -------EK- VS-E------ -------GEE ---------- -----AT--- -----YKPVP VA-------- ---------P LPIAEGCLVL
SiGGP-2    ---------- ---------- ---------- -------ID- ---------- ---------- ---------- ---------- ---------- ---------- ---------- -S--------
SvGGP-1    ---------- ---------- ---------- -------EK- VS-E------ -------GEE ---------- -----AT--- -----YTPVP VA-------- ---------P LPIAEGCLVL
SvGGP-2    ---------- ---------- ---------- -------ID- ---------- ---------- ---------- ---------- ---------- ---------- ---------- -Y--------
SlGGP-1    SPE------- ---------- ---------- -DLDVTPPQ- PM-E------ -------EID ---------- -----GLN-- -------THS -T-------- ---------M VPA-------
SlGGP-2    ---------- ---------- ---------- -------IN- PE-K------ ---------- ---------- -----EIP-- -----DSPGP QV-------- ---------A SHIPPDCLVL
StGGP-1    SPE------- ---------- ---------- -DLDVTPPQ- PM-E------ -------EID ---------- -----GLN-- -------THS -T-------- ---------M VPA-------
StGGP-2    ---------- ---------- ---------- -------IN- PE-K------ ---------- ---------- -----EIP-- -----DSPGP QV-------- ---------A SHMPPDCLVL
SbGGP-1    ---------- ---------- ---------- -------EE- AS-E------ -------GKG ---------- -----TTT-- -----FAPVP VS-------- ---------P MPVAEGCLVH
SbGGP-2    ---------- ---------- ---------- ---------- ---------- ---------- ---------- ---------- ---------- ---------- ---------- ----------
SfGGP-1    ---------- ---------- ---------- ---------- ---E------ ---------- ---------- ---------- -----MPTMD SS-------L HLMGMSGVAL NGQSEHCLVL
SfGGP-2    ---------- ---------- ---------- ---------- ---S------ ---------- ---------- ---------- -----CPEWP GRT-----LF AAAGIRMVTL RRRSR-----
SppGGP-1   ---------- ---------- ---------- --------E- AG-A------ -------AGD ---------- -----SLL-- -----QTSAP PP-------- ---------A ASLFQ-----
```

```
SppGGP-2     ---------- ---------- ---------- -------DE- LM-E------ -------TTE ---------- -----SSFQ- -----RATVP PV-------- ---------A PHFPEGCLVS
TcGGP-1      LLD------- ---------- ---------- -EPDTK-TQ- SL-E------ -------IEN ---------- -----AIT-- -----KSSHR -P-------- ---------M VAGTHECLVL
TcGGP-2      ---------- ---------- ---------- -------TE- VN-QAEDKVY Q------KED ---------- -----SLCK- -----QRAAS QA-------- ---------N QRVPYDCMVL
TpGGP        ---------- ---------- ---------- -------LQ- SL-P------ -------KDD ---------- -----AVN-- -----SSTYP -T-------- ---------V VAGSQECVVL
VvGGP-1      ---------- ---------- ---------- ---------- ---------- ---------- ---------- ----GAIN-- -----NSSYP AA-------- ---------M VAGKQECLVQ
VvGGP-2      ---------- ---------- ---------- -------IR- EN-ERD---- -------REE ---------- -----DFN-- -----KPPTP RA-------- ---------T PTFA------
VcGGP        ---------- ---------- ---------- ---------- ---------- --------GA ---------- -----ALQ-- ---------- ---------- ---------- ----------
ZmGGP-E-1    ---------- ---------- ---------- -------EE- AS-D------ -------GEG ---------- -----PTA-- -----YVPVK VT-------- ---------P LPVAEGCLVL
ZmGGP-E-2    ---------- ---------- ---------- ---------- ---------- ---------- ---------- ---------- ---------- ---------- ---------- ----------
ZmGGP-1      ---------- ---------- ---------- -------EE- AS-D------ -------GEG ---------- -----PTA-- -----YVPVK VT-------- ---------P LPVAEGCLVL
ZmGGP-2      ---------- ---------- ---------- ---------- ---------- ---------- ---------- ---------- ---------- ---------- ---------- ----------
ZomGGP       ---------- ---------- ---------- -EDEVT-PG- MN-------- -------NGG ---------- -----AFL-- -----SRSSS HI-------- ---------T TPFPEGCLVL


             ....|....| ....|....| ....|....| ....|....| ....|....| ....|.
                  1210       1220       1230       1240       1250
AcGGP        H--------- ---------- ---------- ---------- ---------- ------
AcGGP-R-1    H--------- ---------- ---------- ---------- ---------- ------
AcGGP-R-2    Q--------- ---------- ---------- ---------- ---------- ------
AdGGP        H--------- ---------- ---------- ---------- ---------- ------
AeGGP        H--------- ---------- ---------- ---------- ---------- ------
ArGGP        H--------- ---------- ---------- ---------- ---------- ------
AmhGGP-1     Q--------- ---------- ---------- ---------- ---------- ------
AmhGGP-2     Q--------- ---------- ---------- ---------- ---------- ------
AncGGP-3     ---------- ---------- ---------- ---------- ---------- --ML--
AncGGP-1     H--------- ---------- ---------- ---------- ---------- ------
AncGGP-2     ---------- ---------- ---------- ---------- ---------- ------
AqcGGP       H--------- ---------- ---------- ---------- ---------- ------
AhGGP-1      Q--------- ---------- ---------- ---------- ---------- ------
AhGGP-2      N--------- ---------- ---------- ---------- ---------- ------
AlGGP-1      Q--------- ---------- ---------- ---------- ---------- ------
AlGGP-2      N--------- ---------- ---------- ---------- ---------- ------
AtGGP-1      Q--------- ---------- ---------- ---------- ---------- ------
AtGGP-2      N--------- ---------- ---------- ---------- ---------- ------
BosGGP-1     Q--------- ---------- ---------- ---------- ---------- ------
BosGGP-2     NC-------- ---------- --GAF----- ---------- ---------- ------
BdGGP-1      Q--------- ---------- ---------- ---------- ---------- ------
BdGGP-2      Q--------- ---------- ---------- ---------- ---------- ------
BdGGP-3      ---------- ---------- ---------- ---------- ---------- ------
BsGGP-1      Q--------- ---------- ---------- ---------- ---------- ------
BsGGP-2      Q--------- ---------- ---------- ---------- ---------- ------
BsGGP-3      ---------- ---------- ---------- ---------- ---------- ------
BoGGP-1      Q--------- ---------- ---------- ---------- ---------- ------
BoGGP-2      ---------- ---------- ---------- ---------- ---------- ------
BoGGP-3      ---------- ---------- --------CC T--------- ---------- ------
BrGGP-1      Q--------- ---------- ---------- ---------- ---------- ------
BrGGP-2      Q--------- ---------- ---------- ---------- ---------- ------
BrGGP-3      ---------- ---------- ---------- ---------- ---------- ------
BrGGP-4      ---------- ---------- ---------- ---------- ---------- ------
CgGGP-1      Q--------- ---------- ---------- ---------- ---------- ------
CgGGP-2      NC-------- ---------- --GAF----- ---------- ---------- ------
CrGGP-1      Q--------- ---------- ---------- ---------- ---------- ------
CrGGP-2      NC-------- ---------- --GAF----- ---------- ---------- ------
ChrGGP       ---------- ---------- ---------- ---------- ---------- ------
CcGGP-1      Q--------- ---------- ---------- ---------- ---------- ------
CcGGP-2      H--------- ---------- ---------- ---------- ---------- ------
CsGGP-1      Q--------- ---------- ---------- ---------- ---------- ------
CsGGP-2      ---------- ---------- ---------- ---------- ---------- ------
CosGGP       ---------- ---------- ---------- ---------- ---------- ------
CusGGP       ---------- ---------- ---------- ---------- ---------- ------
DcGGP-1      H--------- ---------- ---------- ---------- ---------- ------
DcGGP-2      N--------- ---------- ---------- ---------- ---------- ------
DsGGP        ---------- ---------- ---------- ---------- ---------- ------
EgGGP-1      Q--------- ---------- ---------- ---------- ---------- ------
EgGGP-2      Q--------- ---------- ---------- ---------- ---------- ------
EgGGP-3      ---------- ---------- ---------- ---------- ---------- ------
EgGGP-4      KG-------- ---------- ---------- ---------- ---------R AWLL-Q
EgGGP-5      ---------- ---------- ---------- ---------- ---------- ------
EsGGP-1      Q--------- ---------- ---------- ---------- ---------- ------
EsGGP-2      N--------- ---------- ---------- ---------- ---------- ------
FvGGP-1      Q--------- ---------- ---------- ---------- ---------- ------
FvGGP-2      QNNTGDQGNG VPPTTLVVED SSGTLPIHSA SSKGHVHIVK LLLEHYPKKK AYLVKE
FvGGP-3      ---------- ---------- ---------- ---------- ---------- ------
GmGGP-1      Q--------- ---------- ---------- ---------- ---------- ------
GmGGP-2      H--------- ---------- ---------- ---------- ---------- ------
GmGGP-3      Q--------- ---------- ---------- ---------- ---------- ------
GmGGP-4      ---------- ---------- ---------- ---------- ---------- ------
GnmGGP       Q--------- ---------- ---------- ---------- ---------- ------
GrGGP-1      Q--------- ---------- ---------- ---------- ---------- ------
GrGGP-2      Q--------- ---------- ---------- ---------- ---------- ------
GrGGP-3      Q--------- ---------- ---------- ---------- ---------- ------
KfGGP-1      Q--------- ---------- ---------- ---------- ---------- ------
KfGGP-2      Q--------- ---------- ---------- ---------- ---------- ------
KlGGP-1      Q--------- ---------- ---------- ---------- ---------- ------
KlGGP-2      Q--------- ---------- ---------- ---------- ---------- ------
KlGGP-3      Q--------- ---------- ---------- ---------- ---------- ------
LuGGP        Q--------- ---------- ---------- ---------- ---------- ------
MdGGP        Q--------- ---------- ---------- ---------- ---------- ------
MeGGP-1      Q--------- ---------- ---------- ---------- ---------- ------
MeGGP-2      Q--------- ---------- ---------- ---------- ---------- ------
MeGGP-3      H--------- ---------- ---------- ---------- ---------- ------
MpGGP        ---------- ---------- ---------- ---------- ---------- ------
MtGGP-1      Q--------- ---------- ---------- ---------- ---------- ------
MtGGP-2      Q--------- ---------- ---------- ---------- ---------- ------
MpGGP        SV-------- ---------- ---------- ---------- ---------- ----SD
MsGGP        GH-------- ---------- ---------- ---------- ---------- ------
MgGGP-1      ---------- ---------- ---------- ---------- ---------- ------
MgGGP-2      ---------- ---------- ---------- ---------- ---------- -----E
MaGGP-1      Q--------- ---------- ---------- ---------- ---------- ------
MaGGP-2      Q--------- ---------- ---------- ---------- ---------- ------
MaGGP-3      ---------- ---------- ---------- ---------- ---------- ------
MaGGP-4      ---------- ---------- ---------- ---------- ---------- ------
OtGGP        Q--------- ---------- ---------- ---------- ---------- ------
OsGGP-1      Q--------- ---------- ---------- ---------- ---------- ------
OsGGP-2      ---------- ---------- ---------- ---------- ---------- ------
OlGGP        ---------- ---------- ---------- ---------- ---------- ------
PhGGP-1      Q--------- ---------- ---------- ---------- ---------- ------
PhGGP-2      ---------- ---------- ---------- ---------- ---------- ------
PvGGP-1      Q--------- ---------- ---------- ---------- ---------- ------
PvGGP-2      Q--------- ---------- ---------- ---------- ---------- ------
PvGGP-3      ---------- ---------- ---------- ---------- ---------- ------
PhvGGP-1     Q--------- ---------- ---------- ---------- ---------- ------
PhvGGP-2     L--------- ---------- ---------- ---------- ---------- ------
PpGGP-1      ---------- ---------- ---------- ---------- ---------- ------
PpGGP-2      ---------- ---------- ---------- ---------- ---------- ------
PpGGP-3      ---------- ---------- ---------- ---------- ---------- ------
PaGGP-1      P--------- ---------- ---------- ---------- ---------- ------
```

```
PaGGP-2      P--------- ---------- ---------- ---------- ---------- ------
PaGGP-3      ---------- ---------- ---------- ---------- ---------- ------
PsGGP-1      P--------- ---------- ---------- ---------- ---------- ------
PsGGP-2      P--------- ---------- ---------- ---------- ---------- ------
PsGGP-3      ---------- ---------- ---------- ---------- ---------- ------
PtGGP-1      P--------- ---------- ---------- ---------- ---------- ------
PtGGP-2      ---------- ---------- ---------- ---------- ---------- ------
PtGGP-3      P--------- ---------- ---------- ---------- ---------- ------
PtGGP-4      P--------- ---------- ---------- ---------- ---------- ------
PtGGP-5      ---------- ---------- ---------- ---------- ---------- ------
PotGGP-1     H--------- ---------- ---------- ---------- ---------- ------
PotGGP-2     Q--------- ---------- ---------- ---------- ---------- ------
PotGGP-3     Y--------- ---------- ---------- ---------- ---------- ------
PrpGGP       Q--------- ---------- ---------- ---------- ---------- ------
RcGGP-1      Q--------- ---------- ---------- ---------- ---------- ------
RcGGP-2      Q--------- ---------- ---------- ---------- ---------- ------
RcGGP-3      H--------- ---------- ---------- ---------- ---------- ------
SpGGP-1      Q--------- ---------- ---------- ---------- ---------- ------
SpGGP-2      H--------- ---------- ---------- ---------- ---------- ------
SpGGP-3      H--------- ---------- ---------- ---------- ---------- ------
SmGGP-1      V--------- ---------- ---------- ---------- ---------- ------
SmGGP-2      ---------- ---------- ---------- ---------- ---------- ------
SmGGP-3      ---------- ---------- ---------- ---------- ---------- ------
SiGGP-1      Q--------- ---------- ---------- ---------- ---------- ------
SiGGP-2      ---------- ---------- ---------- ---------- ---------- ------
SvGGP-1      Q--------- ---------- ---------- ---------- ---------- ------
SvGGP-2      ---------- ---------- ---------- ---------- ---------- ------
SlGGP-1      ---------- ---------- ---------- ---------- ---------- ------
SlGGP-2      Q--------- ---------- ---------- ---------- ---------- ------
StGGP-1      ---------- ---------- ---------- ---------- ---------- ------
StGGP-2      Q--------- ---------- ---------- ---------- ---------- ------
SbGGP-1      Q--------- ---------- ---------- ---------- ---------- ------
SbGGP-2      ---------- -----L---- ---------- ---------- ---------- ------
SfGGP-1      QV-------- ---------- ---------- ---------- ---------- ------
SfGGP-2      ---------- ---------- ---------- ---------- ---------- ------
SppGGP-1     ---------- ---------- ---------- ---------- ---------- ------
SppGGP-2     Q--------- ---------- ---------- ---------- ---------- ------
TcGGP-1      Q--------- ---------- ---------- ---------- ---------- ------
TcGGP-2      Q--------- ---------- ---------- ---------- ---------- ------
TpGGP        Q--------- ---------- ---------- ---------- ---------- ------
VvGGP-1      Q--------- ---------- ---------- ---------- ---------- ------
VvGGP-2      ---------- ---------- ---------- ---------- ---------- ------
VcGGP        ---------- ---------- ---------- ---------- ---------- ------
ZmGGP-E-1    P--------- ---------- ---------- ---------- ---------- ------
ZmGGP-E-2    ---------- -----LG--- ---------- ---------- ---------- ------
ZmGGP-1      P--------- ---------- ---------- ---------- ---------- ------
ZmGGP-2      ---------- -----LG--- ---------- ---------- ---------- ------
ZomGGP       Q--------- ---------- ---------- ---------- ---------- ------
```
